# Detection of Protein–Protein Interactions in *Escherichia coli* With Single Molecule Sensitivity

**DOI:** 10.1002/advs.202510093

**Published:** 2026-04-10

**Authors:** Marilyne Davi, Daniel Ladant

**Affiliations:** ^1^ Institut Pasteur Université Paris Cité CNRS UMR 3528 Paris France; ^2^ Unité De Biochimie des Interactions Macromoléculaires Département De Biologie Structurale Et Chimie CNRS UMR 3528 Paris France

**Keywords:** cyclic AMP signaling, protein–protein interactions, single molecule sensitivity, two‐hybrid

## Abstract

Protein–protein interactions are central in all biological processes. Methods capable of detecting interactions within living, intact cells have been particularly useful to identify and characterize protein interaction networks. We describe here an exquisitely sensitive regulatory circuit that can detect in bacteria, protein–protein interaction with single‐molecule sensitivity. This approach involves the interaction‐mediated reconstitution of a cyclic AMP signaling cascade in *Escherichia coli* taking advantage of the high catalytic activity of the adenylate cyclase (AC) from *Bordetella pertussis* upon activation by its natural activator, calmodulin (CaM). We show that less than one complex of interacting hybrid proteins per cell on average, is enough to confer a selectable trait to the host. This exquisitely sensitive adenylate cyclase hybrid (ESACH) system allows for direct selection, in living bacteria, of ligands exhibiting high affinity for given targets or for studying interactions involving toxic proteins. The extreme sensitivity of the AC/CaM/cAMP signaling cascade may thus be harnessed to interrogate biological processes with single‐molecule resolution in live bacteria and could be exploited to design novel synthetic regulatory networks operating at, or even below, the theoretical threshold limit of one molecule per cell.

## Graphical abstract

This article describes a regulatory circuit in *Escherichia coli* able to detect protein–protein interactions with exquisite sensitivity. The interaction between two hybrid proteins fused to Bordetella pertussis adenylate cyclase and its activator calmodulin triggers a potent cyclic AMP signaling cascade. On average, a single hybrid protein complex per cell is sufficient to confer a selectable trait to the host.

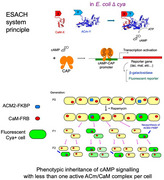

**Abbreviations**
AC: *Bordetella pertussis* adenylate cyclase catalytic domain
ACM1: ACM247 = AC mutant with a Leu‐Gln insertion between residues 247 and 248
ACM2: ACM335 = AC mutant with a Cys‐Ser insertion between residues 335 and 336
BACTH: Bacterial Adenylate Cyclase Two‐Hybrid
CaM: calmodulin (human)
cAMP: cyclic AMP
CAP (or CRP): catabolite activator protein
ESACH: Exquisitely Sensitive Adenylate Cyclase Hybrid System
FKBP: FK506‐binding protein
FRB: FKBP‐rapamycin binding domain
GFP: green fluorescent protein
IPTG: Isopropyl‐β‐D‐thiogalactopyranoside
LB: Luria‐Bertani broth
Mab: monoclonal antibody
PPI: protein–protein interactions
RBS: Ribosome Binding Sequence
scFv: single‐chain antibody fragment
SD: standard deviation
T25, T18: AC fragments
V_1K_
: camelidae variable heavy chain antibody fragment 3K1K
V_9A_
: camelidae variable heavy chain antibody fragment 3G9A
V_H_H: camelidae variable heavy chain antibody fragment
WB: Western Blot
Xgal: 5‐Bromo‐4‐chloro‐3‐indolyl‐β‐D‐galactopyranoside
Zip: GCN4 leucine zipper motif

## 1 Introduction

Most cellular processes are driven by molecular interactions and the study of protein interaction networks represents an essential step to understanding biological systems. Methods capable of detecting protein–protein interactions (PPI) within living, intact cells have been particularly useful to identify and characterize the “interactome”, a cornerstone of systems biology. Most of these methods are based on the co‐expression, in the same cell, of two (or more) hybrid proteins that, upon interaction, produce a phenotypic and/or selective trait. This approach was pioneered by Fields and Song, who developed the yeast two‐hybrid system (or interaction trap), in which the putative interaction partners are co‐expressed as fusions with two moieties of a transcriptional activator [1]. Association of the two hybrid proteins restores the activity of the split transcriptional activator and leads to the expression of specific reporter genes. Similar approaches have later been designed in which split enzymes or fluorescent/luminescent proteins are used as reporter systems to select or image in vivo protein–protein interactions in various hosts, such as yeast, mammalian or bacterial cells [2, 3, 4, 5, 6, 7, 8, 9, 10, 11, 12, 13, 14, 15, 16, 17, 18, 19, 20, 21, 22, 23].

We previously elaborated a bacterial two‐hybrid system, BACTH (Bacterial Adenylate Cyclase Two‐Hybrid) that is based on the interaction‐mediated reconstitution of a cyclic AMP (cAMP) signaling cascade in *Escherichia coli* [24]. In this system, the proteins of interest are genetically fused to two complementary fragments, T25 and T18, from the catalytic domain of *Bordetella pertussis* adenylate cyclase (AC), and co‐expressed in an *E. coli cya* strain (i.e. deficient in its endogenous adenylate cyclase). Association of the two hybrid proteins results in functional complementation between the separately inactive T25 and T18 fragments leading to cAMP synthesis. In *E. coli*, cAMP binds to the catabolite activator protein (CAP or CRP) and triggers the transcriptional activation of catabolic operons, such as lactose or maltose, thus yielding a characteristic phenotype. This system has been extensively used to reveal a wide variety of interactions between bacterial, eukaryotic, or viral proteins, occurring at various subcellular locations, e.g. cytosol, membrane or DNA level [25, 26, 27, 28].

Here, we describe a novel architecture for the AC‐based two‐hybrid screen that permits detection of PPI in bacteria with an extreme sensitivity. This approach takes advantage of the high catalytic potency of *B. pertussis* AC upon activation by its natural activator, the eukaryotic calcium‐sensor, calmodulin (CaM) [29]. With this new design, we found that, on average, less than one complex of interacting hybrid proteins per cell is enough to confer a selectable trait to the host. This exquisitely sensitive adenylate cyclase hybrid (ESACH) system should be particularly appropriate for direct in vivo selection of ligands exhibiting high affinity for given targets or for studying PPI involving toxic proteins. The ultimate sensitivity provided by the AC/CaM/cAMP signaling cascade that can potentially detect one active enzyme complex per bacterium, might also be exploited to engineer novel synthetic regulatory networks operating at, or even below, the theoretical threshold limit of one molecule per cell.

## 2 Results

### 2.1 Design of an Exquisitely Sensitive Adenylate Cyclase Hybrid (ESACH) System

Our previously described AC‐based two‐hybrid system [24] relies on the interaction‐mediated reconstitution of enzymatic activity from two complementary fragments of *B. pertussis* AC. In the absence of its activator calmodulin (CaM), AC exhibits a k_cat_ of about 1‐2 s^−1^, and therefore few hundreds of active hybrid protein complexes per bacteria are required to produce enough cAMP to confer a Cya*^+^* phenotype to the *E. coli Δcya* host cells. Upon binding to CaM, AC is activated more than a 1000‐fold [29] to reach a turnover number of about 2000 s^−1^. We reasoned that by taking advantage of the full catalytic potency of AC upon activation by CaM one should drastically increase the sensitivity of this genetic assay, so that a single active AC/CaM complex per cell might potentially be sufficient to confer a selectable trait to the bacterial host.

**FIGURE 1:**
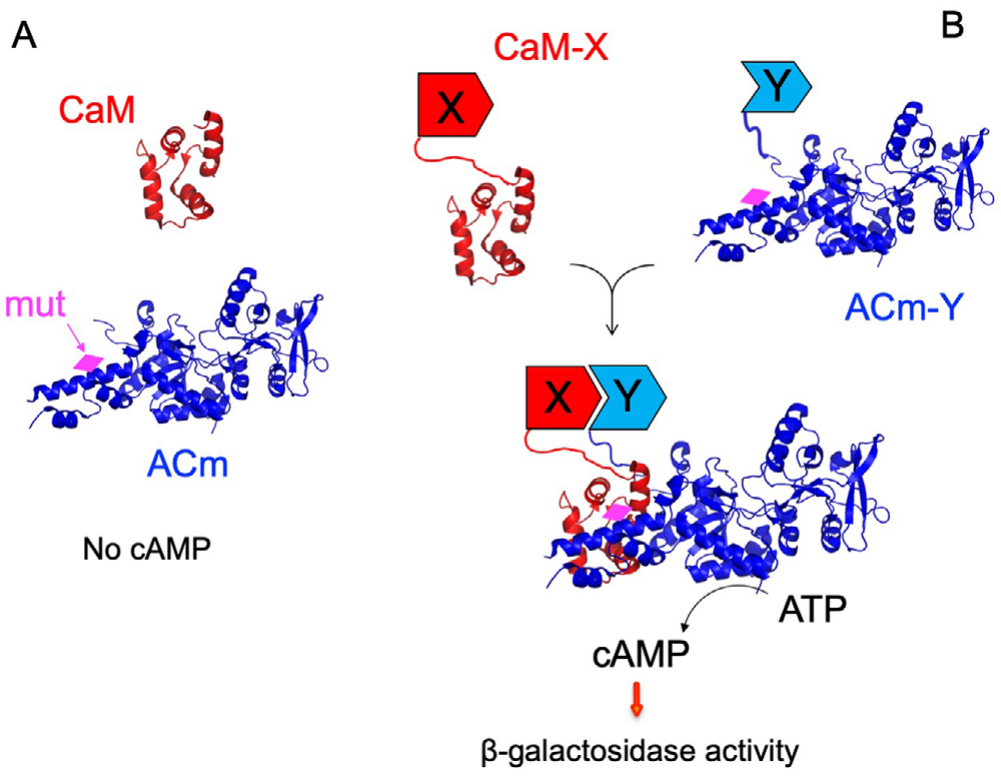
Principle of the exquisitely sensitive adenylate cyclase hybrid (ESACH) system. (A) The catalytic domain of *B. pertussis* adenylate cyclase (blue) is modified (ACm) in its CaM‐binding domain (magenta diamond) to decrease its affinity for CaM (red). When expressed at low level in *E. coli ΔcyaA*, CaM cannot activate ACm and there is no cAMP synthesis. (B) When CaM and ACm are fused to two interacting proteins, X and Y, they are brought into close proximity, and CaM can activate ACm to produce cAMP (right). Cyclic AMP then binds to the catabolite gene activator protein, CAP, and the cAMP/CAP complex can stimulate the transcription of the catabolite genes, such as the lactose operon or the maltose regulon.

In the novel architecture explored here, named ESACH for Exquisitely Sensitive Adenylate Cyclase Hybrid, the two proteins of interest are separately fused to AC and CaM and co‐expressed in an *E. coli Δcya* strain (Figure 1). To render the AC activation dependent upon the association of the hybrid proteins, AC was engineered to reduce its naturally very high affinity for CaM (the CaM concentration required for half‐maximal activation, K_1/2_, is about 0.1–0.2 nM in the presence of calcium [30]) by introducing appropriate mutations, so that when both the modified AC and CaM are expressed alone at low level in a *E. coli Δcya* strain, they cannot spontaneously associate. Among the various modifications known to decrease CaM affinity, we chose two, ACM1 and ACM2, that result from two‐amino acid insertions within the T18 moiety of AC: Leu‐Gln between residues 247 and 248 in ACM1, and Cys‐Ser between residues 335 and 336 in ACM2 [30]. These insertions were previously shown to decrease CaM affinity by more than 3 000 and 50‐100‐fold, respectively [30]. These two‐codon insertion mutations are expected to be less prone to reversion toward a wild‐type, high‐affinity phenotype (as it would require a precise excision of the 6 inserted nucleotides) than a single point mutation replacing a critical residue involved in CaM‐binding [31]. As a model system of interacting proteins, we used the antigen‐binding fragment from camelidae heavy chain antibodies, or “nanobodies”, V_H_H # 3G9A and V_H_H # 3K1K, here called V_9A_ and V_1K_, respectively, that interact with high affinity (K_D_ ≈ 0.5 nM) with the green fluorescent protein (GFP) as reported by Kirchhofer et al. [32].

### 2.2 Detection of Active Hybrid AC/CaM Complexes in Living Bacteria

**FIGURE 2:**
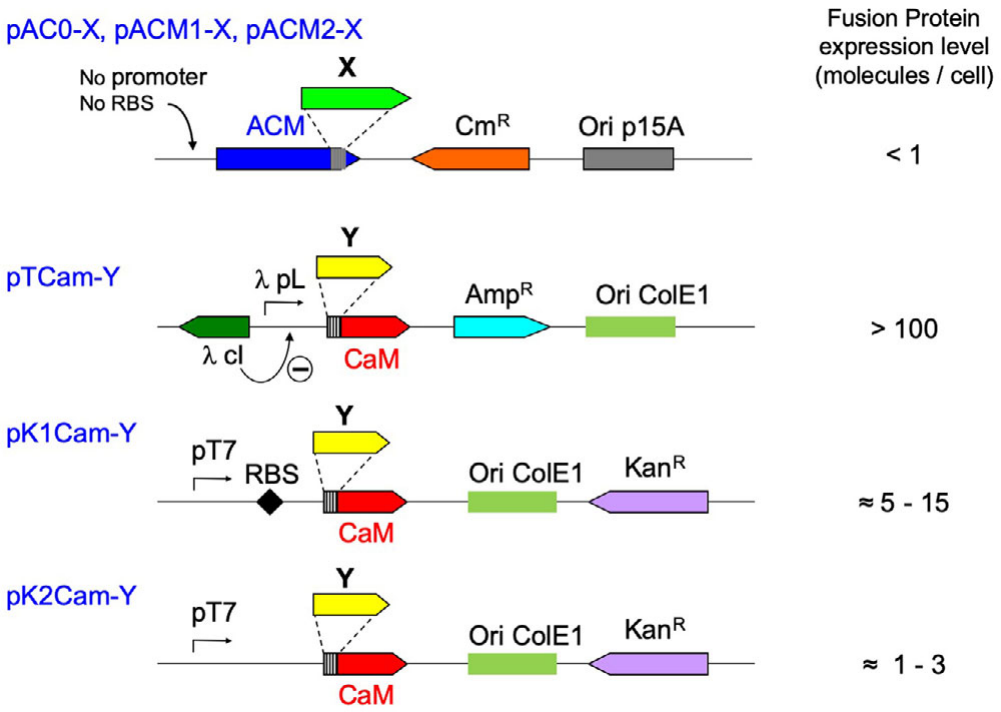
Schematic representation of ESACH plasmids. The colored boxes represent the ORFs of different genes, with the arrows indicating the direction of transcription/translation. The hatched boxes correspond to the multicloning site sequences (MCS) fused to the Cter of ACM or N‐ter of CaM. X and Y are the protein of interest fused to ACM and CaM. The origins of replication of the plasmids are indicated by shaded boxes. λcI corresponds to the thermosensitive repressor cI^857^ that strongly represses the λ promoter pL at low temperature (30°C or below), pT7 to the T7 promoter (note that DHM1 does not express any T7 polymerase) and RBS to the ribosome binding site. For each plasmid, the relative expression level of the ACM or CaM fusion proteins_,_ expressed as number of molecules per bacterial cell, and estimated by western blot analysis, is given on the right.

**FIGURE 3:**
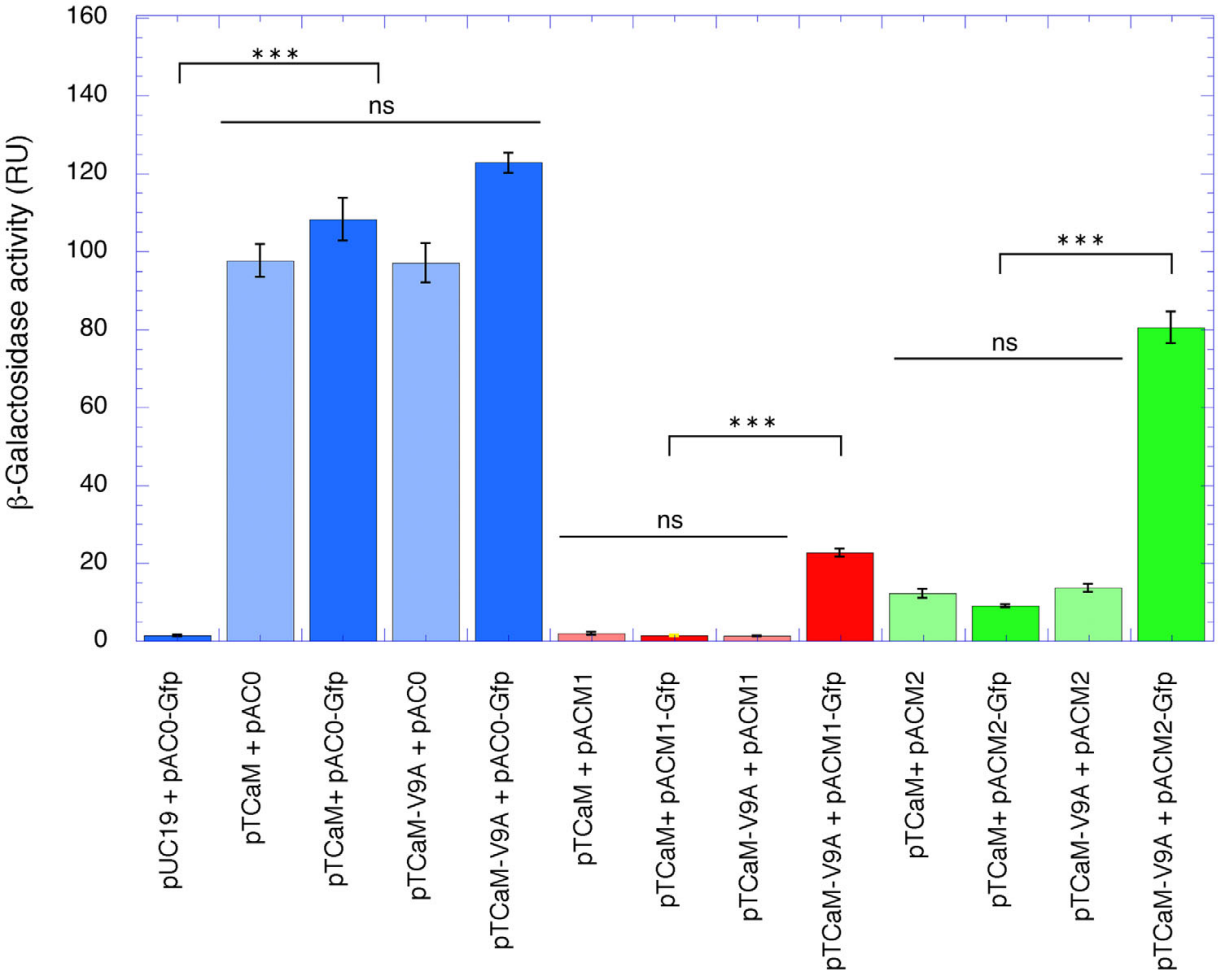
β‐galactosidase assays of protein–protein interaction with ESACH system. DHM1 cells were transformed with the indicated plasmids. For each type of transformants, the bars represent mean ± SD of β‐galactosidase activities, measured as described in Material and Methods, on 6‐8 independent colonies (technical replicates) grown overnight at 30°C in LB medium supplemented with IPTG and antibiotics. Statistical differences were established by one‐way ANOVA followed by Tukey's multiple comparison test. ***p < 0.001, n.s., non‐significant. Similar results were obtained in 3 independent experiments (biological replicates), each performed on distinctly transformed cells.

Different expression systems were explored in order to express AC in *E. coli* at the minimal possible level yet enough to confer a selectable Cya*+* phenotype to an *E. coli Δcya* strain. Among them, we selected an expression vector (pAC0) derived from the low‐copy plasmid pACYC184, in which all transcriptional and translational control sequences upstream of the AC open reading frame (residues 1 to 399 from *B. pertussis* CyaA) were deleted (Figure 2; Table S1 and Appendix 1). Expression in the *E. coli Δcya* strain DHM1, of the wild‐type AC from this vector (pAC0) or AC fused to GFP (pAC0‐Gfp) was able to restore a Cya*^+^* phenotype—as detected by β‐galactosidase assays in liquid cultures (Figure 3; Table 1), measurements of cAMP on total bacterial extract (Table 1) or formation of blue colonies on LB X‐gal plates (Figure S1)—provided the host cells also harbored a compatible plasmid expressing CaM (pTCam, a pUC19 derived vector with a CaM gene under control of a thermosensitive λ promoter [33]).

**TABLE 1:** In vivo complementation between AC and CaM fusions.

	CaM Plasmids	CaM fusion levels	AC plasmids	Phenotype on LB/Xgal	ß gal (Rel. Units)	cAMP (nmol/mg)
1	pDL1312	no	pAC0‐Gfp	White	1 ± 0.5	< 0.1
2	pTCam	High CaM Expression	pAC0‐Gfp	Blue	106 ± 11	> 250
3			pACM1‐Gfp	White	1 ± 0.4	< 0.1
4			pACM2‐Gfp	Blue	8.0 ± 1	NT
5	pTCam‐V_9A_	High CaM‐V_9A_ Expression	pAC0‐Gfp	Blue	107± 15	> 250
6			pACM1‐Gfp	Blue	26 ± 2	> 50
7			pACM2‐Gfp	Blue	78 ± 7	> 60
8	pK1Cam‐V_9A_	Moderate CaM‐V_9A_ Expression	pAC0‐Gfp	Blue	102 ± 14	230 ± 25
9			pACM1	White	1 ± 0.3	< 0.1
10			pACM1‐Gfp	Pale Blue	4 ± 1	1.4 ± 0.5
11			pACM2	White	2 ± 0.4	NT
12			pACM2‐Gfp	Blue	57 ± 8	60 ± 10
13			pACM2‐Fkbp	White	2 ± 0.5	< 0.1
14	pK2Cam‐V_9A_	Low CaM‐V_9A_ Expression	pAC0‐Gfp	Blue	76 ± 6	160 ± 15
15			pACM1	White	1 ± 0.4	< 0.1
16			pACM1‐Gfp	Pale Blue	4 ± 1	0.25 ± 0.1
17			pACM2	White	2 ± 0.6	NT
18			pACM2‐Gfp	Blue	23 ± 5	11 ± 2
19			pACM2‐Fkbp	White	2 ± 0.3	< 0.1
20	pK1Cam‐V_1K_	Moderate CaM‐V_1K_ Expression	pACM2	NT	2 ± 0.4	NT
21			pACM2‐Gfp	NT	74 ± 7	NT
22			pACM2‐Fkbp	NT	2 ± 0.5	NT
23	pK1Cam‐Frb	Low CaM‐V_1K_ Expression	pACM2‐Gfp	White	1 ± 0.5	NT
24			pACM1‐Fkbp	White	2 ± 0.5	NT
25			pACM2‐Fkbp	White	2 ± 0.3	NT
26			pACM1‐Fkbp (+ Rap)	NT	9 ± 2.1	NT
27			pACM2‐Fkbp (+ Rap)	NT	48 ± 6	NT
28	pK2Cam‐Frb	Low CaM‐Frb Expression	pACM2‐Gfp	White	1 ± 0.3	NT
29			pACM2‐Fkbp	White	2 ± 0.4	NT
30			pACM2‐Fkbp (+ Rap)	NT	40 ± 6	NT

DHM1 bacteria were transformed with the indicated plasmids and plated on LB agar plate supplemented with appropriate antibiotics, IPTG, and X‐gal and grown at 30°C for 36 hrs in the presence of 0.5 mM isopropyl β‐D‐thiogalactoside (X‐gal) plus appropriate antibiotics (Figure S1). For each type of transformants, 8 colonies were picked up and grown overnight at 30°C in LB medium supplemented with IPTG and antibiotics. The β‐galactosidase activities were measured as described in Material and Methods. The values represent mean ± SD of β‐galactosidase activities, expressed in relative units, RU, (calculated as indicated in Material and Methods), on the 6‐8 independent colonies. Cyclic AMP levels, expressed in nmol cAMP/mg bacterial dry weight, were determined on the same overnight cultures as described previously [21]. When indicated (+ Rap), 10 µM Rapamycin were added to the liquid culture. Plasmid pDL1312, used here as a control, is a pTCam parental plasmid that expresses neurocalcin instead of CaM [72]. NT: not tested. Similar results were obtained in 3 independent experiments, each performed on distinctly transformed cells.

When the ACM1 variant, either alone or as a fusion to GFP (encoded by plasmids pACM1 and pACM1‐Gfp respectively) was co‐expressed with CaM in DHM1, it failed to restore a Cya^+^ phenotype (Figure 3; Table 1). However, when the pACM1‐Gfp plasmid was co‐transformed with pTCam‐V_9A_ (Figure 2), a pTCaM derivative that expresses CaM as a fusion with the V_9A_ nanobody, the co‐transformants exhibited a clear Cya^+^ phenotype, although the β‐galactosidase expression levels (and cAMP in bacterial culture) were much lower than in cells co‐expressing CaM and the wild‐type AC‐GFP (Figure 3; Table 1). We concluded that CaM‐V_9A_ but not CaM alone, could activate in vivo the ACM1‐GFP variant as a result of the specific interaction between the V_9A_ and the GFP moieties.

**FIGURE 4:**
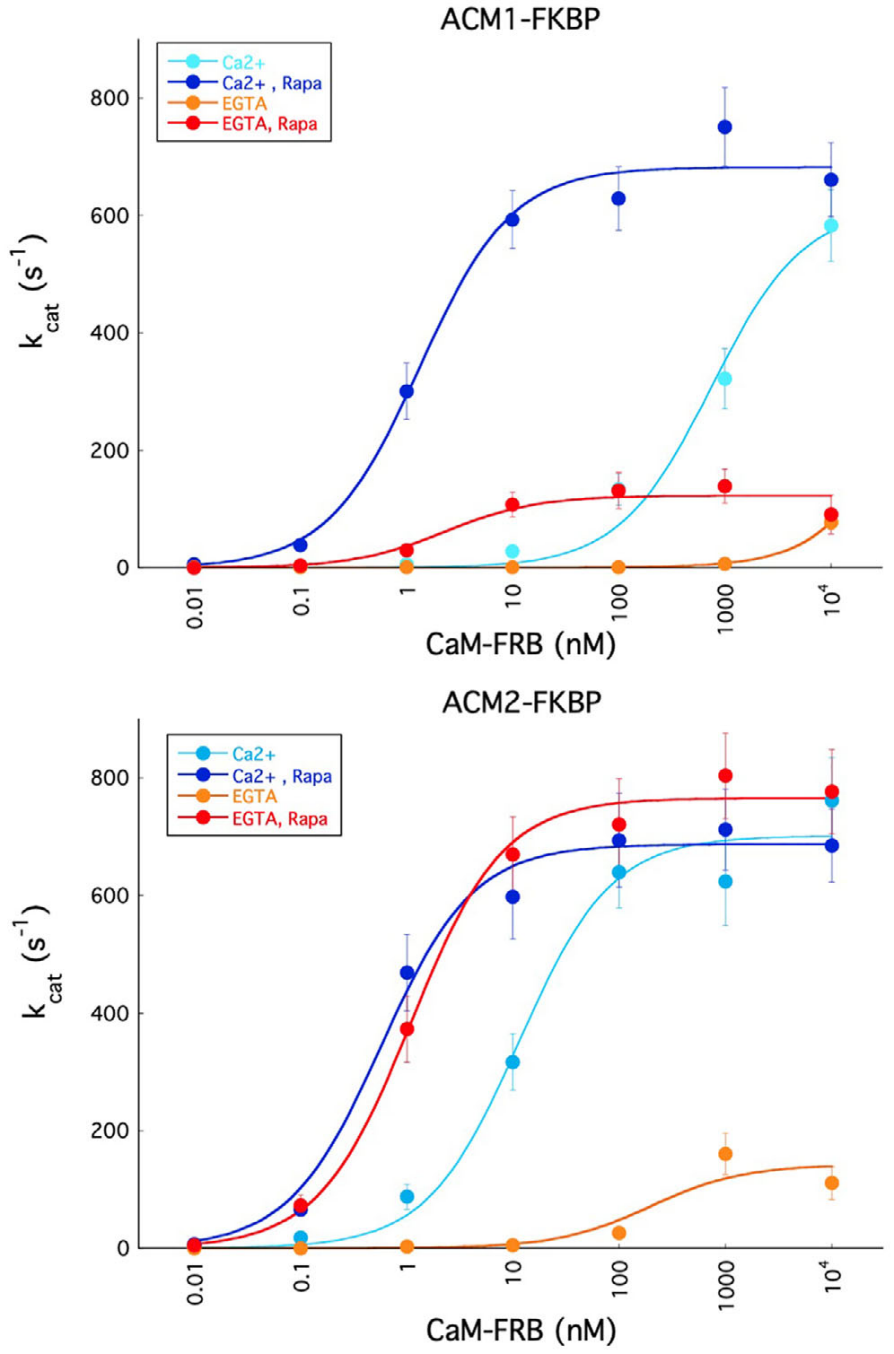
In vitro assays of the rapamycin‐induced activation of ACM‐FKBP by CaM‐FRB. Purified ACM1‐FKBP (upper panel) and ACM2‐FKBP (lower panel) (both at 0.5 nM) were pre‐incubated for 10 min at 30°C with the indicated concentrations of CaM‐FRB in a reaction medium containing either 0.1 mM calcium (Ca^2+^) or 0.1 mM calcium and 1 mM EGTA (EGTA, i.e. no free calcium) and in the presence of 5 µM Rapamycin (Rapa) when indicated. Reactions were initiated by adding 2 mM ATP and carried out for 20 min. Cyclic AMP was then measured spectrophotometrically as described in Material and Methods. The AC activities (expressed in k_cat_ = mol of cAMP produced per mol of enzyme per sec) at the indicated CaM‐FRB concentrations were fitted to equation: A = (A_Max_ x [CaM]) / (K_D_ + [CaM]) with A_Max_ = maximal activities at saturating CaM‐FRB and K_D_ = the CaM‐FRB concentration at half‐maximal activation. The deduced A_Max_ and K_D_ values are listed in Table S2. Similar results were obtained in 4 independent experiments (biological replicates), carried out with distinct ACM1‐FKBP, ACM2‐FKBP, and CaM‐FRB preparations.

The second AC variant, ACM2, similarly expressed as a GFP fusion (from plasmid pACM2‐GFP) also conferred a robust Cya^+^ phenotype to DHM1 when co‐transformed with pTCam‐V_9A,_ as anticipated, but also and unexpectedly, when co‐transformed with pTCaM (or when expressed alone together with CaM or CaM‐V_9A_; Figure 3; Table 1). We hypothesized that the CaM protein level—due to the residual transcription from the cI^857^‐repressed λ promoter (Figure 2)—was high enough to spontaneously activate the ACM2 variant that has a higher affinity for CaM than ACM1 ([30] and Figure 4). We therefore tested alternative expression systems in order to reduce the level of expression of CaM fusions. We constructed two plasmids, pK1Cam‐V_9A_ and pK2Cam‐V_9A_, in which the CaM‐V_9A_ fusion was expressed under the control of a T7 promoter with or without an RBS sequence, respectively (Figure 2; Table S1 and Appendix 1). As the DHM1 cells have no T7 polymerase, the CaM fusion was expected to be expressed at very low level. These plasmids, when co‐transformed with pAC0‐GFP or with pACM2‐GFP, conferred a robust Cya^+^ phenotype to DHM1 cells although the cAMP and β‐galactosidase levels were lower with the latter as compared to the former (Table 1).

When pK1Cam‐V_9A_ or pK2Cam‐V_9A_ were co‐transformed with pACM1‐Gfp, the transformants showed much lower β‐galactosidase and cAMP levels, resulting in a barely detectable Cya^+^ phenotype, likely because of the lower specific activity of the ACM1 variant as compared to wild‐type AC or ACM2 ([30] and Figure 4). Yet, measurements of β‐galactosidase activities unambiguously revealed that ACM1‐GFP, but not ACM1, was activated by the CaM‐V_9A_ hybrid in bacteria (Table 1; Figure S2). More importantly, a Cya^−^ phenotype was obtained upon co‐transformation of pK1Cam‐V_9A_ or pK2Cam‐V_9A_, with pACM2 that expresses ACM2 alone (Table 1). Hence, the expression levels of the CaM‐V_9A_ fusion obtained with these new plasmids now appeared to be appropriate to allow the selective activation of the ACM1‐GFP or ACM2‐GFP fusions but not of the unfused ACM1 or ACM2. We similarly tested another camelidae V_H_H (V_H_H # 3K1K), here noted V_1K_^32^, that also exhibits a high affinity for GFP: the CaM‐V_1K_ fusion was also found to selectively activate in vivo the ACM2‐GFP fusion but not ACM2 (Table 1).

The ESACH system was further characterized by analyzing the rapamycin‐induced interaction between the FK506‐binding protein, FKBP and the FKBP‐rapamycin binding domain, FRB, that binds with high affinity to FKBP in the presence of rapamycin [34]. Plasmids expressing ACM1 or ACM2 fused to FKBP, pACM1‐Fkbp pACM2‐Fkbp respectively, and CaM fused to FRB, pK1Cam‐Frb and pK2Cam‐Frb (Table S1 and Appendix 1), were constructed and tested in complementation assays in DHM1 as above. As shown in Table 1, the CaM‐FRB fusions, produced by the pK1Cam‐Frb or pK2Cam‐Frb plasmids, were able to specifically activate the ACM2‐FKBP only when the cells were grown in the presence of rapamycin. Activation of ACM1‐FKBP by CaM‐FRB could also be detected in cells grown in the presence of rapamycin albeit with a lower efficiency as expected (Table 1; Figure S2). Furthermore, the CaM‐FRB fusion did not activate the ACM2‐GFP hybrid and conversely the CaM‐V_9A_ or CaM‐V_1K_ fusions did not activate the ACM2‐FKBP hybrid (Table 1).

Altogether, these data indicate that the CaM fusions produced by pK1Cam or pK2Cam plasmids could activate in vivo the ACM1 or ACM2 hybrids in a highly selective manner, dictated by the specific association between the protein modules appended to CaM and ACM variants. In additional experiments, we also showed that CaM variants (or fragments) with decreased affinity for AC [35, 36] can also be used as activating modules of AC or ACM2 (see Supplementary note in Supporting Information)

### 2.3 In Vitro Assays of the Interaction‐Mediated Activation of Modified ACs

To corroborate the above results at the molecular level, we characterized in vitro the interaction‐mediated activation of modified AC‐fusions by cognate CaM‐fusions. The ACM1 and ACM2‐FKBP fusions as well as the CaM‐FRB fusion were overexpressed in *E. coli* and purified to near homogeneity (see Supporting Information and Figure S3). The activity of the AC fusions in the presence of increasing concentrations of CaM‐FRB was then assayed in vitro [34, 37] either in the presence or in the absence of calcium or rapamycin. As shown in Figure 4, in the presence of calcium and absence of rapamycin (ie when FKBP and FRB do not associate), half‐maximal activations (equivalent of K_eq_) were obtained at CaM‐FRB concentrations of ∼ 750 nm for ACM1‐FKBP and ∼ 10–15 nM for ACM2‐FKBP (Table S2), in agreement with earlier measurements of the activation of ACM1 or ACM2 by native CaM [30]. Upon addition of rapamycin, both AC fusions were half‐maximally activated at about 1–2 nM CaM‐FRB, a concentration close to the FKBP/rapamycin/FRB equilibrium constant [34]. Most notably, in the absence of calcium, that is, in conditions prevailing in the bacterial cytosol, and without rapapmycin, ACM1‐FKBP was barely activated by CaM‐FRB, while ACM2‐FKBP was partially activated at CaM‐FRB concentrations above 100 nm. This can explain why a Cya^+^ phenotype was observed when DHM1 was co‐transformed with pACM2‐GFP and pTCam as this plasmid likely expressed high CaM levels in bacteria. In the absence of calcium and in the presence of rapamycin ACM2‐FKBP was fully activated by CaM‐FRB, with a half‐stimulating concentration of about 1 nm as above. Interestingly, in the same conditions, ACM1‐FKBP was also half‐maximally activated at about 2.5 nM CaM‐FRB, but its maximal activity reached only about 15% of that found in the presence of calcium. We hypothesize that in the presence of rapamycin, ACM1‐FKBP/rapamycin is fully saturated by CaM‐FRB at concentrations above 10 nm, but, in this complex, the Ca^2+^‐free CaM moiety can only transiently associate (e.g. about 15 % of the time) with the ACM1 moiety to produce an active enzyme, as a result of the strongly destabilizing mutation introduced in ACM1. All together, these experiments directly corroborated the stringent interaction‐mediated activation of the ACM fusions observed in bacteria. They also explained the lower efficiency of complementation observed in bacteria with the ACM1 variant as compared with ACM2.

### 2.4 Detection of Interaction Between Membrane‐Associated Proteins

**FIGURE 5:**
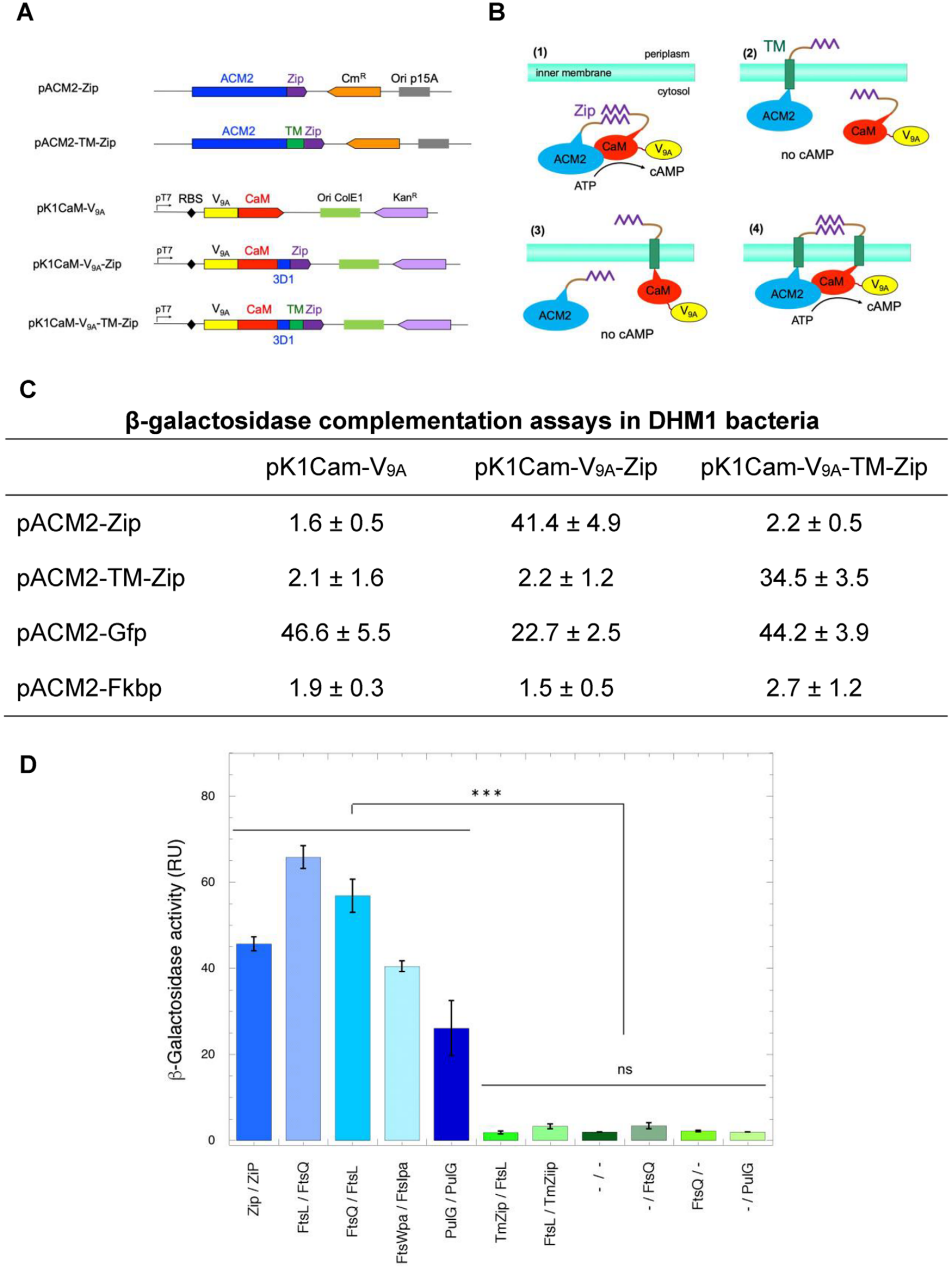
Detection of interactions between membrane‐associated proteins with the ESACH system. A) Schematic representation of plasmids expressing cytosolic or membrane‐associated ACM2 & CaM hybrid proteins. The colored boxes represent the ORFs of the different genes, with the arrow indicating the direction of transcription/translation. ACM2 is in blue, the leucine zipper motif (Zip) in violet, the OppB transmembrane segment (TM) in green, the V_9A_ nanobody is in yellow, and CaM is in red. The 3D1 blue rectangle in CaM plasmids correspond to the very C‐terminal segment of AC containing the 3D1 epitope that was inserted during cloning of the leucine zipper motif. The chloramphenicol (Cm^R^) and kanamycin (Kan^R^) resistant markers as well as the p15A and ColE1 origins of replication are indicated by colored rectangles. B) Schematic representation of the expected topology of the different ACM2 and CaM hybrid proteins relative to the inner membrane (light green bar); (1): ACM2‐Zip / V_9A_‐CaM‐Zip; (2): ACM2‐TM‐Zip / V_9A_‐CaM‐Zip; (3): ACM2‐Zip / V_9A_‐CaM‐TM‐Zip; (4): ACM2‐TM‐Zip / V_9A_‐CaM‐TM‐Zip. C and D) Complementation assays. DHM1 cells were transformed with plasmids encoding the indicated pairs of hybrids (labeled in D as: pACM2 hybrid / pK1Cam hybrid;—= no fusion) and plated on LB agar plus IPTG and X‐gal and grown at 30°C for 36 hrs. The β‐galactosidase activities (expressed in Relative Units) were measured on liquid cultures grown overnight at 30°C in LB plus appropriate antibiotics and IPTG as described in Material and Methods. For each couple of transformants, the values (in C) or bars (in D) represent mean ± SD of β‐galactosidase activities, measured on 6‐8 independent colonies (technical replicates). Statistical differences were established by one‐way ANOVA followed by Tukey's multiple comparison test. ***p < 0.001, ns, non‐significant. Similar results were obtained in 3 independent experiments (biological replicates), each performed on distinctly transformed cells.

We then explored the capacity of the ESACH system to detect interactions between membrane‐associated and/or periplasmic proteins. For this, we constructed plasmids (Figure 5A; Table S1 and Appendix 1) expressing ACM2 or CaM‐V_9A_ fused to a trans‐membrane (TM) segment (the first TM from the *E. coli* OppB oligopeptide transporter subunit [27]) followed by the leucine zipper dimerization domain of GAL4 (Zip). The resulting fusions, ACM2‐TM‐zip and CaM‐V_9A_‐TM‐zip, should insert into the membrane with their Zip motif localized in the periplasm (see Figure 5B). As controls, ACM2 and CaM‐V_9A_ were also expressed as fusions to the leucine zipper motif without the TM domain (i.e. localized in the cytosolic compartment, Figure 5B). As shown in Figure 5C, the membrane‐associated ACM2‐TM‐Zip and CaM‐V_9A_‐TM‐Zip hybrids efficiently associated through their leucine zipper motifs localized in the periplasm. The interaction signal was similar to that detected between the hybrid proteins ACM2‐Zip and CaM‐V_9A_‐Zip that have a cytosolic leucine zipper (Figure 5B). Importantly, ACM2‐TM‐Zip did not interact with CaM‐V_9A_‐Zip nor ACM2‐Zip with CaM‐V_9A_‐TM‐Zip (Figure 5C). This indicates that the leucine zipper motif of the TM‐Zip constructs was properly addressed to the periplasm by the OppB TM domain. In addition, both the membrane‐anchored CaM‐V_9A_‐TM‐Zip and the cytosolic CaM‐V_9A_‐Zip could efficiently interact via their cytoplasmic‐localized V_9A_ module (Figure 5B), with ACM2‐GFP (but not with ACM2‐FKBP as expected) (Figure 5C). These data thus indicate that the ESACH system can efficiently report interactions involving integral membrane proteins or interactions between an integral membrane protein and a cytosolic protein. In addition, they show that CaM can be dually tagged at both its N and C termini to yield fusions that can specifically activate two distinct ACM‐fusions.

We further tested interactions between several native integral membrane proteins previously shown to associate (Figure 5D) in the membrane, including: (i) the small bitopic proteins, FtsQ and FtsL from *E. coli*, that associate with FtsB, to form a trimeric complex essential for cell division [38]; (ii) the bitopic protein FtsI and multipass membrane protein FtsW from *Pseudomonas aeruginosa* forming a tight complex involved in septal peptidoglycan synthesis [39]; (iii) the major pilin subunit PulG that oligomerizes in the membrane to form the pseudo pilus of the type II secretion systems (T2SS) of *Klebsiella oxytoca* [40]. Overall, these results show that the ESACH system is applicable to characterize interactions between integral membrane‐proteins.

### 2.5 Expression Levels of Hybrid Proteins

**FIGURE 6:**
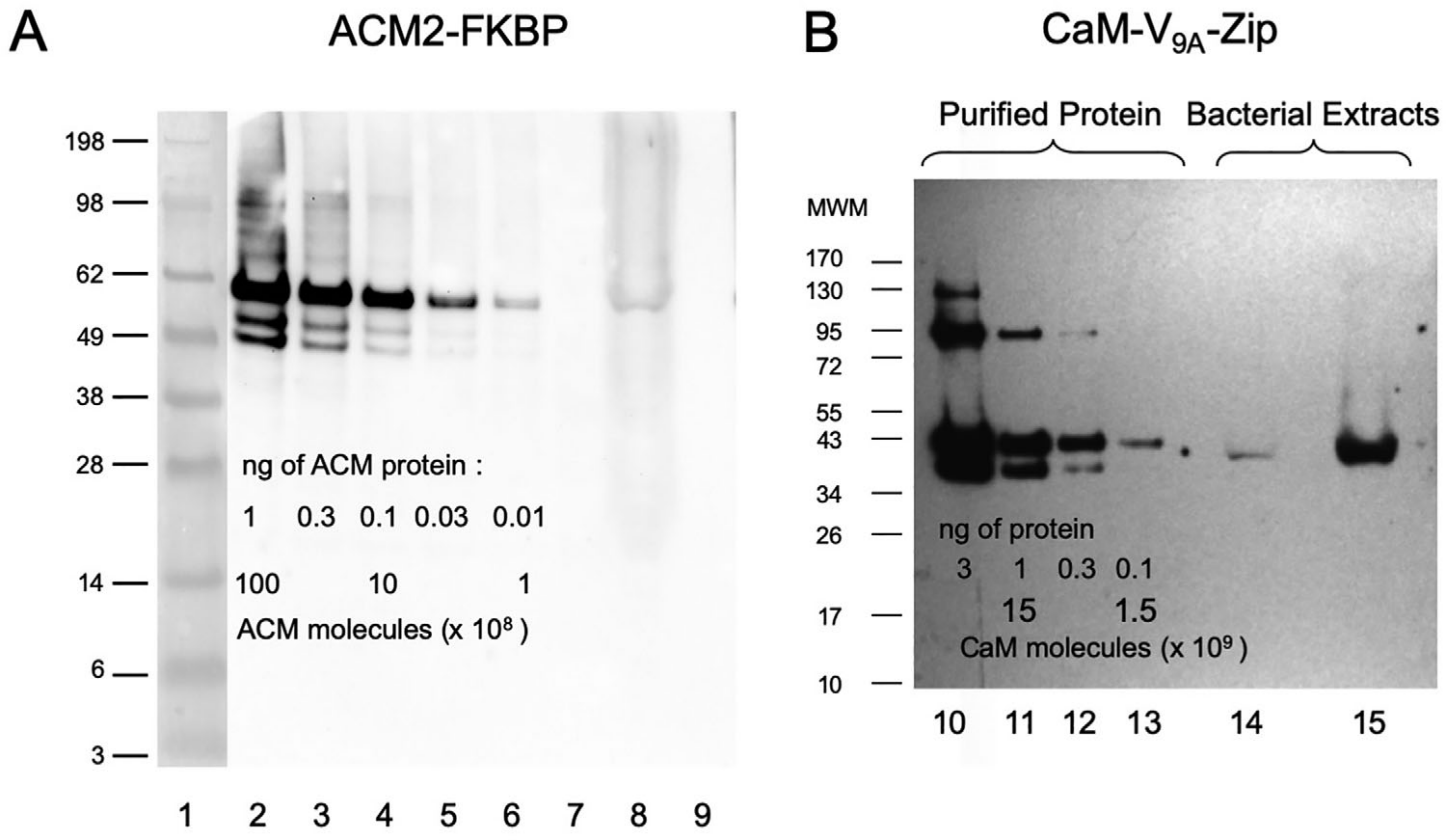
Expression levels of ACM and CaM hybrid proteins *in E. coli*. (A) Western blot analysis of the expression of the ACM2‐FKBP hybrid protein in DHM1. Line 1 : colorimetric image of the prestained molecular weight markers (MWM, size in kDa indicated on the left) run together with the various protein samples (in line 7). Lines 2 to 9 : chemiluminescent image of the membrane to reveal the ACM proteins in the different samples. Lines 2 to 6: 1, 0.3, 0.1, 0.03 and 0.01 ng respectively of the purified ACM2‐FKBP hybrid protein (molecular weight of ≈ 56.5 kDa; 0.01 ng of ACM2‐FKBP fusion correspond to ≈ 10^8^ protein molecules); Line 7: SeeBlue Plus2 prestained Molecular Weight Markers (ThermoFisher Scientific # LC5925); Line 8: total bacterial extract corresponding 1 mL of cell culture at OD_600_ = 1 (i.e. ≈ 10^9^ bacteria) from a mid‐log phase culture of DHM1/pACM2‐Fkbp/pK1Cam‐Frb (grown in the presence of rapamycin). Line 9: no protein. The protein samples were separated by SDS‐PAGE, electro‐transferred to a pvdf western blotting membrane, probed with the 3D1 monoclonal antibody and detected by chemiluminescence imaging (see Experimental Procedures). A colorimetric image of the membrane was then recorded and the line corresponding to the prestained MWM was copied and pasted on the left of the chemiluminescent image. B) Western blot analysis of the expression of the CaM fusion proteins in DHM1. Lines 10 to 13: 3, 1, 0.3, and 0.1 ng, respectively of the purified CaM‐V_9A_‐Zip protein (molecular weight of ≈ 43 kDa; 0.1 ng of CaM‐V_9A_‐Zip fusion correspond to ≈ 1.4 × 10^9^ molecules) were separated by electrophoresis, electro‐transferred to pvdf membrane and detected with the 3D1 monoclonal antibody. Lines 14 and 15: Total bacterial extracts (corresponding to 1 OD_600,_ i.e ≈ 10^9^ bacteria) from mid‐log phase cultures of DHM1 cells harboring the indicated plasmids were probed in parallel by Western blot; Line 14: pACM2‐Zip/pK2Cam‐V_9A_‐Zip; Line 15: pACM2‐Zip/pK1Cam‐V_9A_‐Zip. PageRuler (ThermoFisher Scientific # 26616) was used as molecular weight markers (MWM, size in kDa indicated on the left). Similar results were obtained in 4 independent experiments (biological replicates), carried out with distinct bacterial extracts and purified protein preparations.

We next quantified the level of expression of the ACM and CaM hybrid proteins in the bacterial cells by western blot (WB). The ACM proteins could be specifically detected with a monoclonal antibody (Mab) 3D1 that recognizes an epitope located between residues 373 and 400 of AC [41]. To quantify the amount of ACM2‐FKBP protein produced per bacterial cell, the hybrid protein was over‐expressed in *E. coli* (Supporting Information) and purified to near homogeneity (see Figure S3) to serve as a standard. As shown in Figure 6A, the 3D1 Mab was able to detect down to ≈ 0.01 ng of ACM2‐FKBP fusion, which correspond to about 10^8^ ACM2‐FKBP molecules (molecular weight of ≈ 73 kDa). A bacterial extract corresponding to 1 mL of culture at OD_600_ = 1, i.e. about 10^9^ bacteria), of DHM1 cells expressing ACM2‐FKBP and CaM‐FRB (grown in the presence of rapamycin) was probed in parallel on the same WB. As shown in Figure 6A, a very faint signal (in the range of that found for 0.01 ng of ACM2‐FKBP fusion) could be detected by WB in this extract. This indicates that the bacteria harboring pACM2‐FKBP expressed, on average, less than one ACM2‐FKBP molecule per cell (i.e. below 0.1 ng of protein fusion per 10^9^ cells).

We analyzed similarly different extracts of bacterial cultures harboring various combinations of plasmids, e.g. pAC0‐Gfp, pACM1‐Gfp, or other pACM2 plasmids derivatives, (Figure S4A), and in all cases the WB signal intensities (actually the absence of signal) confirm that less than 1 molecule of ACM fusions were expressed per cell with the different pACM1 and pACM2 hybrid proteins. In control experiments (Figure S4B) we checked that the very low level of detection of ACM fusions in the bacterial extracts was not due to the presence of the large amount of bacterial proteins, as sub‐ng amounts of purified ACM2‐GFP spiked in the extract could be readily detected by WB with the 3D1 Mab. All together, these WB experiments indicate that the expression level of AC hybrid achieved with the ESACH vectors is on average below one molecule per bacterial cell.

The expression level of the CaM fusions produced from the pK1Cam and pK2Cam plasmids were similarly determined by WB using the same 3D1 Mab, as the CaM‐V_9A_‐Zip fusion also contains the AC‐derived 3D1 epitope (initial attempts to quantify the CaM‐fusion with a FLAG‐tagged CaM turned out to be not sensitive enough, data not shown). The CaM‐V_9A_‐Zip fusion was overexpressed and purified to serve as standard (see Supporting Information). About 0.1 ng of the purified CaM‐V_9A_‐Zip fusion could be detected by Mab 3D1 in WB (Figure 6B) corresponding to ≈ 14 × 10^8^ protein molecules (molecular weight of ≈ 42 kDa). A similar signal was detected in total extracts of 10^9^ DHM1 cells harboring pK2Cam‐V_9A_‐Zip and pACM2‐Zip indicating a ratio of about 1 ‐ 3 CaM fusions per cell. A higher signal was found in extracts of DHM1 cells harboring pK1Cam‐V_9A_‐Zip and pACM2‐Zip, corresponding to about 5 ‐ 15 CaM hybrids per bacteria (note that given the mean volume of the *E. coli* cytosol, 1 molecule per cell corresponds roughly to a concentration of 1 nm) (Figure 6B).

Altogether, these results highlight the exquisite sensitivity of the AC/CaM/cAMP signaling cascade that could reliably detect in *E. coli*, interactions between hybrid proteins expressed at a minimal level of few molecules per cell in the case of the CaM fusions, and more strikingly, at less than one molecule per cell, on average, in the case of the ACM fusions.

### 2.6 Characterization of Interactions Involving Toxic Proteins

**FIGURE 7:**
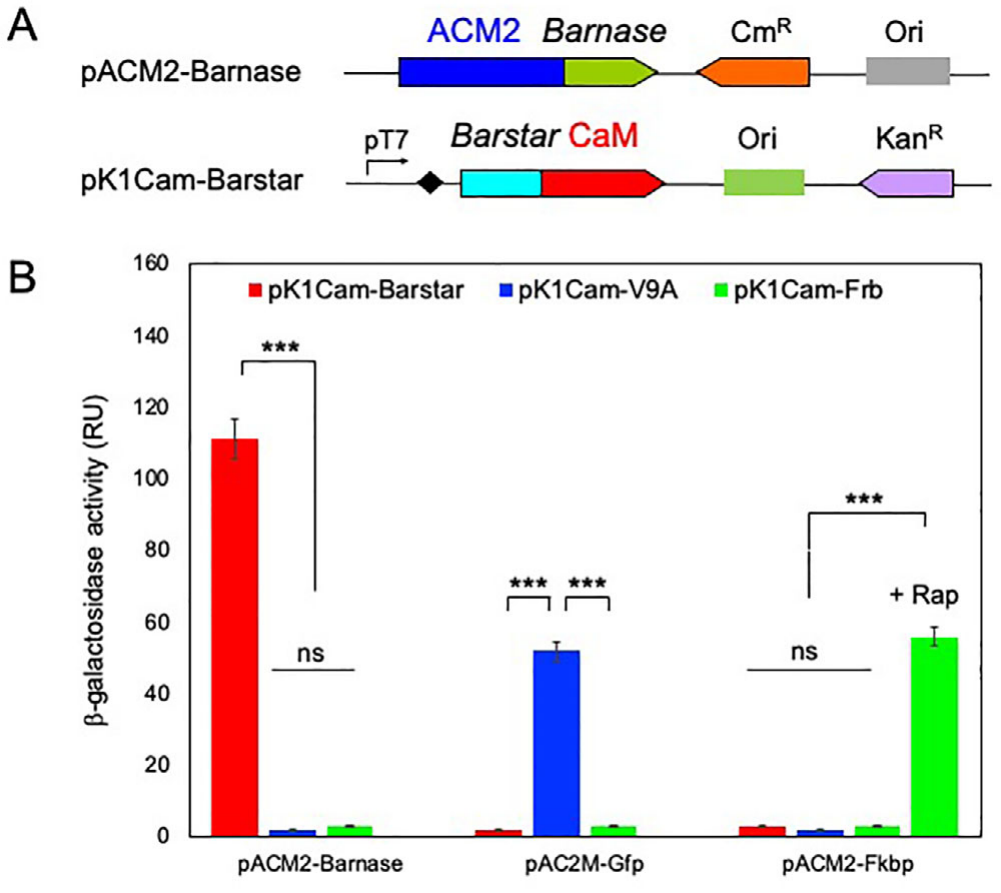
In vivo detection of Barnase/Barstar interaction with ESACH system. (A) Schematic representation of pACM2‐Barnase & pK1Cam‐Barstar plasmids. The colored boxes represent the ORFs of different genes, with the arrow indicating the direction of transcription/translation. (B) β‐galactosidase activities in DHM1 cells transformed with the indicated plasmids and grown at 30°C in LB medium supplemented with IPTG and antibiotics, and 5 µM rapamycin when indicated (+ Rap). For each combination, bars represent mean ± SD of β‐galactosidase activities, measured on 6‐8 independent colonies (technical replicates). Statistical differences were established by one‐way ANOVA followed by Tukey's multiple comparison test. ***p < 0.001, n.s., non‐significant. Similar results were obtained in 3 independent experiments (biological replicates).

To further establish that the ACM fusions are expressed at an extremely low level in bacteria, we explored the interaction of the toxic enzyme barnase, a ribonuclease secreted by the bacterium *Bacillus amyloliquefaciens*, with barstar a specific inhibitor that binds with high affinity to barnase and blocks its RNAse activity. Barnase is lethal to bacterial cells when expressed without its inhibitor barstar [42, 43]. Synthetic barnase and barstar genes were cloned into the pACM2 and pK1Cam plasmids, respectively. DHM1 cells cotransformed with the two resulting plasmids, pACM2‐Barnase and pK1Cam‐Barstar, exhibited a strong Cya*^+^* phenotype, while control co‐transformations with various pACM2 and pK1Cam derivatives demonstrated the selectivity of interaction between the barnase and barstar modules (Figure 7). Noticeably, the pACM2‐Barnase plasmid could be transformed into DHM1 cells expressing no barstar fusion, and the transformed cells did not exhibit any detectable growth problem (as indicated by growth kinetics, see Figure S5). This confirms that the ACM2‐Barnase hybrid protein was expressed at a level low enough not to affect the bacterial physiology, yet sufficient to allow an efficient detection of the barnase‐barstar interaction. Hence the ESACH system may be used to characterize the interaction properties of toxic proteins in bacteria, including the wide variety of toxin‐antitoxin systems that are present in these organisms [44].

### 2.7 Screening of Nanobody‐Antigen Interactions in Bacteria

We showed above that the ESACH system can detect of the specific association of the two different nanobodies V_9A_ and V_1K_ with their target antigen GFP [32]. To examine whether it could also be applied for direct in vivo selection of nanobodies (or other binding modules [45, 46], we randomly PCR‐mutagenized the V_9A_ nanobody at amino acid residue 107, located at the interface with GFP in the crystal structure [32], and screened for variants unable to bind to GFP (see Figure S6). One such variant, Y_107N_, harboring a change of Tyr107 into Asn, abolished the interaction with GFP as revealed by the Cya^−^ phenotype of DHM1/pACM2‐Gfp/pK2Cam‐Y_107N_ transformants. The plasmid pK2Cam‐Y_107N_ expressing the V_9A_‐Y_107N_ variant was chosen for a second round of mutagenesis in which the three amino acid positions 105, 106 or 107 of the V_9A_ were randomly mutagenized (Figure S6). The mutagenized plasmid pool was co‐transformed into DHM1/pACM2‐Gfp and plated on a selective medium [47, 48], a minimal medium containing maltose as a unique carbon source: as the maltose regulon is under a stringent cAMP/CAP control, only Cya*^+^* bacteria can grow on this medium (Xgal and IPTG were also added to better visualize the Cya*^+^* colonies). We randomly chose a number of Cya*^+^* colonies that grew on this selective medium and sequenced their pK2Cam‐V_9A_ plasmids. As shown in Table S3, all fusions contained a tyrosine (or in one case a phenylalanine) at position 107, and a glycine residue at position 106, indicating that these residues were mostly critical for interaction of V_9A_ with GFP. In contrast, a variety of residues could be found in Cya^+^ clones at codon 105, indicating that this position was less important for interaction, in agreement with the 3D structure of the GFP/V_9A_ complex [32]. As a control, the mutagenized plasmid pool was also co‐transformed into DHM1/pACM2‐Fkbp and plated on the same selective medium. No colonies could be detected in these conditions highlighting the stringency of the selection. All together, these experiments indicate that the ESACH system could be used for direct in vivo selection of binding proteins.

### 2.8 Visualization of Active Hybrid AC/CaM Complexes in Living Bacteria

**FIGURE 8:**
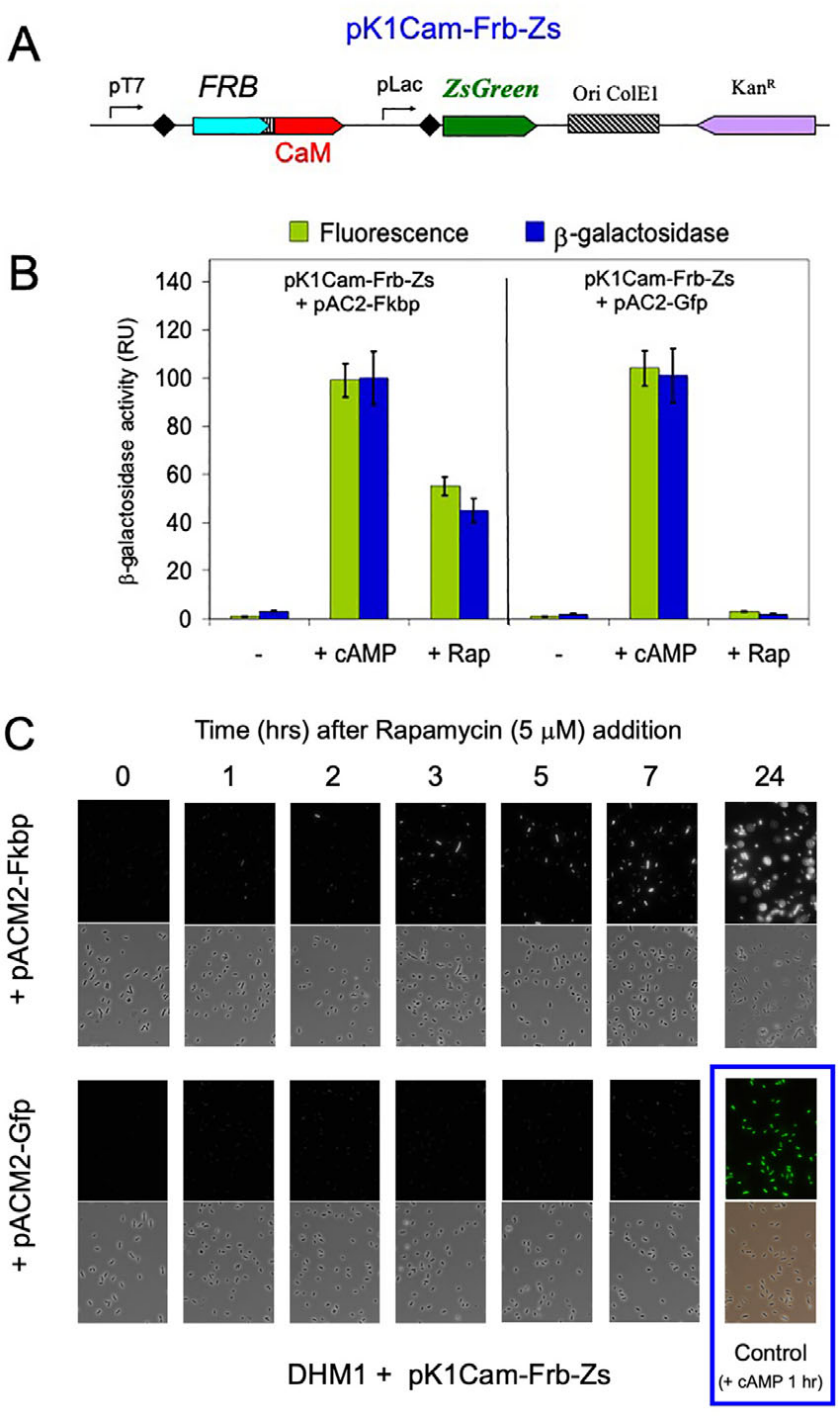
In vivo detection of rapamycin‐induced ACM2‐FKBP/CaM‐FRB interaction. (A) Schematic representation of pK1Cam‐Frb‐Zs plasmid. The colored boxes represent the ORFs of different genes, with the arrow indicating the direction of transcription/translation. pLac correspond to the lac promoter driving the expression of the ZsGreen fluorescent protein. (B) DHM1 cells, harboring either pK1Cam‐Frb‐Zs and pACM2‐Fkbp (left) or pK1Cam‐Frb‐Zs and pACM2‐Gfp (right), were grown overnight at 30°C in LB medium containing appropriate antibiotics, IPTG (100 µM), and when indicated, either 2 mM cAMP or 5 µM Rapamycin (Rap). ZsGreen fluorescence (green bars) and β‐galactosidase activities (blue bars) were measured (on 6 to 8 distinct colonies for each condition, technical replicates) as described in Material and Methods. The bars represent mean ± SD. Statistical differences were established by one‐way ANOVA followed by Tukey's multiple comparison test. For DHM1/pK1Cam‐Frb‐Zs/ pACM2‐Fkbp (left), *p* values for no addition vs cAMP addition, for no addition vs Rapamycin addition, or cAMP addition vs Rapamycin addition were all < 0.001. For DHM1/pK1Cam‐Frb‐Zs/ pACM2‐Gfp (right), *p* values for no addition vs cAMP addition, or cAMP addition vs Rapamycin addition were < 0.001, while no addition vs Rapamycin addition were non‐significant. (C) DHM1 cells, harboring pK1Cam‐Frb‐Zs and either pACM2‐Fkbp or pACM2‐Gfp, were grown overnight at 30°C in LB medium containing appropriate antibiotics then diluted 1:100 in LB medium containing antibiotics and IPTG (100 µM) and further incubated at 30°C until early exponential phase. Rapamycin (5 µM) was added at time 0 and cells were imaged at the indicated time on a Nikon epi‐fluorescence microscope. Bottom right: control experiment in which DHM1/pK1Cam‐Frb‐Zs/pACM2‐Gfp cells were imaged 1 hr after addition of 2 mM cAMP instead of rapamycin. Similar results were obtained in 4 independent experiments (biological replicates).

**FIGURE 9:**
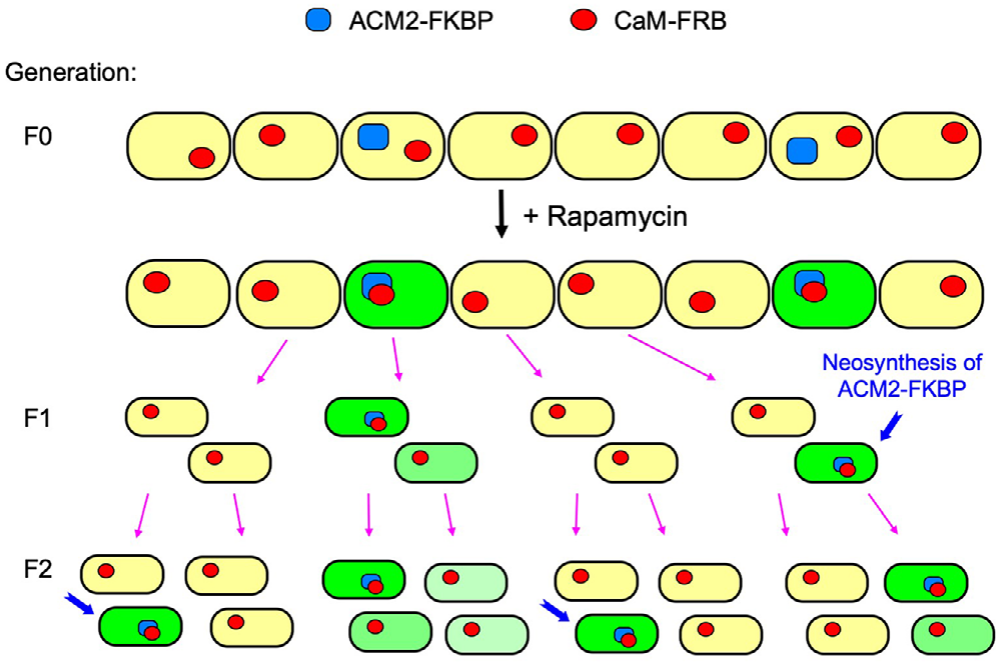
Model of phenotypic inheritance of Cya^+^ phenotype in ESACH system. In bacterial cells (yellow) harboring pACM2‐Fkbp and pK1CaM‐Frb, the ACM2‐FKBP hybrid protein (blue) is expressed on average in 1 out 4 bacterial cells, while the CaM‐FRB fusion (red) is expressed in all cells. Upon addition of rapamycin, cells harboring a ACM2‐FKBP hybrid protein are becoming fluorescent as a result of cAMP‐induced ZsGreen expression (green) while other remains non‐fluorescent (yellow). Over time, the progeny of the ACM2‐FKBP harboring cells will gradually loss the fluorescence (shaded green) due to progressive dilution of the ZsGreen protein, until new events of neo‐synthesis of ACM2‐FKBP (blue arrows) resume the full expression of ZsGreen.

To further document the counterintuitive observation that cells could express on average less than one ACM/CaM active complex per bacterium and yet display a selectable Cya*^+^* phenotype, we attempted to visualize the complementation between hybrid proteins in vivo, on individual bacteria through a fluorescent reporter. For this, the gene coding for the ZsGreen fluorescent protein (Clontech Laboratories) was placed under the transcriptional control of a cAMP/CAP dependent *lac* promoter and inserted into the pK1Cam‐Frb plasmid (Figure 8A). The resulting plasmid pK1Cam‐Frb‐Zs was co‐transformed into DHM1 cells with either pACM2‐Fkbp or pACM2‐Gfp. The co‐transformants displayed a background fluorescence signal when grown in LB medium but were highly fluorescent when grown in LB medium supplemented with cAMP, which can diffuse inside the cells to directly stimulate cAMP‐dependent gene transcription (Figure 8B). As expected, DHM1 cells co‐transformed with pK1Cam‐Frb‐Zs and pACM2‐Fkbp also displayed high fluorescence when grown in LB medium supplemented with rapamycin while those co‐transformed with pK1Cam‐Frb‐Zs and pACM2‐Gfp remained non‐fluorescent (Figure 8B). This indicates that the placZsgreen fluorescent reporter could reveal the rapamycin‐induced interaction between ACM2‐FKBP and FRB‐CaM and the resulting activation of AC enzymatic activity. We then imaged the kinetics of rapamycin‐induced activation of ACM2‐FKBP by CaM‐FRB in cells grown in LB medium. As shown in Figure 8C, within the first hours of growth in the presence of rapamycin, only about 20‐25 % of the cells became fluorescent. This fraction progressively increased to more than 90 % of total population after an overnight culture. No fluorescent cells were detected when rapamycin was added to DHM1/pACM2‐Gfp‐GFP/pK1Cam‐Frb‐Zs. Yet, all these cells became highly fluorescent within 0.5–1 h after addition of cAMP in the medium (“control” in Figure 8C). We interpret these results as follows, as illustrated in Figure 9: in DHM1 cells harboring pACM2‐FKBP, the ACM2‐FKBP fusion protein is stochastically expressed on average in about 20‐25 % of the cells. In these cells, addition of rapamycin triggers the interaction of the hybrid enzyme with the co‐expressed FRB‐CaM, and these bacteria start to produce cAMP and consequently to express the ZsGreen fluorescent reporter. The other cells, lacking the ACM2‐FKBP hybrid, obviously cannot produce cAMP and thus remain non‐fluorescent. However, upon prolonged exposure to rapamycin, the progeny of these ZsGreen^−^ cells will progressively became fluorescent as a result of stochastic expression of the ACM2‐FKBP that should occur statistically once every 2‐3 cell cycles (if present statistically in 20%–25% of the cells). The progeny of the ZsGreen^+^ cells should retain the fluorescence of the mother cell due to the random distribution of the ZsGreen proteins between the two daughter cells, as well as that of the cAMP/CAP complex, which could thus trigger transient de novo ZsGreen expression in daughter cells. In addition, one of the two daughter cells should inherit the active ACM/CaM complex to continue synthesizing high amounts of cAMP. All together, these data support the view that the exquisite sensitivity of the ESACH system could allow bacterial colonies to be selected for their Cya^+^ phenotype, even though at any given time only a fraction of all bacteria may harbor an active ACM/CaM hybrid complex.

## 3 Discussion

We describe here the design of an exquisitely sensitive genetic assay, ESACH, able to report protein interactions at minimal expression levels, close to or below single molecule per cell. Taking advantage of the high enzymatic activity of the *B. pertussis* adenylate cyclase (AC) when activated by its natural activator, the eukaryotic protein calmodulin (CaM), we demonstrate that AC can confer a selectable trait to a bacterial host even when it is expressed, on average, at less than one molecule per cell. This remarkable property originates from the particular characteristics of the cAMP signaling cascade, combined with the high turnover number of CaM‐activated AC (k_cat_ > 1 000 s^−1^). Indeed, in a given bacterial cell, a single molecule of active AC/CaM complex can rapidly synthesize enough cAMP to saturate the CAP proteins and activate the transcription of cAMP/CAP‐dependent genes. When this cell divides, the daughter cell that does not inherit the single AC/CAM complex will nevertheless inherit the over‐expressed metabolic enzymes (e. g., lactose or maltose‐metabolizing ones) as well as the cAMP/CAP molecules produced in the mother cell (Figure 9). Therefore, this daughter cell is able to grow for a while on the selection medium without actually harboring any active AC enzyme. As this daughter cell further divides, the concentrations of cAMP/CAP and cAMP‐dependent catabolic enzymes will progressively decrease until they reach a level insufficient to sustain growth on the selective medium. These cells will resume their growth when a new stochastic event of AC expression restarts a new cycle. Although we did not precisely determine the frequency of the stochastic expression of the AC enzyme with our system, the fact that about 20%–25% of the cell population expressed a Cya*^+^* phenotype when rapamycin was added to promote the interaction and subsequent activation of ACM2‐FKBP by CaM‐FRB, suggests that an ACM2‐FKBP molecule is produced on average every two division cycles. In our experimental settings, CaM‐FRB fusion was produced at an average level of about 3–10 molecules per cell so that enough CaM‐FRB should be present in each cell to activate any neo‐synthesized ACM2‐FKBP.

The combined properties of high AC activity and of the cAMP/CAP signaling cascade in *E. coli* thus offers a unique approach to characterize protein interactions at minimal levels, and possibly at single‐molecule‐per‐cell level. Single molecule experimentation has allowed exploring biological mechanisms that cannot be easily disclosed by ensemble‐level experiments [49, 50, 51, 52]. Most of these studies have been performed through optical imaging of GFP tagged components expressed at the minimal observable level. The ESACH system provides the extreme sensitivity required for functional characterization of biomolecular processes in bacteria, at extremely low level of expression, potentially close to or below a single molecule per cell.

Interestingly, it has been reported that a significant fraction of all proteins (about half of the 1000 tested) in an *E. coli* cell might be present at low levels, that is, below ten molecules per cell [53]. Although most of these low‐abundance proteins may be non‐essential, a significant fraction of them were found to be implicated in genetic interactions as indicated by the patterns of synthetic sickness and/or synthetic lethality observed in experiments of double‐knockout combinations [54]. Characterization of the interaction networks in vivo of these low‐abundance proteins at their native expression level may be instrumental to further explore their physiological function. The fact that numerous proteins may be expressed only at very low copy number in *E. coli* and possibly in other bacterial species, is calling for the development of technologies allowing such single‐molecule experiments in living organisms. Currently only high‐resolution microscopy techniques are able to detect a single molecule in live cells [51, 52, 55]. The ESACH system could provide a simple and efficient approach to study protein interactions, as well as protein modifications or degradations, at such extremely low levels of expression.

Further work will be required to properly evaluate the ESACH assay for large‐scale screening of protein interaction networks and compare its capabilities to other previously designed bacterial two‐hybrid assays (including the BACTH technique). Due to the low expression level of the hybrid proteins, the ESACH system will likely be selective for high affinity interactions compared to other two‐hybrid systems in which the fusion proteins are expressed at much higher levels (e.g. hundreds of molecules per cell). It will be interesting to precisely determine the range of affinities that can be detected by the ESACH assay compared to that of other protein‐complementation systems.

The high sensitivity of the ESACH assay could be particularly adapted for direct in vivo screening of high affinity antibodies (e.g. single chain antibody fragment, scFv, or nanobody [45]) or other “binders” based on different scaffolds [46], to specific antigens or proteins of interest. We anticipate that ESACH could be efficiently combined with deep learning techniques to facilitate identification and evolution of recombinant antibodies or in silico‐designed biomolecules with high affinity and selectivity [56, 57, 58, 59, 60, 61].

In summary, we have shown here that the high catalytic activity of the CaM‐activated AC can be combined with the endogenous cAMP signaling cascade of *E. coli* to design a novel experimental tool for exploring biological processes at extremely low protein expression level and potentially at the single molecule level in living cells. This may open novel avenues for studying in bacteria the molecular basis of biological mechanisms that haven't been accessible up to now with current methodologies. In addition, we can also speculate that the ESACH technique might be exploited to design synthetic regulatory networks or biological logic‐gates [62] operating at, or even below, one molecule per cell. It could also be an attractive experimental tool to explore the molecular basis of phenotypic heterogeneity arising from random segregation of a small number of proteins or complexes between daughter cells during cell division [63, 64, 65, 66, 67]. These stochastic events are keys for cell‐to‐cell variability leading to bacterial individuality in microbial populations and are likely an important mechanism for adaptation and evolution [68].

### 3.1 Limitations of the Study

Although this study indicates that the ESACH signaling cascade can detect less than one interacting hybrid complex per cell, on average on the whole cell population, it does not directly demonstrate single‐molecule interaction detection as we did not perform precise molecular counting at single cell level. In particular, we cannot exclude that among the population of Cya^+^ bacteria, some cells may produce two or more molecules of ACM hybrids. It is possible that when a cell transcribes a mRNA encoding the ACM hybrid—likely also as a single mRNA molecule—several proteins could be translated from that mRNA. Characterization of the mRNA transcription and translation of ACM hybrids at the extremely low level of expression used in the described ESACH assay remains to be carried out.

## 4 Experimental Procedures

### 4.1 General Methods

Bacteria were routinely grown at 30°C in LB broth (0.5% yeast extract, 1% tryptone) containing 0.5% NaCl [69]. Unless stated otherwise, antibiotics were added at the following concentrations: ampicillin (100 µg/mL), chloramphenicol (30 µg/mL), kanamycin (50 µg/mL). Standard protocols for molecular cloning, PCR, DNA analysis, transformation and P1 transduction were used [69]. The *E. coli* strain XL1‐Blue (Stratagene / Agilent Technologies) was used for all routine cloning experiments. PCR primer's synthesis and DNA sequencing were carried out by the company Eurofins MWG Operon (Ebersberg, Germany). Synthetic genes coding for the V_9A_ and V_1K_ V_H_H, barnase, and barstar were obtained from Geneart (Thermo Fisher Scientific). Synthetic genes coding for the FtsI and FtsW proteins from *Pseudomonas aeruginosa and* PulG proteins from *Klebsiella oxytoca* were obtained from Twist Bioscience (South San Francisco, CA, USA).

### 4.2 Plasmid Constructions

Plasmids coding for AC wild‐type, ACM247, ACM335, and CaM were described in Ladant et al. [30] and Vougier et al. [33]. The plasmids pAC0, pACM1, and pACM2 are derived from the pT25 plasmid harboring a p15A origin of replication and a chloramphenicol resistant marker [24]. They were constructed by subcloning the full‐length AC (or ACM1 = ACM247; ACM2 = ACM335) genes and removal of the transcriptional and translational sequences in front of AC or ACM coding regions. The maps and DNA sequences (full‐length sequence for pAC0 and, for other plasmids only the specific *Cla*I‐*Xho*I DNA fragments of the other plasmids) of these plasmids are shown in Appendix 1.

Genes coding for GFP (from plasmid pDSW207‐blr [70], FKBP (kindly provided by Dr Yves Jacob, Institut Pasteur), Barnase (synthetic gene, ordered from Geneart, Thermo Fisher Scientific) GCN4 leucine zipper (from plasmid pUT18C‐zip [71]), and the first transmembrane segment of OppB followed by the GCN4 leucine zipper (from plasmid pUTM18C‐zip [27]) were subcloned in the C‐terminal multicloning site. Plasmid pACM2‐TM‐zip was constructed by subcloning between the *BamH*I and *Xho*I sites of pACM2‐Gfp, a PCR‐amplified DNA fragment (with appropriate primers introducing a *Bgl*II site—compatible with *BamH*I—and a *Xho*I site) that codes for the linker region, the *E. coli* OppB first TM segment and the GAL4 leucine zipper (zip) domain from plasmid pUT18C‐TM‐zip [27].

Plasmids pTrACM1‐Fkbp, pTrACM2‐Fkbp, and pTrACM2‐Gfp are derivatives of plasmid pTRAC384GK [33], in which the AC coding region is under the control of the phage λ Pr promoter, that is repressed at temperatures below 32°C by the thermosensitive λ repressor cI^857^ (Figure 2 and Appendix 1). They were constructed by subcloning appropriate fragments from plasmid pACM1‐Fkbp, pACM2‐Fkbp, and pACM2‐Gfp into pTRAC384GK.

Plasmid pDLTCaM41^33^ harbors a ColE1 origin of replication, an ampicillin resistant marker, and a wild‐type CaM gene under the control of the phage λ Pr promoter that is repressed at temperature below 32°C by the thermosensitive λ repressor cI^857^ (Figure 2). Plasmid pTCam is a derivative of pDLTCaM41, expressing, under control of the thermoinducible λ promoter, CaM fused to a 6‐histidine tag at its N‐ter and a tetra‐cysteine tag at its C‐terminus (Appendix 1). Plasmid pTCam‐V_9A_ is a derivative of pTCam expressing CaM fused to the V_9A_ nanobody at its N‐ter and a tetra‐cysteine tag at C‐terminus (Figure 2 and Appendix 1).

Plasmids pK1Cam and pK2Cam are derivatives of the pMK‐RQ vector (Geneart, Thermo Fisher Scientific; contains a ColE1 origin of replication and a Kanamycin resistant marker) and harbor a synthetic CaM gene (Geneart) with a N‐terminal multicloning site and an AviTag sequence (allowing for enzymatic biotinylation in vivo) appended to its C‐terminus. The CaM gene is placed under the control of a T7 promoter with (pK1Cam) or without (pK2Cam) a ribosome binding site (Figure 2; Table S1, and Appendix 1). Plasmid pDL1312, used here as a control plasmid, is the parental vector of pDLTCaM41 expressing neurocalcin instead of CaM [72]. Genes coding for the camelidae V_H_H 3G9A (here called V_9A_) and V_H_H 3K1K, (here called V_1K_) [32], FRB, and barstar were subcloned in frame into the N‐terminal multicloning site. Plasmids pK1Cam‐V_9A_‐Zip and pK2Cam‐V_9A_‐Zip (Figure 5A; Table S1, and Appendix 1).) were obtained by cloning a 274 bp *BstB*I‐*Xho*I fragment from pACM2‐Zip (encoding AC residues 373‐400 followed by the GCN4 leucine zipper) between the *Cla*I and *Xho*I sites of the pK1Cam‐V_9A_ or pK2Cam‐V_9A_ vectors.

Plasmids pK1Cam‐V_9A_‐TM‐Zip (Figure 5A; Table S1, and Appendix 1) was obtained by cloning between the into *Xho*I & *BamH*I sites of pK1Cam‐V_9A_ a PCR‐amplified fragment from pACM2‐TM‐Zip, encoding the TM(OppB)‐Zip segment.

Plasmid pK1Cam‐Frb‐Zs was obtained by inserting, between the *Spe*I and *BamH*I sites of pK1Cam‐Frb plasmid, a PCR fragment encoding the ZsGreen fluorescent protein gene (from plasmid pZsGreen from Clontech, Takara) placed under a pLac promoter (see Figure 8A; Table S1, and Appendix 1).

Plasmid pCaM_VU8_ was synthesized by Geneart and contains a synthetic gene encoding the CaM‐VU8 variant [35] fused to a HA tag and a multicloning site, cloned into the pMA‐T vector (Geneart). Plasmid pCaM_VU8_‐V_1K,_ was obtained by inserting a PCR fragment encoding the V_1K_ gene between the *Mlu*I and *Sac*I sites of pCaM_VU8_. Plasmid pCaM_Cter_‐V_1K_ was obtained by inserting a PCR fragment encoding the C‐terminal moiety of CaM [36] (residues 77 to 149) between the *Nhe*I and *Spe*I sites of pCaM_VU8_‐V_1K_.

The FtsL and FtsQ genes were amplified from *E. coli* DNA and cloned (using the Gibson technique) into the *BamH*I site of pACM2‐Zip or the *Xba*I site of pK1Cam‐V_9A_‐Zip. Synthetic genes encoding FtsI and FtsW from *Pseudomonas aeruginosa* and PulG of *Klebsiella oxytoca* were obtained from Twist Bioscience and cloned similarly (with the Gibson technique) into the *BamH*I site of pACM2‐Zip or the *Xba*I site of pK1Cam‐V_9A_‐Zip.

The sequences of all recombinant plasmids were verified by DNA sequencing (Eurofins‐MWG, Les Ullis, France).

### 4.3 ESACH Complementation and Screening Assays

ESACH complementation assays were carried out in the *E. coli Δcya* strain DHM1 [26]. After transformation with appropriate plasmids, cells were plated on LB agar containing X‐Gal, IPTG plus antibiotics and incubated at 30°C for 24–36 h. Efficiency of interaction between hybrid proteins was quantified by measuring β‐galactosidase (β‐Gal) activity in liquid cultures in 96‐well format assay [70]. For each set of transformation, the β‐Gal assays were performed on 6 to 8 overnight cultures each inoculated with distinct colony and grown at 30°C in 300 µL LB broth in the presence of 0.5 mM IPTG and appropriate antibiotics in a 96‐well microtiter plate (2.2 mL 96‐well storage plate, Thermo Fisher Scientific). For screening experiments, the DHM1 cells, after electroporation with appropriate plasmids, were incubated in LB broth at 30°C for 90 min, then washed several times with M63 synthetic medium [69] and spread on M63 solid medium supplemented with maltose (0.2%), 5‐bromo‐4‐chloro‐3‐indolyl‐b‐D‐galactoside (X‐Gal, 40 µg/mL), isopropyl‐β‐D‐thiogalactopyranoside (IPTG, 0.5 mM), kanamycin (25 µg/mL) and chloramphenicol (20 µg/mL). Plates were incubated at 30°C for 6–10 days until appearance of blue, Cya^+^ (Mal^+^ and Lac^+^) colonies. Cyclic AMP was measured on boiled liquid culture with an ELISA assay as previously described [24, 73].

### 4.4 Purification of ACM and CaM Fusion Proteins

The ACM1‐FKBP, ACM2‐FKBP, and ACM2‐GFP open reading frames were sub‐cloned into plasmid pTRAC384GK [33] and the recombinant plasmids (pTrACM1‐FKBP, pTrACM2‐FKBP, and pTrACM2‐GFP, respectively) were transformed into the *E. coli* BLR strain (Novagen, Darmstadt, Germany). The transformants were grown at 30°C in LB medium containing 100 mg/L ampicillin, and when the culture reached an optical density of 0.6 – 0.8 at 600 nm, expression of the proteins was triggered by shifting the growth temperature to 42°C. After 150 min of additional growth at 42°C, the cells were collected by centrifugation (20 min, 10 000 x *g*, 4°C), and the cell pellets were frozen at −20°C. The cell pellets were resuspended in 20 mM HEPES‐Na, pH 7.5, and disrupted by sonication at 4°C. The sonicated suspension was centrifuged for 20 min at 13 000 x *g* at 4°C. The supernatant was discarded, and the pellet (containing inclusion bodies) was resuspended in 8 M urea with 20 mM HEPES‐Na (pH 7.5) and agitated overnight at 4°C. After 20 min of centrifugation at 13 000 x *g* at 4°C, the supernatant (“urea extract”) that contained the solubilized ACM fusion proteins was collected. The ACM fusions were purified by two sequential chromatographic treatments with DEAE‐Sepharose as previously described [33]. Briefly, the urea extract was first loaded onto a DEAE‐Sepharose column (20 mL of packed resin) equilibrated in 8 M urea with 20 mM HEPES‐Na (pH 7.5). In these conditions, the ACM proteins did not bind to the resin, and were recovered in the flow‐through fractions. The collected flow‐through fractions were then diluted 5 times with 20 mM HEPES‐Na (pH 7.5) and applied to a second DEAE‐ Sepharose column (20 mL of packed resin), which had been equilibrated in 20 mM HEPES‐Na (pH 7.5). In these conditions, the ACM proteins were retained on the resin and, after extensive washing with 20 mM HEPES‐Na (pH 7.5), the proteins were eluted in a soluble form using 20 mm HEPES‐Na (pH 7.5) containing 200 mM NaCl. The AC protein preparations were analyzed by SDS‐PAGE analysis (see Figure S3). The concentrations of ACM fusion proteins were determined from the absorption spectra using an extinction coefficient calculated from their amino acid content.

The FRB‐CaM and CaM‐V_9A_‐Zip protein were expressed in *E. coli* after subcloning the corresponding gene into the pTCam plasmid derivative and purified as described in Vougier et al. [33]. Protein purity was monitored by SDS‐PAGE analysis, and the protein concentrations were determined by absorption at 280 using molecular extinction coefficients calculated from their amino acid sequence.

### 4.5 In Vitro Assays of the Rapamycin‐Induced Activation of ACm‐FKBP by CaM‐FRB

AC assays were carried out as described by Davi et al. [37], in a final volume of 100 µL of 50 mm Tris‐HCl, pH 8.0, 7.5 mm MgCl_2_, 0.1 mm CaCl_2_, 0.5 mg/mL bovine serum albumin (BSA), containing 0.5 nm of ACM1‐FKBP or ACM2‐FKBP, various concentrations of CaM‐FRB (from 0 to 100 µm) (all proteins were diluted in 10 mM Tris‐HCl, pH 8.0, 0.1% Tween 20) and supplemented with 1 mM EGTA and /or 5 µM rapamycin, as indicated. The reaction mixtures were equilibrated at 30°C for 10 min and the enzymatic reactions were initiated by adding 2 mM ATP and further incubated at 30°C for 20 min. Blank assays containing no enzymes or no CaM‐FRB were carried out in parallel. The cAMP produced was then determined spectrophotometrically as previously described [37].

### 4.6 Quantification of the Expression of the Hybrid Proteins in Living Bacteria

For in vivo quantification of hybrid protein expression, DHM1 cells harboring the appropriate plasmids were grown until mid‐log phase (starting from an over‐night culture) washed with M63 medium, resuspended in SDS Gel loading buffer and heated at 100°C for 5 min. Total bacterial extracts, corresponding to ≈ 10^9^ bacteria (i.e. 1 OD_600_ of bacteria) were loaded on a 10% SDS‐polyacrylamide gel as well as various amounts (ranging from 10 to 0.01 ng) of the purified ACM2‐FKBP (521 aa, molecular weight = 56 443 Da; 0.1 ng of ACM2‐FKBP fusion correspond to ≈ 1 × 10^9^ protein molecules), ACM2‐GFP (664 aa; molecular weight = 72 667 Da; 0.1 ng of ACM2‐GFP fusion correspond to ≈ 8 × 10^8^ protein molecules), or CaM‐V_9A_‐Zip (381 aa; molecular weight = 41 869 Da; 0.1 ng of CaM‐V_9A_‐Zip fusion correspond to ≈ 1.4 × 10^9^ molecules) hybrid proteins. After electrophoresis, the proteins were electro‐transferred onto a polyvinylidene difluoride (pvdf) membrane (Millipore), incubated with the anti‐cyaA monoclonal antibody 3D1 [41] (Santa Cruz Biotechnology; sc‐13582) then with a horseradish peroxidase‐conjugated goat anti‐mouse secondary antibody (Santa Cruz Biotechnology, sc‐2031) and finally detected by enhanced chemiluminescence (ECL‐Plus kit from Amersham Biosciences (Cytiva) or the SuperSignal West Atto Ultimate kit from Thermo Fisher Scientific).

### 4.7 Mutagenesis of the V9A Gene and ESACH Screening of Antigen‐Antibody Interactions

Mutagenesis of the V_9A_ gene on plasmid pK2Cam‐V_9A_ was performed by a mutagenic PCR carried out with an oligonucleotide primer containing a degenerated codon NN(C/G) at position 107 (Figure S6). The mutagenized plasmid pool was transformed into DHM1/pACM2‐Gfp competent bacteria and plated on LB agar supplemented with X‐gal (40 µg/mL), IPTG (0.5 mM), kanamycin (50 µg/mL), chloramphenicol (30 µg/mL) plates and grown for 36 h at 30°C. Several white colonies, encoding non‐interacting V_9A_ variants, were randomly picked for plasmid purification and the corresponding pK2Cam plasmids were sequenced. We selected one of these variants, pK2Cam‐V_9A_‐Y_107N_, in which the Y_107_ codon of V_9A_ is changed into an Asn codon for a second round of mutagenesis. It was performed by a mutagenic PCR carried out with oligonucleotide primers containing degenerated codons NN(C/G) at position 105‐107 (Figure S6). The mutagenized plasmid pool was transformed into DHM1/pACM2‐Gfp competent bacteria and plated on LB/X‐Gal/IPTG/chloramphenicol/kanamycin plates and grown for 36 hrs at 30°C. Both white (lac‐) and blue (lac+) colonies were randomly picked for plasmid purification and the pK2Cam plasmids were sequenced (Eurofins‐MWG, Les Ullis, France). Alternatively, the transformed cells were plated on a selective medium made of M63 minimal medium agar supplemented with maltose (0.2%), kanamycin (25 µg/mL), chloramphenicol (20 µg/mL), IPTG (0.5 mM), and X‐gal (40 µg/mL, to facilitate the detection of Cya+ clones that are Mal+ and also Lac+) and grown for several days at 30°C [47, 48]. Growing colonies(mal+) were randomly picked for plasmid purification and the pK2Cam plasmids were sequenced as above.

### 4.8 Fluorescence Microscopy

For fluorescence microscopy studies, overnight cultures of DHM1 cells harboring appropriate plasmids were diluted 1:100 in LB medium containing IPTG (100 µM), and appropriate antibiotics and incubated until early exponential phase at 30°C. Rapamycin (5 µM) was added to induce association of ACM‐FKBP with FRB‐CaM. Images of living, non‐fixed cells were acquired on a Nikon epi‐fluorescence microscope Eclipse 80i equipped with a 100x Plan‐Apo oil immersion objective and a 100 W mercury lamp. Images were captured with a 5‐megapixel colour CCD DS‐5Mc device camera and processed using Adobe Photoshop software [70].

### 4.9 Statistical Analysis

All experiments were performed at least 3 times, each with different preparations of proteins or transformed cells (= biological replicates). For β‐galactosidase activity measurements, the results are presented as mean ± standard deviation (SD) of 6 to 8 cultures each inoculated with a distinct colony (technical replicates). Statistical significance was determined using one‐way ANOVA followed by Tukey's multiple comparison test. *P*‐values less than 0.001 were considered statistically significant. All statistical analyses and graph plotting were performed using Kaleidagraph (Synergy Software, Reading, PA) or Microsoft Excel software.

## Author Contributions

Conceived and designed the experiments: DL. Performed the experiments: MD. Analyzed the data: MD, DL. Wrote the manuscript: DL. Final editing: MD, DL.

## Conflicts of Interest

Both authors are co‐authors on patent applications EP 3 169 782 B1 and US10760072 B2, which cover the design of AC/CaM‐based highly sensitive regulatory circuit to detect protein–protein interaction in bacteria with single molecule sensitivity.

## Supporting information




**Supporting File**: advs75179‐sup‐0001‐SuppMat.docx.

## Data Availability

The data that support the findings of this study are available in the supplementary material of this article.
