# Supplementary material for: Detection of Protein–Protein Interactions in Escherichia coli With Single Molecule Sensitivity
Source: Adv Sci (Weinh). 2026 Apr 10:e10093. Online ahead of print. doi: 10.1002/advs.202510093 (PMC13334617; doi:10.1002/advs.202510093)
Supplement: Supplementary file 1 — Supporting File: advs75179‐sup‐0001‐SuppMat.docx. [file ADVS-9999-e10093-s001.docx]

**Supporting Information**

**Detection of Protein-Protein Interactions in *Escherichia coli* with Single Molecule Sensitivity**

**Marilyne Davi and Daniel Ladant**

Unité de Biochimie des Interactions Macromoléculaires, CNRS UMR 3528, Institut Pasteur, 28 rue du Dr. Roux, 75724 Paris cedex 15, France.

Tel: 331 45 68 84 00; email: [daniel.ladant@pasteur.fr](mailto:daniel.ladant@pasteur.fr)

**Supplementary Note**

**CaM variants as activators of AC in ESACH interaction assays.**

The ESACH uses wild-type CaM and AC variants, ACM1 and ACM2, with reduced affinity for CaM. We also explored an alternative design using as complementary modules, a wild-type AC enzyme and a modified CaM with decreased affinity for AC. We first tested a previously characterized CaM variant, CaM_VU8,_ in which three glutamic acid residues at position 82, 83, and 84 of CaM are substituted by lysines: CaM_VU8_ displays ≈ 1000-fold lower affinity for wild-type AC compared to native CaM^35^. CaM_VU8_ expressed from the pCam_VU8_ plasmid (see Material & methods, Table S1 and Appendix 1).) was unable to activate *in vivo* the wild-type AC or the AC-GFP fusion (see table below). When CaM_VU8_ was expressed as a fusion with the V_1K_ nanobody, it efficiently activated the AC-GFP fusion but not AC alone. Furthermore, CaM_VU8_-V_1K_ could not activate the ACM2-GFP fusion, likely because of the too-low affinity of the mutant CaM_VU8_ for the ACM2 variant.

We then tested the C-terminal moiety of CaM (i.e. residues 77 to 148), CaM_Cter_, as a potential activating partner of AC in ESACH assay: CaM_Cter_ has been shown to fully activate AC with a ≈ 10 fold lower affinity than native CaM^36^. As shown in Table 3, CaM_Cter_ expressed as a fusion with the V_1K_ nanobody efficiently activated the AC-GFP fusion. Yet it also partly stimulated the AC alone, likely because of its relatively high affinity for the enzyme. More interestingly, the CaM_Cter_-V_1K_ fusion efficiently activated the ACM2-GFP fusion but not ACM2 or the ACM2-FKBP fusion. Therefore, the CaM_Cter_ fragment, which is only 72 amino-acid long, can be used in combination with the ACM2 variant for high sensitive detection of interactions in ESACH assay in living bacteria.

**Supplementary Note Table 1 :**

**Complementation assays between various AC and CaM fusions.**

|  | pCam_VU8_ | pCam_VU8_-V_1K_ | pCam_Cter_-V_1K_ |
| --- | --- | --- | --- |
| pAC0 | 5.7 ± 0.5 | 5.0 ± 0.7 | 29 ± 5 |
| pAC0-Gfp | 6.6 ± 0.6 | 111 ± 10 | 187 ± 13 |
| pACM2 | 5.3 ± 0.5 | 4.8 ± 0.7 | 4.6 ± 0.5 |
| pACM2-Gfp | 6.0 ± 0.8 | 5.3 ± 0.5 | 141 ± 15 |
| pACM2-Fkbp | 6.1 ± 0.7 | 4.9 ± 0.7 | 5.0 ± 0.5 |

DHM1 bacteria were transformed with the indicated plasmids and plated on LB agar supplemented with appropriate antibiotics, IPTG, and X-gal and grown at 30°C for 36 hrs in the presence of 0.5 mM IPTG plus appropriate antibiotics. The β-galactosidase activities (expressed in relative units) were determined on liquid cultures grown overnight at 30 °C in LB plus appropriate antibiotics and IPTG. For each transformant, the values represent the mean ± SD of β-galactosidase activities, measured as described in Material and Methods, on 6-8 independent colonies (technical replicates). Similar results were obtained in 3 independent experiments (biological replicates), each performed on distinctly transformed cells.

**Supplementary Figures**

| **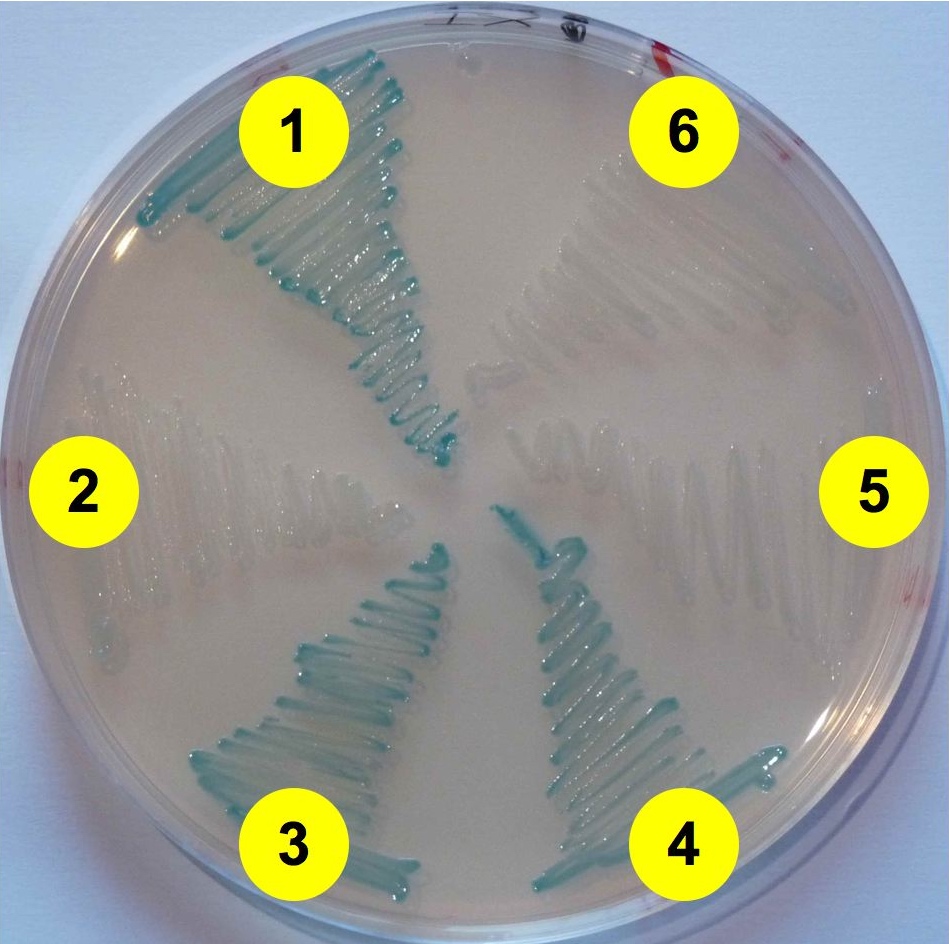** | DHM1 *Δcya* co-transformed with :  1 : pAC0-Gfp + pK1Cam-V_9A_  2 : pACM2-Gfp + pK1Cam-Frb  3 : pACM2-Gfp + pK1Cam-V_9A_  4 : pACM2-Gfp + pK2Cam-V_9A_  5 : pACM2-Fkbp + pK1Cam-V_9A_  6 : pACM2-Fkbp + pK2Cam-V_9A_ |
| --- | --- |
|  |  |

**Figure S1 :** Phenotypic assay of protein interaction on LB–X-gal. DHM1 cells were co-transformed with the indicated plasmids and plated on LB–X-gal agar plates containing 0.5 mM IPTG, chloramphenicol and kanamycin, and incubated for 36 hr at 30°C.


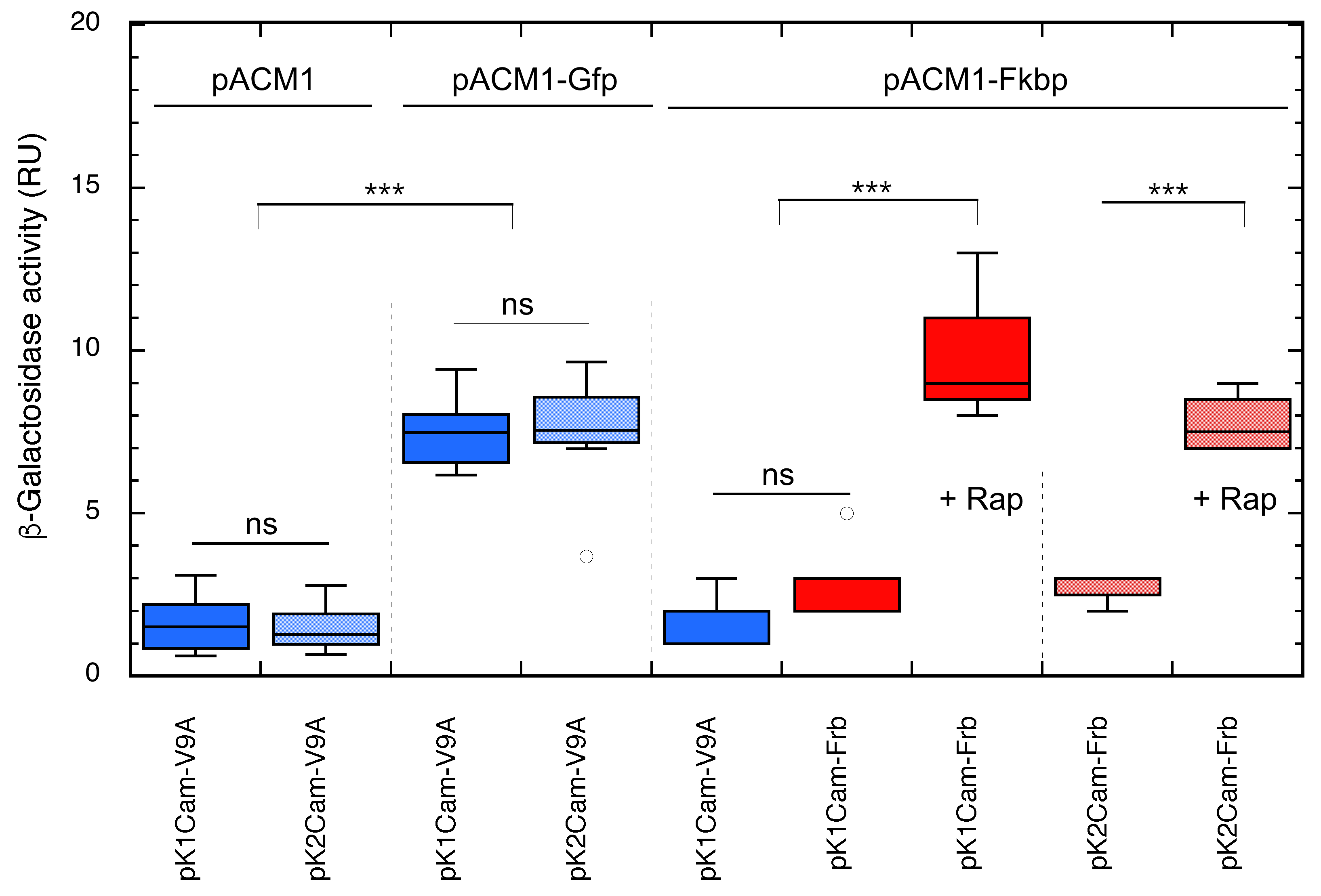


**Figure S2:** β-galactosidase assays of ACM1 complementation at low CaM hybrid expression. DHM1 cells were transformed with the indicated plasmids. For each transformation, 6-8 independent colonies (technical replicates) were picked up and grown overnight at 30 °C in LB medium supplemented with IPTG, antibiotics and when indicated 5 μM rapamycin (+ Rap). For each culture, after cell lysis, as described in Material and Methods, the β-galactosidase assays were carried out for 60 min and represented as boxplot. *** p < 0.001, n.s., non-significant. Similar results were obtained in 3 independent experiments (biological replicates), each performed on distinct transformed cells.

**
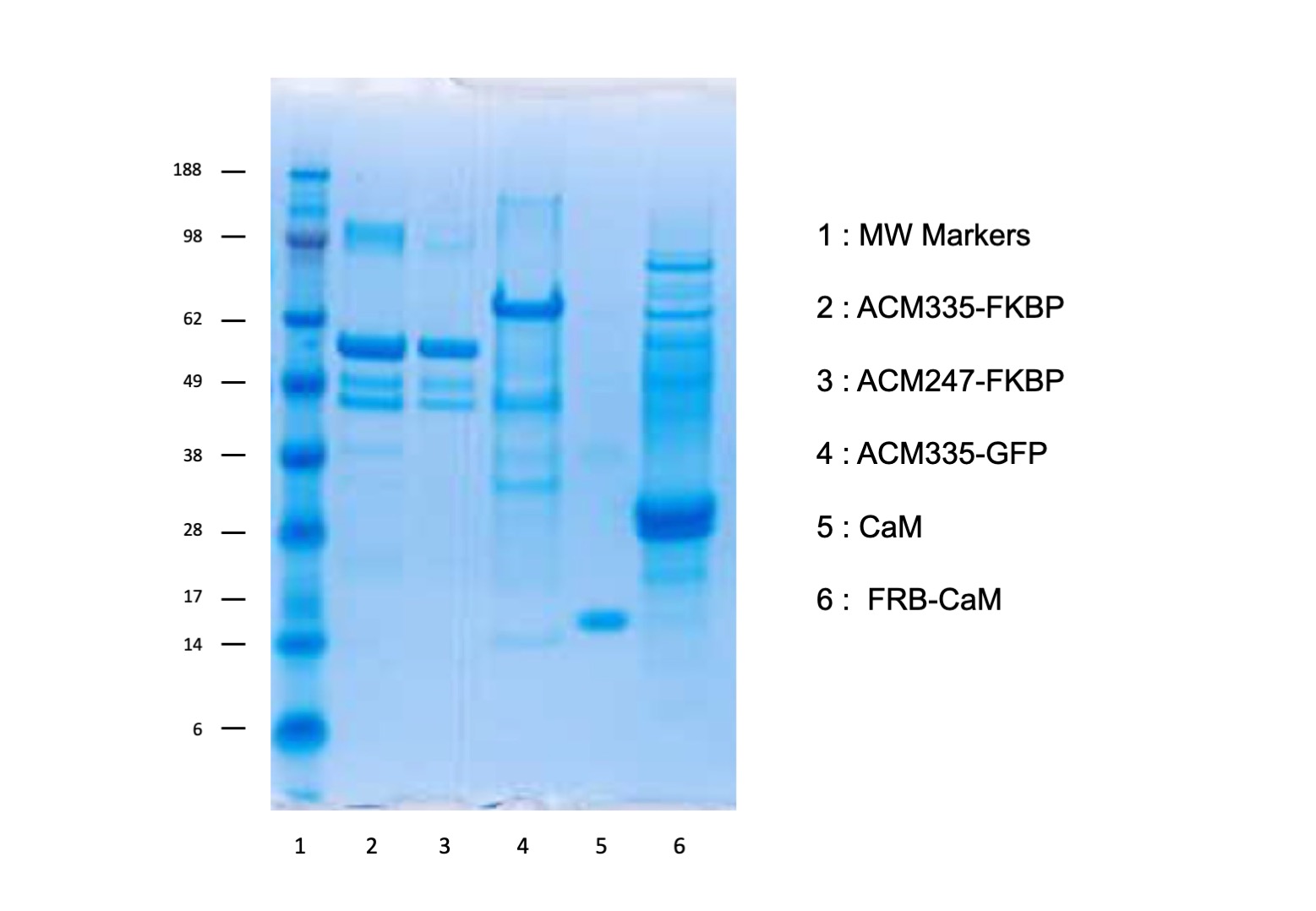
**

**Figure S3:** SDS-PAGE analysis of purified ACM and CaM fusions. The proteins (as indicated in the legend on the right) were separated by electrophoresis on a 4–12% SDS- polyacrylamide gel (Invitrogen). After migration, the gel was stained with PageBlue protein staining solution (Thermo Fisher Scientific).


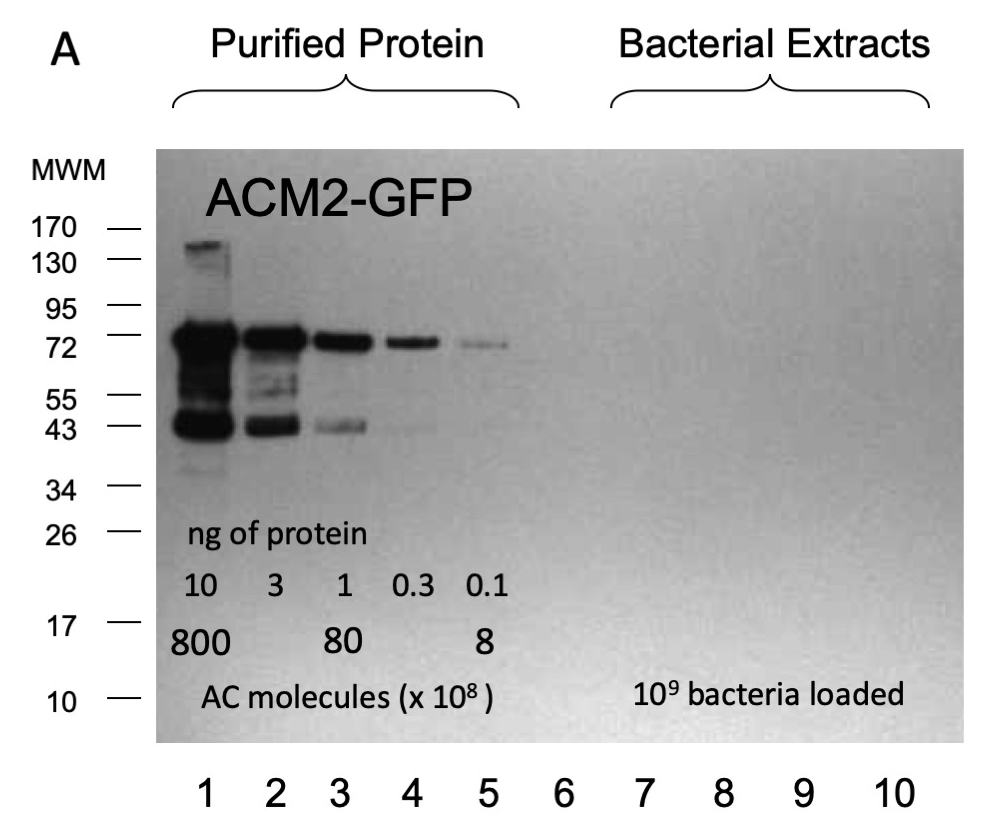


**Figure S4A:** Western blot analysis of the expression of the ACM2-GFP hybrid protein in DHM1. Lines 1 - 5 : 10, 3, 1, 0.3, and 0.1ng respectively of the purified ACM2-GFP hybrid protein (molecular weight of ≈ 73kDa; 0.1 ng of ACM2-GFP fusion correspond to ≈ 8 x 10^8^ protein molecules) were separated by electrophoresis, electro-transferred to nitrocellulose and detected with 3D1 monoclonal antibody. Line 6: Molecular Weight Markers (size in kDa indicated on the left side). Line 7: no protein. Lines 8-10 : Total bacterial extracts (corresponding to ≈ 10^9^ bacteria i.e. 1 ml of cell culture at OD_600_ = 1) from DHM1 cells harboring the indicated plasmids were probed in parallel by Western blot; line 8: pACM2-Gfp/pK1C-V_9A_; line 9: pACM2-Gfp/pK2C-V_9A_; line 10: pACM2- Gfp/pTCam-V_9A_.


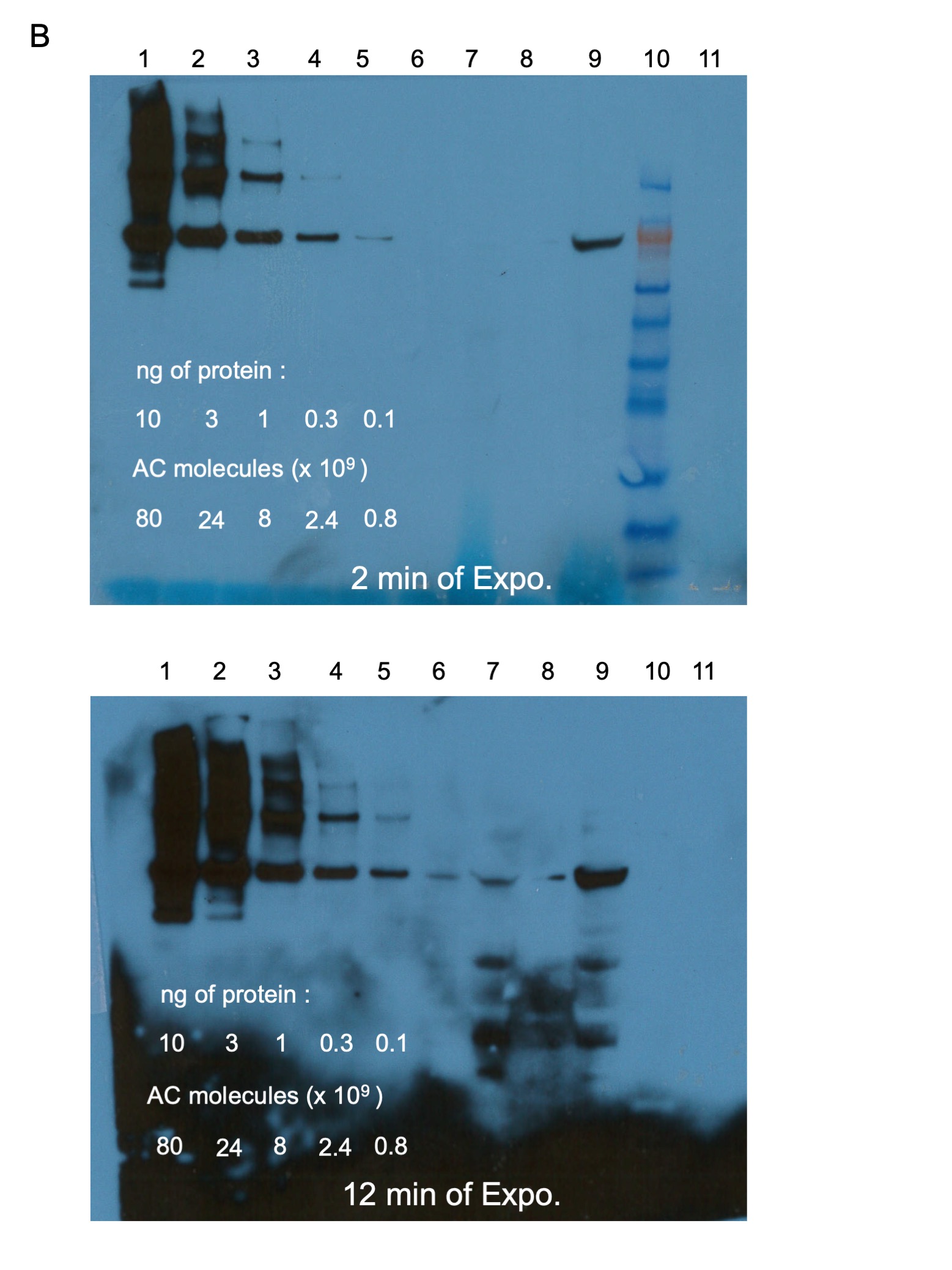


**Figure S4B:** Western blot analysis of the expression of the ACM2-GFP hybrid protein in DHM1. Lines 1 - 5 : 10, 3, 1, 0.3, and 0.1ng respectively of the purified ACM2-GFP hybrid protein (molecular weight of ≈ 73kDa; 0.1 ng of ACM2-GFP fusion correspond to ≈ 8 x 10^8^ protein molecules) were separated by electrophoresis, electro-transferred to nitrocellulose, detected with 3D1 monoclonal antibody and finally revealed by enhanced chemiluminescence (2 different times of exposition are shown). Line 6: empty (the small band in the 12 min exposure blot is a carry-over of line 5). Line 7: Total bacterial extract corresponding to ≈ 10^9^ bacteria i.e. 1 ml of cell culture at OD_600_ = 1 of DHM1/pAC0-Gfp/pK1CaM-V_9A_ ; Line 8: empty; Line 9 : Total bacterial extract as in line 7 (ie ≈ 10^9^ DHM1/pAC0-Gfp/pK1CaM-V_9A_ bacteria) supplemented with 0.5 ng of purified ACM2-GFP protein. Line 10: Molecular Weight Markers. Line 11 : no protein.

**
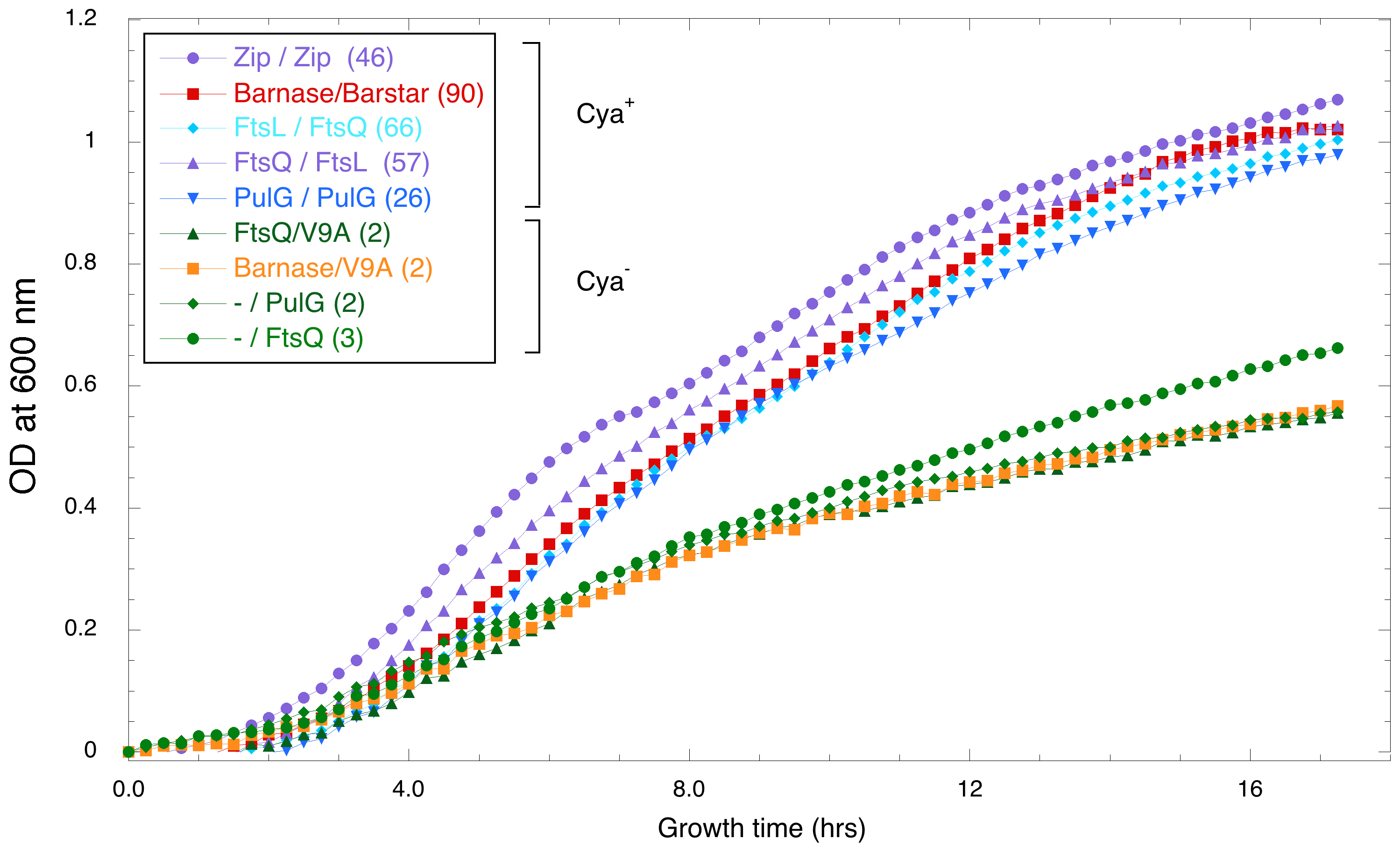
**

**Figure S5:** Growth kinetics of cells expressing different ACM2 and CaM fusions. DHM1 cells were transformed with the indicated couple of plasmids: X/Y corresponds to plasmids pACM2-X/pK1Cam-Y; for each couple, the mean values of β-galactosidase activities (with SD below 20 % of the mean) are shown in parenthesis. For each transformants, 7 independent colonies were picked up and grown at 30 °C in LB medium supplemented with IPTG and antibiotics, in a microplate reader with continuous agitation; OD at 600 nm was recorded every 15 min over 17 hrs. The curves shown are the mean growth of the 7 independent colonies. As can be seen, the growth of DHM1 cells co-expressing pACM2-Barnase and pK1Cam-V_9_A (orange symbol) is like that of cells expressing other non-interacting hybrids (e.g. Cya- phenotype). Similarly, the growth of DHM1 cells co-expressing pACM2-Barnase and pK1Cam-Barstar (red symbol) is like that of cells expressing other interacting hybrids (e.g. Cya+ phenotype). It should be noted that, as expected, Cya+ cells exhibit a better growth than Cya- cells, a phenomenon which is well documented (see ref 48).

**
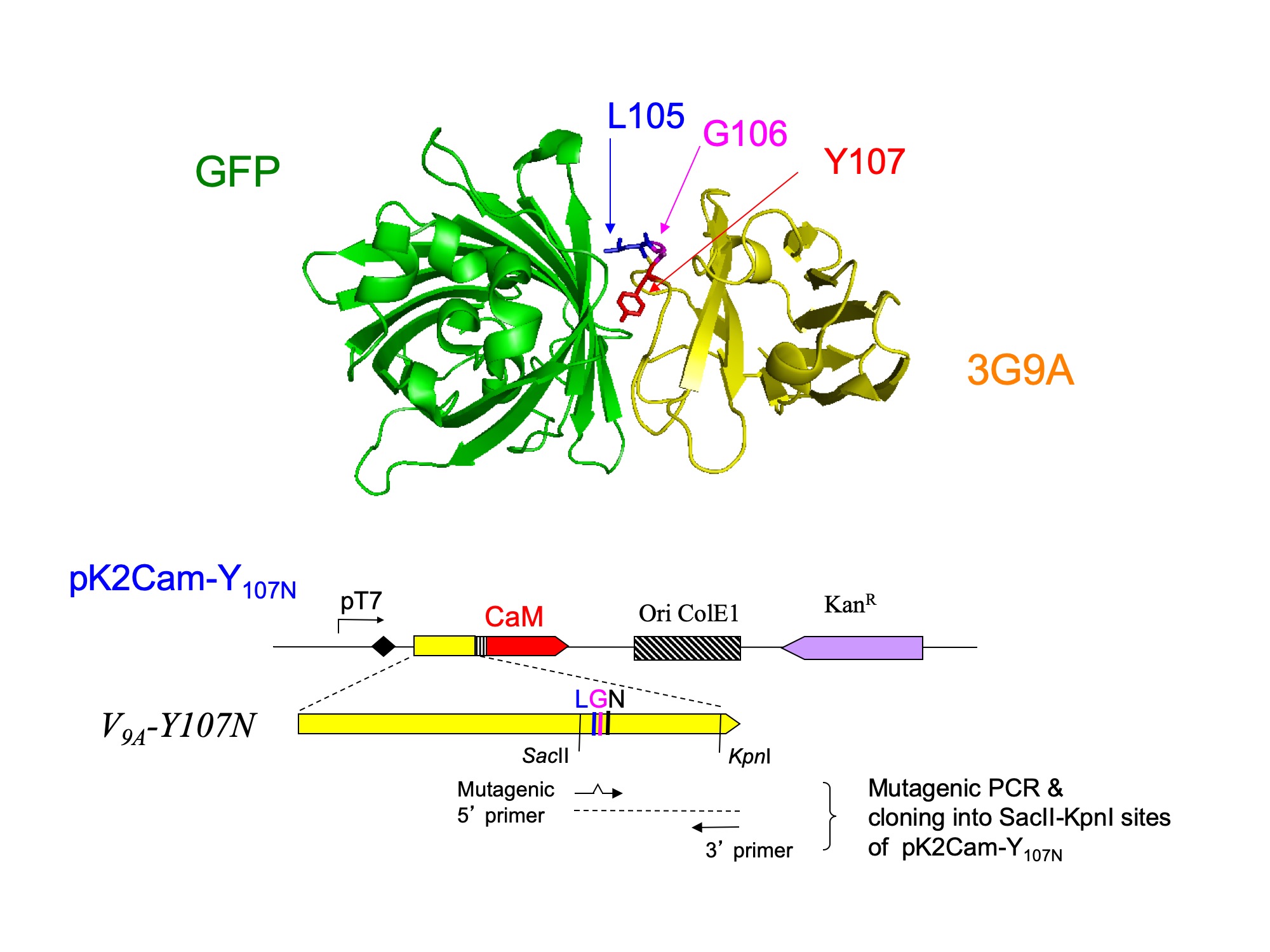
**

**Figure S6:** Mutagenesis of V_9A_ and selection of revertants on minimal medium. *Upper part:* 3D structure of the GFP/ V_9A_ complex (3G9A.pdb) showing the key L_105_, G_106_, and Y_107_ residues at the protein interface. *Lower part :* Schematic representation of the pK2Cam-V_9A_-Y_107N_ plasmid and mutagenesis strategy to modify the 105-107 residues. Briefly, pK2Cam-V_9A_-Y_107N_ was mutagenized by mutagenic PCR with an oligonucleotide primer containing degenerated codons NN(C/G) at position 105-107 as described in Materials and Methods. The mutagenized plasmid pool was then transformed into DHM1/pACM2-Gfp competent bacteria and plated on an indicator medium (LB supplemented with Xgal, IPTG, chloramphenicol and kanamycin) or on a selective medium (M63 minimal medium supplemented with maltose, Xgal, IPTG, chloramphenicol and kanamycin).

**Table S1:** Plasmids used in this work

**AC encoding plasmids**

| Plasmids | Vector features | Main characteristics | References |
| --- | --- | --- | --- |
| pDIA5240 | Amp  ColE1 *Ori* | expresses AC catalytic domain under the control of a lac promoter | Ladant *et al.* 1992 |
| pACM247 | Amp  ColE1 *Ori* | expresses ACM1 (ACM247) under the control of a lac promoter | Ladant *et al.* 1992 |
| pACM335 | Amp  ColE1 *Ori* | expresses ACM2 (ACM335) under the control of a lac promoter | Ladant *et al.* 1992 |
| pTRAC384GK | Amp  ColE1 *Ori* | expresses AC catalytic domain under the control of a thermoinducible λ promoter | Vougier *et al.* 2004 |
| pUT18C-zip | Amp  ColE1 *Ori* | expresses the T18 domain fused to the GCN4 leucine zipper (T18-zip) | Karimova *et al.* 2001 |
| pT25 | Cm  P15A *Ori* | expresses the T25 domain under the control of a pLac promoter | Karimova *et al.* 1998 |
| pAC0 | Cm  P15A *Ori* | expresses AC catalytic domain  No promoter, no RBS sequence | This work |
| pAC0-Gfp | Cm  P15A *Ori* | expresses AC-Gfp fusion  No promoter, no RBS sequence | This work |
| pACM1 | Cm  P15A *Ori* | expresses ACM1  No promoter, no RBS sequence | This work |
| pACM1-Gfp | Cm  P15A *Ori* | expresses ACM1-Gfp fusion  No promoter, no RBS sequence | This work |
| pACM1-Fkbp | Cm  P15A *Ori* | expresses ACM1-Fkbp fusion  No promoter, no RBS sequence | This work |
| pACM2-Gfp | Cm  P15A *Ori* | expresses ACM2-Gfp fusion  No promoter, no RBS sequence | This work |
| pACM2-Fkbp | Cm  P15A *Ori* | expresses ACM2-Fkbp fusion  No promoter, no RBS sequence | This work |
| pACM2-Zip | Cm  P15A *Ori* | expresses ACM2-Zip fusion  No promoter, no RBS sequence | This work |
| pACM2-TM-Zip | Cm  P15A *Ori* | expresses ACM2-TM-Zip fusion  No promoter, no RBS sequence | This work |
| pACM2-Barnase | Cm  P15A *Ori* | expresses ACM2-Barnase fusion  No promoter, no RBS sequence | This work |
| pACM2-FtsL | Cm  P15A *Ori* | expresses ACM2-FtsL (*E. coli*) fusion  No promoter, no RBS sequence | This work |
| pACM2-FtsQ | Cm  P15A *Ori* | expresses ACM2-FtsQ (*E. coli*) fusion  No promoter, no RBS sequence | This work |
| pACM2-FtsWpa | Cm  P15A *Ori* | expresses ACM2-FtsW (*P. aeruginosa*) fusion  No promoter, no RBS sequence | This work |
| pACM2-PulG | Cm  P15A *Ori* | expresses ACM2-PulG (*K. oxytoca*) fusion  No promoter, no RBS sequence | This work |
|  |  |  |  |
| pTrACM1-Fkbp | Amp  ColE1 *Ori* | expresses ACM1-Fkbp fusion under the control of a thermoinducible λ promoter | This work |
| pTrACM2-Fkbp | Amp  ColE1 *Ori* | expresses ACM2-Fkbp fusion under the control of a thermoinducible λ promoter | This work |
| pTrACM2-Gfp | Amp  ColE1 *Ori* | expresses ACM2-GFP fusion under the control of a thermoinducible λ promoter | This work |

**CaM encoding plasmids**

| Plasmids | Vector features | Main characteristics* | References |
| --- | --- | --- | --- |
|  | | | |
| pDLTCaM41 | Amp  ColE1 *Ori* | expresses CaM under the control of a thermoinducible λ promoter | Vougier *et al.* 2004 |
| pTCam | Amp  ColE1 *Ori* | Derives from pDLTCaM41  expresses CaM with a 6 x histidine tag at N-ter and a Tetracysteine tag at C-ter under control of a thermoinducible λ promoter | This work |
| pTCam-V_9A_ | Amp  ColE1 *Ori* | expresses CaM-V_9A_ fusion  under control of a thermoinducible λ promoter | This work |
| pTCam-V_9A_-Zip | Amp  ColE1 *Ori* | expresses CaM-V_9A_ with the 3D1 epitope and the GCN4 leucine zipper at Cter under control of a thermoinducible λ promoter | This work |
| pTCam-FRB | Amp  ColE1 *Ori* | expresses CaM-FRB fusion  under control of a thermoinducible λ promoter | This work |
| pK1Cam-V_9A_ | Kan  ColE1 *Ori* | expresses CaM-V_9A_ fusion  T7 promoter and a RBS sequence | This work |
| pK1Cam-V_9A_-Zip | Kan  ColE1 *Ori* | expresses CaM-V_9A_ with the 3D1 epitope and the GCN4 leucine zipper at Cter  T7 promoter and a RBS sequence | This work |
| pK1Cam-V_9A_-TM-Zip | Kan  ColE1 *Ori* | expresses CaM-V_9A_ with the 3D1 epitope followed by the OppB TM segment and the GCN4 leucine zipper at Cter  T7 promoter and a RBS sequence | This work |
| pK1Cam-Frb | Kan  ColE1 *Ori* | expresses CaM-FRB fusion  T7 promoter and a RBS sequence | This work |
| pK1Cam-Frb-Zs | Kan  ColE1 *Ori* | expresses CaM-FRB fusion  T7 promoter and a RBS sequence  ZsGreen under control of a pLac promoter | This work |
| pK1Cam-V_1K_ | Kan  ColE1 *Ori* | expresses CaM-V_1K_ fusion  T7 promoter and a RBS sequence | This work |
| pK1Cam-Barstar | Kan  ColE1 *Ori* | expresses CaM-Barstar fusion  T7 promoter and a RBS sequence | This work |
| pK1Cam-FtsL | Kan  ColE1 *Ori* | expresses CaM-FtsL (*E. coli*) fusion  T7 promoter and a RBS sequence | This work |
| pK1Cam-FtsQ | Kan  ColE1 *Ori* | expresses CaM-FtsQ (*E. coli*) fusion  T7 promoter and a RBS sequence | This work |
| pK1Cam-FtsIpa | Kan  ColE1 *Ori* | expresses CaM-FtsI (*P. aeruginosa*) fusion  T7 promoter and a RBS sequence | This work |
| pK1Cam-PulG | Kan  ColE1 *Ori* | expresses CaM-PulG (*K. oxytoca*) fusion  T7 promoter and a RBS sequence | This work |
|  |  |  |  |
| pK2Cam-V_9A_ | Kan  ColE1 *Ori* | expresses CaM-V_9A_ fusion  T7 promoter but no RBS sequence | This work |
| pK2Cam-V_9A_-Zip | Kan  ColE1 *Ori* | expresses CaM-V_9A_ with the 3D1 epitope and the GCN4 leucine zipper at Cter  T7 promoter but no RBS sequence | This work |
| pK2Cam-Y_107N_ | Kan  ColE1 *Ori* | expresses CaM-V_H3G_-Y_107N_ variant  with Y107N mutation  T7 promoter but no RBS sequence | This work |
| pK2Cam-Frb | Kan  ColE1 *Ori* | expresses CaM-FRB fusion  T7 promoter but no RBS sequence | This work |
| pK2Cam-V_1K_ | Kan  ColE1 *Ori* | expresses CaM-V_1K_ fusion  T7 promoter but no RBS sequence | This work |
| pK2Cam-Barstar | Kan  ColE1 *Ori* | expresses CaM-Barstar fusion  T7 promoter but no RBS sequence | This work |
| pCam_VU8_ | Amp  ColE1 *Ori* | expresses CaM_VU8_  T7 promoter and a RBS sequence | This work |
| pCam_VU8_-V_1K_ | Amp  ColE1 *Ori* | expresses CaM_VU8_-V_1K_ fusion  T7 promoter and a RBS sequence | This work |
| pCam_Cter_-V_1K_ | Amp  ColE1 *Ori* | expresses CaM_Cter_-V_1K_ fusion  T7 promoter and a RBS sequence | This work |
|  | | | |
| **Miscellaneous plasmids** | | | |
| pUC19 | Amp  ColE1 *Ori* | Cloning vector | Laboratory collection |
| pDSW207-blr | Amp  ColE1 *Ori* | expresses GFP fused to *E. coli* Blr | Karimova *et al.,* 2012 |
| pDL1312 | Amp  ColE1 *Ori* | expresses neurocalcin under the control of a thermoinducible λ promoter | Ladant *et al.,* 1995 |
| pMK-RQ | Kan  ColE1 *Ori* | Cloning vector | Geneart-Life-technologies |
| pZsGreen | Amp  ColE1 *Ori* | Contains the gene coding for ZsGreen, a variant of Zoanthus sp. green fluorescent protein | Clontech-Takara |

* Note : the *E. coli Δcya* strain DHM1 does not have T7 polymerase; the T7 promoter present in the CaM-expressing plasmid is therefore non-functional in this strain.

**Table S2:** Kinetic constants of the rapamycin-induced activation

of ACM-FKBP hybrids by CaM-FRB

| Experimental conditions | A_Max_ | K_D_ |
| --- | --- | --- |
| ACM1-FKBP |  |  |
| Ca^2+^ | 616 (42) | 749 (203) |
| Ca^2+^ + Rapamycin | 682 (21) | 1.3 (0.3) |
| EGTA | 78 (12) | > 5000 |
| EGTA + Rapamycin | 123 (10) | 2.3 (1.4) |
|  |  |  |
| ACM2-FKBP |  |  |
| Ca^2+^ | 701 (29) | 11.4 (3) |
| Ca^2+^ + Rapamycin | 687 (18) | 0.57 (0.11) |
| EGTA | 141 (23) | 196 (159) |
| EGTA + Rapamycin | 765 (14) | 1.1 (0.15) |
|  |  |  |

Maximal activities, A_Max_ , (in mol of cAMP per mol of enzyme per sec) and CaM-FRB concentrations (in nM) at half-maximal activation , ≈ K_D_ , were deduced from data shown in Figure 3 (standard errors are indicated in parentheses).

**Table S3:** Screening of V_9A_ variants on minimal medium

| Alleles | Nucleotide sequences  at codons 105-107 | Amino acid at residues 105-107 |
| --- | --- | --- |
| Wt | CTG GGT TAC | L G Y |
| V_9A_-M1 | CTG GGT TTC | L G **F** |
| V_9A_-M2 | AAC GGC TAC | **N** G Y |
| V_9A_-M3 | CAG GGC TAC | **Q** G Y |
| V_9A_-M4 | CTG GGT TAC | L G Y |
| V_9A_-M5 | CTC GGG TAC | L G Y |
| V_9A_-M6 | ATG GGC TAC | **M** G Y |
| V_9A_-M7 | TTG GGC TAC | L G Y |
| V_9A_-M8 | ATG GGG TAC | **M** G Y |
| V_9A_-M9 | TTG GGG TAC | L G Y |
| V_9A_-M10 | ATG GGC TAC | **M** G Y |

Plasmid pK2Cam-V_9A_-Y_107N_ was mutagenized at codon 105-107 by mutagenic PCR with oligonucleotide primers containing degenerated codons NN(C/G) at position 105-107 as described in Materials and Methods and as schematized in Figure S3. The mutagenized plasmid pool was transformed into DHM1/pAC2-Gfp competent bacteria and plated on a selective medium made of M63 minimal medium supplemented with maltose, Xgal, IPTG, chloramphenicol and kanamycin. Plates were grown for 7-10 days at 30 °C. Few growing colonies (i.e., exhibiting a Cya^+^ phenotype, i.e. Lac^+^ and Mal^+^) were randomly picked for plasmid DNA purification and the pK2Cam plasmids were sequenced.

**Appendix 1**: DNA sequences of main ESACH plasmids

AC fusion encoding plasmids

pAC0

pAC0-Gfp

pACM1-Gfp

pACM2-Fkbp

pACM2-Zip

pACM2-TM-Zip

pACM2-Barnase

pTrACM2-Gfp

CaM fusion encoding plasmids

pTCam

pTCam-V_9A_

pTCam-V_9A_-Zip

pTCam-Frb

pK1Cam-V_9A_

pK2Cam-V_9A_

pK1Cam-V_9A_-Zip

pK1Cam-V_9A_-TM-Zip

pK1Cam-Frb

pK1Cam-Frb-Zs

pK1Cam-V_1K_

pK1Cam-Barstar

pCam_VU8_

pCam_VU8_-V_1K_

pCam_Cter_-V_1K_

**pAC0 (3610 bp):**

**Map**

**
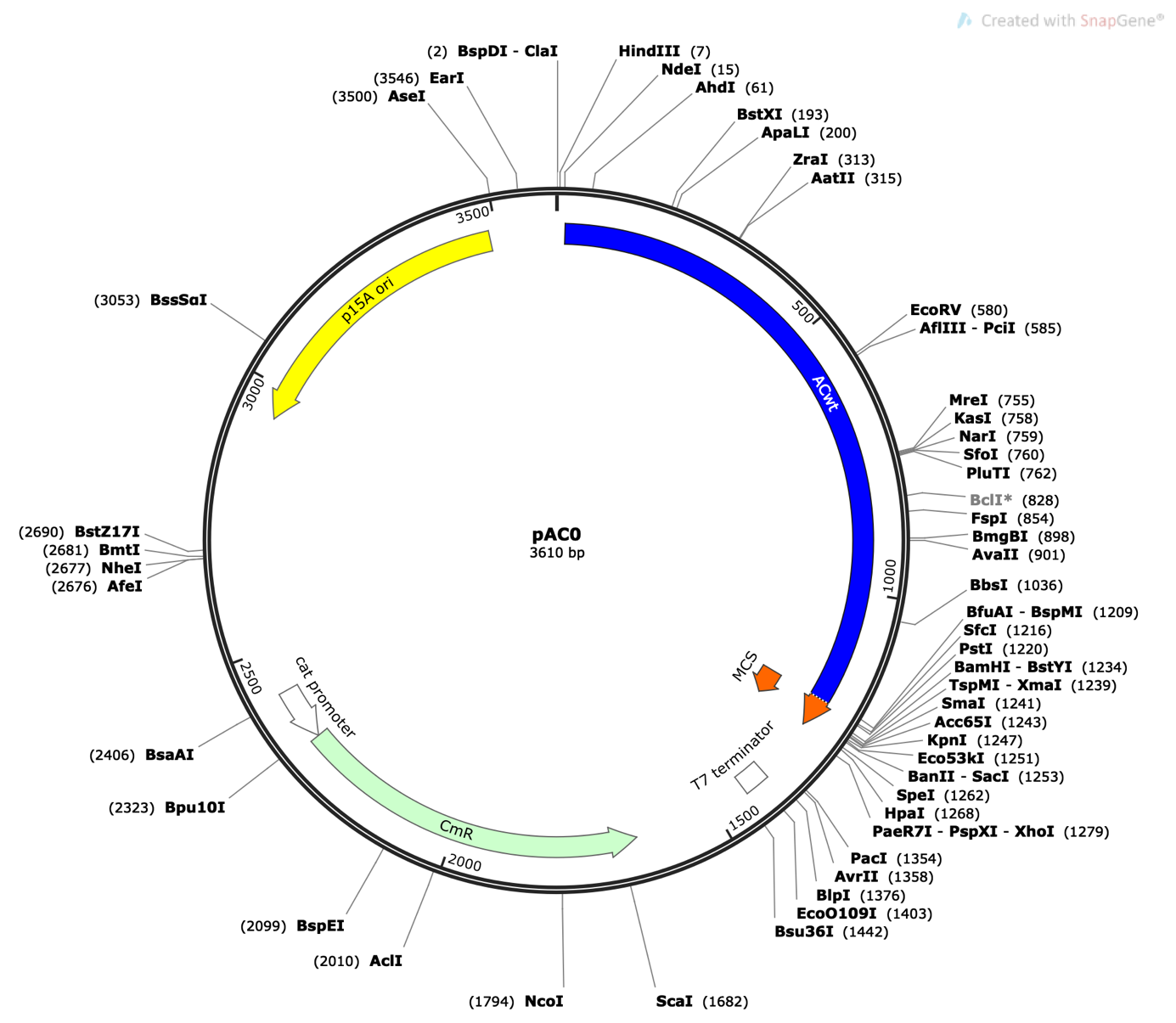
**

**pAC0 full DNA sequence:**

**atcgat**aagcttgcatATGCAGCAATCGCATCAGGCTGGTTACGCAAACGCCGCCGACCGGGAGTCTGGCATCCCCGCAGCCGTACTCGATGGCATCAAGGCCGTGGCGAAGGAAAAAAACGCCACATTGATGTTCCGCCTGGTCAACCCCCATTCCACCAGCCTGATTGCCGAAGGGGTGGCCACCAAAGGATTGGGCGTGCACGCCAAGTCGTCCGATTGGGGGTTGCAGGCGGGCTACATTCCCGTCAACCCGAATCTTTCCAAACTGTTCGGCCGTGCGCCCGAGGTGATCGCGCGGGCCGACAACGACGTCAACAGCAGCCTGGCGCATGGCCATACCGCGGTCGACCTGACGCTGTCGAAAGAGCGGCTTGACTATCTGCGGCAAGCGGGCCTGGTCACCGGCATGGCCGATGGCGTGGTCGCGAGCAACCACGCAGGCTACGAGCAGTTCGAGTTTCGCGTGAAGGAAACCTCGGACGGGCGCTATGCCGTGCAGTATCGCCGCAAGGGCGGCGACGATTTCGAGGCGGTCAAGGTGATCGGCAATGCCGCCGGTATTCCACTGACGGCGGATATCGACATGTTCGCCATTATGCCGCATCTGTCCAACTTCCGCGACTCGGCGCGCAGTTCGGTGACCAGCGGCGATTCGGTGACCGATTACCTGGCGCGCACGCGGCGGGCCGCCAGCGAGGCCACGGGCGGCCTGGATCGCGAACGCATCGACTTGTTGTGGAAAATCGCTCGCGCCGGCGCCCGTTCCGCAGTGGGCACCGAGGCGCGTCGCCAGTTCCGCTACGACGGCGACATGAATATCGGCGTGATCACCGATTTCGAGCTGGAAGTGCGCAATGCGCTGAACAGGCGGGCGCACGCCGTCGGCGCGCAGGACGTGGTCCAGCATGGCACTGAGCAGAACAATCCTTTCCCGGAGGCAGATGAGAAGATTTTCGTCGTATCGGCCACCGGTGAAAGCCAGATGCTCACGCGCGGGCAACTGAAGGAATACATTGGCCAGCAGCGCGGCGAGGGCTATGTCTTCTACGAGAACCGTGCATACGGCGTGGCGGGGAAAAGCCTGTTCGACGATGGGCTGGGAGCCGCGCCCGGCGTGCCGAGCGGACGTTCGAAGTTCTCGCCGGATGTACTGGAAACGGTGCCGGCGTCACCCGGATTGCGGCGGCCGTCGCTGGGCGCAGTGGAACGCCACTGCAGGTCGACTCTAGAGGATCCCCGGGTACCGAGCTCGAATTCTACTAGTTAActaagtaa**ctcgag**tctggtaaagaaaccgctgctgcgaaatttgaacgccagcacatggactcgtctactagcgcagcttaattaacctaggctgctgccaccgctgagcaataactagcataaccccttggggcctctaaacgggtcttgaggggttttttgctgaaacctcaggcatttgagaagcacacggtcacactgcttccggtagtcaataaaccggtaaaccagcaatagacataagcggctatttaacgaccctgccctgaaccgacgaccgggtcgaatttgctttcgaatttctgccattcatccgcttattatcacttattcaggcgtagcaccaggcgtttaagggcaccaataactgccttaaaaaaattacgccccgccctgccactcatcgcagtactgttgtaattcattaagcattctgccgacatggaagccatcacagacggcatgatgaacctgaatcgccagcggcatcagcaccttgtcgccttgcgtataatatttgcccatggtgaaaacgggggcgaagaagttgtccatattggccacgtttaaatcaaaactggtgaaactcacccagggattggctgagacgaaaaacatattctcaataaaccctttagggaaataggccaggttttcaccgtaacacgccacatcttgcgaatatatgtgtagaaactgccggaaatcgtcgtggtattcactccagagcgatgaaaacgtttcagtttgctcatggaaaacggtgtaacaagggtgaacactatcccatatcaccagctcaccgtctttcattgccatacggaattccggatgagcattcatcaggcgggcaagaatgtgaataaaggccggataaaacttgtgcttatttttctttacggtctttaaaaaggccgtaatatccagctgaacggtctggttataggtacattgagcaactgactgaaatgcctcaaaatgttctttacgatgccattgggatatatcaacggtggtatatccagtgatttttttctccattttagcttccttagctcctgaaaatctcgataactcaaaaaatacgcccggtagtgatcttatttcattatggtgaaagttggaacctcttacgtgccgatcaacgtctcattttcgccaaaagttggcccagggcttcccggtatcaacagggacaccaggatttatttattctgcgaagtgatcttccgtcacaggtatttattcggcgcaaagtgcgtcgggtgatgctgccaacttactgatttagtgtatgatggtgtttttgaggtgctccagtggcttctgtttctatcagctgtccctcctgttcagctactgacggggtggtgcgtaacggcaaaagcaccgccggacatcagcgctagcggagtgtatactggcttactatgttggcactgatgagggtgtcagtgaagtgcttcatgtggcaggagaaaaaaggctgcaccggtgcgtcagcagaatatgtgatacaggatatattccgcttcctcgctcactgactcgctacgctcggtcgttcgactgcggcgagcggaaatggcttacgaacggggcggagatttcctggaagatgccaggaagatacttaacagggaagtgagagggccgcggcaaagccgtttttccataggctccgcccccctgacaagcatcacgaaatctgacgctcaaatcagtggtggcgaaacccgacaggactataaagataccaggcgtttcccctggcggctccctcgtgcgctctcctgttcctgcctttcggtttaccggtgtcattccgctgttatggccgcgtttgtctcattccacgcctgacactcagttccgggtaggcagttcgctccaagctggactgtatgcacgaaccccccgttcagtccgaccgctgcgccttatccggtaactatcgtcttgagtccaacccggaaagacatgcaaaagcaccactggcagcagccactggtaattgatttagaggagttagtcttgaagtcatgcgccggttaaggctaaactgaaaggacaagttttggtgactgcgctcctccaagccagttacctcggttcaaagagttggtagctcagagaaccttcgaaaaaccgccctgcaaggcggttttttcgttttcagagcaagagattacgcgcagaccaaaacgatctcaagaagatcatcttattaatcagataaaatatttctagatttcagtgcaatttatctcttcaaatgtagcacctgaagtcagccccatacgatataagttgtaattctcatgtttgacagcttatc

**pAC0-GFP (4337 bp):**


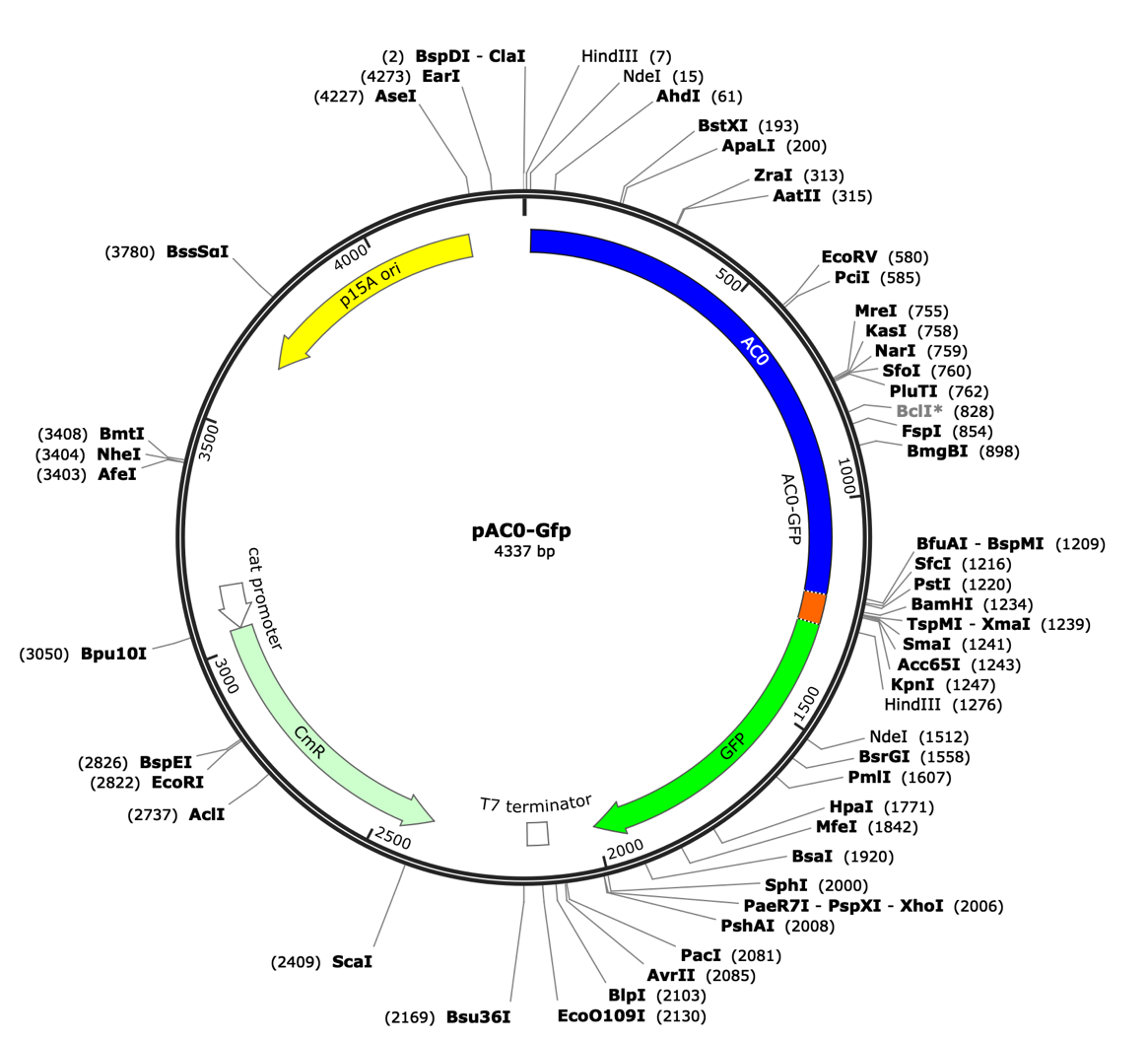


**DNA sequence (*Cla*I-*Xho*I fragment)**

**atcgat**aagcttgcatATGCAGCAATCGCATCAGGCTGGTTACGCAAACGCCGCCGACCGGGAGTCTGGCATCCCCGCAGCCGTACTCGATGGCATCAAGGCCGTGGCGAAGGAAAAAAACGCCACATTGATGTTCCGCCTGGTCAACCCCCATTCCACCAGCCTGATTGCCGAAGGGGTGGCCACCAAAGGATTGGGCGTGCACGCCAAGTCGTCCGATTGGGGGTTGCAGGCGGGCTACATTCCCGTCAACCCGAATCTTTCCAAACTGTTCGGCCGTGCGCCCGAGGTGATCGCGCGGGCCGACAACGACGTCAACAGCAGCCTGGCGCATGGCCATACCGCGGTCGACCTGACGCTGTCGAAAGAGCGGCTTGACTATCTGCGGCAAGCGGGCCTGGTCACCGGCATGGCCGATGGCGTGGTCGCGAGCAACCACGCAGGCTACGAGCAGTTCGAGTTTCGCGTGAAGGAAACCTCGGACGGGCGCTATGCCGTGCAGTATCGCCGCAAGGGCGGCGACGATTTCGAGGCGGTCAAGGTGATCGGCAATGCCGCCGGTATTCCACTGACGGCGGATATCGACATGTTCGCCATTATGCCGCATCTGTCCAACTTCCGCGACTCGGCGCGCAGTTCGGTGACCAGCGGCGATTCGGTGACCGATTACCTGGCGCGCACGCGGCGGGCCGCCAGCGAGGCCACGGGCGGCCTGGATCGCGAACGCATCGACTTGTTGTGGAAAATCGCTCGCGCCGGCGCCCGTTCCGCAGTGGGCACCGAGGCGCGTCGCCAGTTCCGCTACGACGGCGACATGAATATCGGCGTGATCACCGATTTCGAGCTGGAAGTGCGCAATGCGCTGAACAGGCGGGCGCACGCCGTCGGCGCGCAGGACGTGGTCCAGCATGGCACTGAGCAGAACAATCCTTTCCCGGAGGCAGATGAGAAGATTTTCGTCGTATCGGCCACCGGTGAAAGCCAGATGCTCACGCGCGGGCAACTGAAGGAATACATTGGCCAGCAGCGCGGCGAGGGCTATGTCTTCTACGAGAACCGTGCATACGGCGTGGCGGGGAAAAGCCTGTTCGACGATGGGCTGGGAGCCGCGCCCGGCGTGCCGAGCGGACGTTCGAAGTTCTCGCCGGATGTACTGGAAACGGTGCCGGCGTCACCCGGATTGCGGCGGCCGTCGCTGGGCGCAGTGGAACGCCACTGCAGGTCGACTCTAGAGGATCCCCGGGTACCGGGGGGGTCTATGACCATGATTACGCCAAGCTTGATGAGTAAAGGAGAAGAACTTTTCACTGGAGTTGTCCCAATTCTTGTTGAATTAGATGGTGATGTTAATGGGCACAAATTTTCTGTCAGTGGAGAGGGTGAAGGTGATGCAACATACGGAAAACTTACCCTTAAATTTATTTGCACTACTGGAAAACTACCTGTTCCATGGCCAACACTTGTCACTACTTTCGCGTATGGTCTTCAATGCTTTGCGAGATACCCAGATCATATGAAACAGCATGACTTTTTCAAGAGTGCCATGCCCGAAGGTTATGTACAGGAAAGAACTATATTTTTCAAAGATGACGGGAACTACAAGACACGTGCTGAAGTCAAGTTTGAAGGTGATACCCTTGTTAATAGAATCGAGTTAAAAGGTATTGATTTTAAAGAAGATGGAAACATTCTTGGACACAAATTGGAATACAACTATAACTCACACAATGTATACATCATGGCAGACAAACAAAAGAATGGAATCAAAGTTAACTTCAAAATTAGACACAACATTGAAGATGGAAGCGTTCAACTAGCAGACCATTATCAACAAAATACTCCAATTGGCGATGGCCCTGTCCTTTTACCAGACAACCATTACCTGTCCACACAATCTGCCCTTTCGAAAGATCCCAACGAAAAGAGAGACCACATGGTCCTTCTTGAGTTTGTAACAGCTGCTGGGATTACACATGGCATGGATGAACTATACAAGCATGCGTGA**ctcgag**

**pACM1-GFP (4343 bp):**


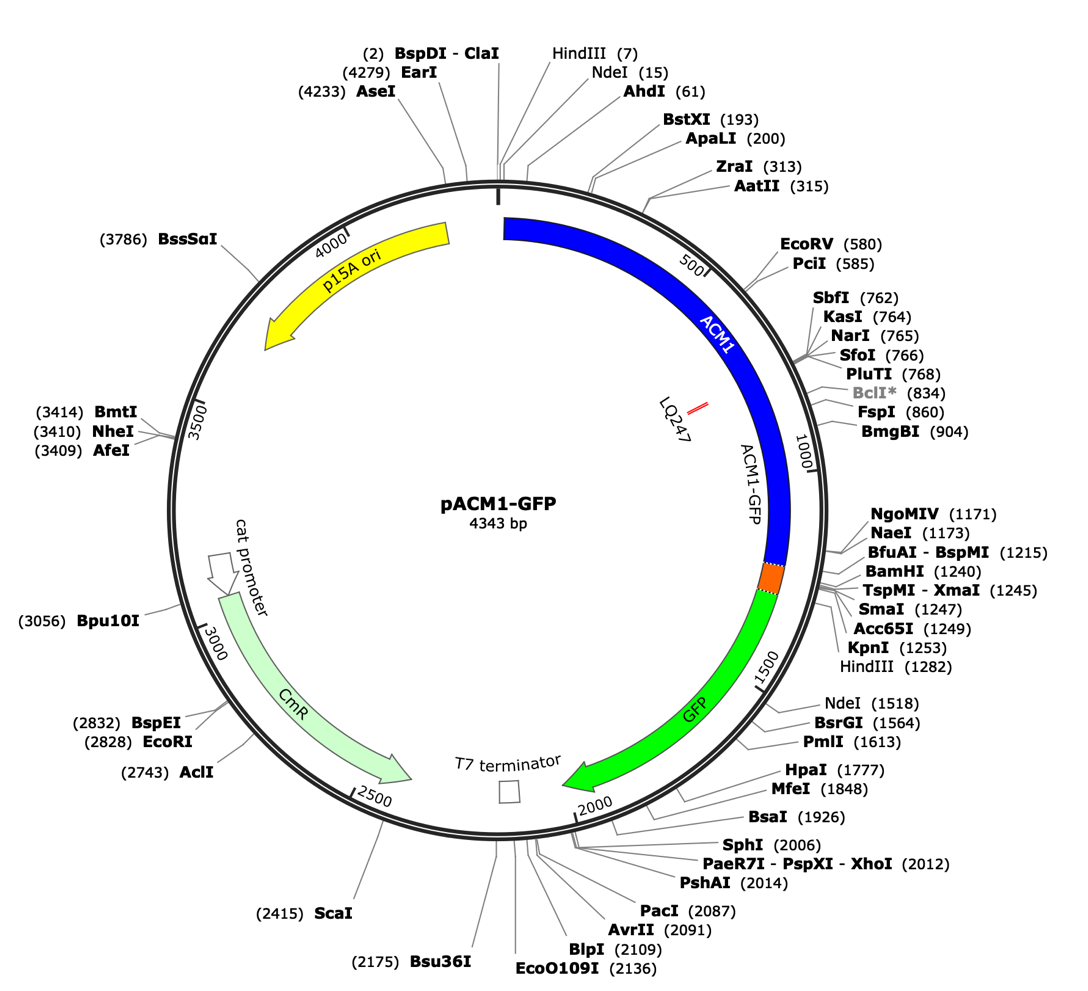


**DNA sequence (*Cla*I-*Xho*I fragment)**

**atcgat**aagcttgcatATGCAGCAATCGCATCAGGCTGGTTACGCAAACGCCGCCGACCGGGAGTCTGGCATCCCCGCAGCCGTACTCGATGGCATCAAGGCCGTGGCGAAGGAAAAAAACGCCACATTGATGTTCCGCCTGGTCAACCCCCATTCCACCAGCCTGATTGCCGAAGGGGTGGCCACCAAAGGATTGGGCGTGCACGCCAAGTCGTCCGATTGGGGGTTGCAGGCGGGCTACATTCCCGTCAACCCGAATCTTTCCAAACTGTTCGGCCGTGCGCCCGAGGTGATCGCGCGGGCCGACAACGACGTCAACAGCAGCCTGGCGCATGGCCATACCGCGGTCGACCTGACGCTGTCGAAAGAGCGGCTTGACTATCTGCGGCAAGCGGGCCTGGTCACCGGCATGGCCGATGGCGTGGTCGCGAGCAACCACGCAGGCTACGAGCAGTTCGAGTTTCGCGTGAAGGAAACCTCGGACGGGCGCTATGCCGTGCAGTATCGCCGCAAGGGCGGCGACGATTTCGAGGCGGTCAAGGTGATCGGCAATGCCGCCGGTATTCCACTGACGGCGGATATCGACATGTTCGCCATTATGCCGCATCTGTCCAACTTCCGCGACTCGGCGCGCAGTTCGGTGACCAGCGGCGATTCGGTGACCGATTACCTGGCGCGCACGCGGCGGGCCGCCAGCGAGGCCACGGGCGGCCTGGATCGCGAACGCATCGACTTGTTGTGGAAAATCGCTCGCGCC**CTGCAG**GGCGCCCGTTCCGCAGTGGGCACCGAGGCGCGTCGCCAGTTCCGCTACGACGGCGACATGAATATCGGCGTGATCACCGATTTCGAGCTGGAAGTGCGCAATGCGCTGAACAGGCGGGCGCACGCCGTCGGCGCGCAGGACGTGGTCCAGCATGGCACTGAGCAGAACAATCCTTTCCCGGAGGCAGATGAGAAGATTTTCGTCGTATCGGCCACCGGTGAAAGCCAGATGCTCACGCGCGGGCAACTGAAGGAATACATTGGCCAGCAGCGCGGCGAGGGCTATGTCTTCTACGAGAACCGTGCATACGGCGTGGCGGGGAAAAGCCTGTTCGACGATGGGCTGGGAGCCGCGCCCGGCGTGCCGAGCGGACGTTCGAAGTTCTCGCCGGATGTACTGGAAACGGTGCCGGCGTCACCCGGATTGCGGCGGCCGTCGCTGGGCGCAGTGGAACGCCACTGCAGGTCGACTCTAGAGGATCCCCGGGTACCGGGGGGGTCTATGACCATGATTACGCCAAGCTTGATGAGTAAAGGAGAAGAACTTTTCACTGGAGTTGTCCCAATTCTTGTTGAATTAGATGGTGATGTTAATGGGCACAAATTTTCTGTCAGTGGAGAGGGTGAAGGTGATGCAACATACGGAAAACTTACCCTTAAATTTATTTGCACTACTGGAAAACTACCTGTTCCATGGCCAACACTTGTCACTACTTTCGCGTATGGTCTTCAATGCTTTGCGAGATACCCAGATCATATGAAACAGCATGACTTTTTCAAGAGTGCCATGCCCGAAGGTTATGTACAGGAAAGAACTATATTTTTCAAAGATGACGGGAACTACAAGACACGTGCTGAAGTCAAGTTTGAAGGTGATACCCTTGTTAATAGAATCGAGTTAAAAGGTATTGATTTTAAAGAAGATGGAAACATTCTTGGACACAAATTGGAATACAACTATAACTCACACAATGTATACATCATGGCAGACAAACAAAAGAATGGAATCAAAGTTAACTTCAAAATTAGACACAACATTGAAGATGGAAGCGTTCAACTAGCAGACCATTATCAACAAAATACTCCAATTGGCGATGGCCCTGTCCTTTTACCAGACAACCATTACCTGTCCACACAATCTGCCCTTTCGAAAGATCCCAACGAAAAGAGAGACCACATGGTCCTTCTTGAGTTTGTAACAGCTGCTGGGATTACACATGGCATGGATGAACTATACAAGCATGCGTGA**ctcgag**

**pACM2-Fkbp (3914 bp):**


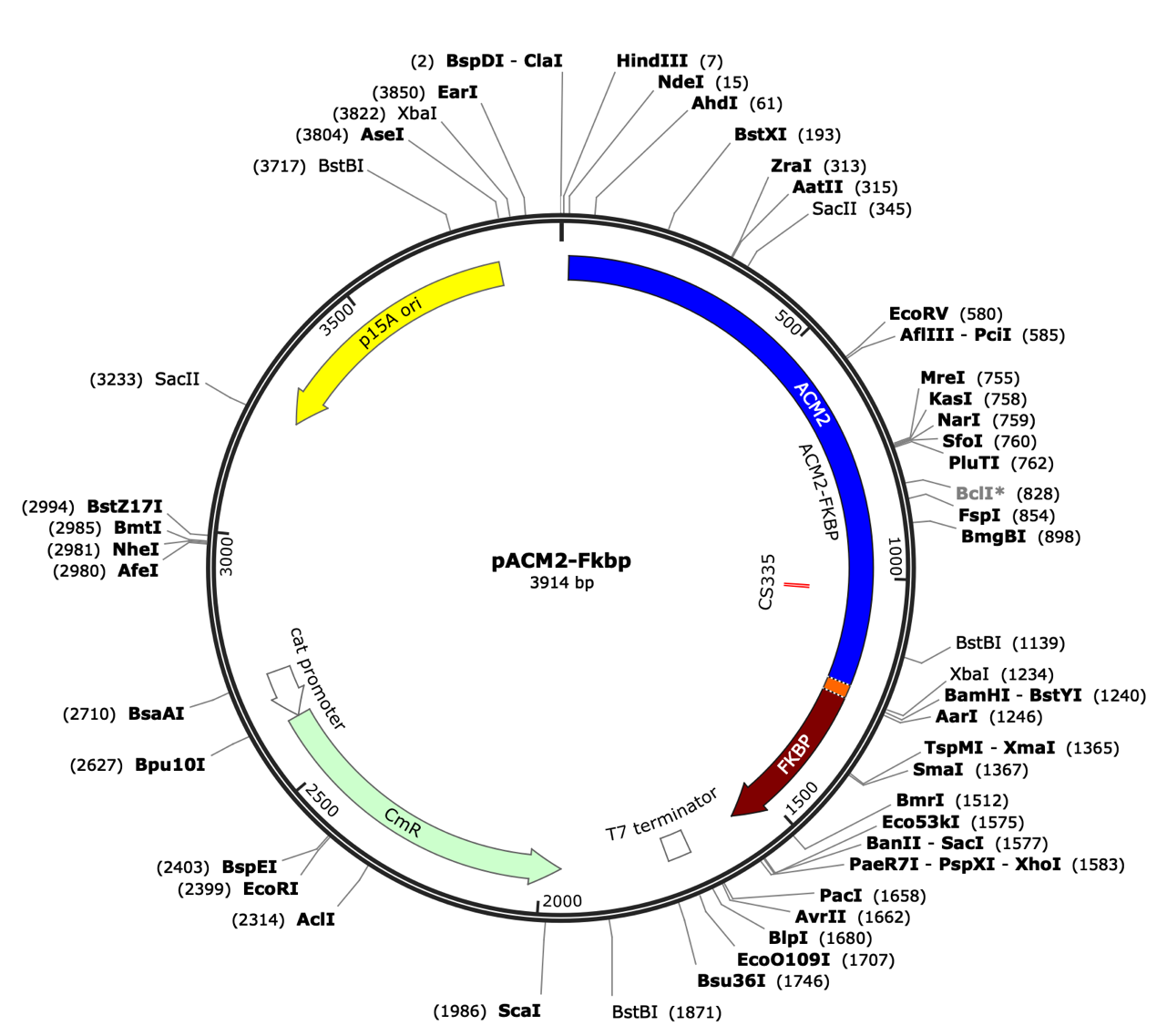


**DNA sequence (*Cla*I-*Xho*I fragment)**

**atcgat**aagcttgcatATGCAGCAATCGCATCAGGCTGGTTACGCAAACGCCGCCGACCGGGAGTCTGGCATCCCCGCAGCCGTACTCGATGGCATCAAGGCCGTGGCGAAGGAAAAAAACGCCACATTGATGTTCCGCCTGGTCAACCCCCATTCCACCAGCCTGATTGCCGAAGGGGTGGCCACCAAAGGATTGGGCGTGCACGCCAAGTCGTCCGATTGGGGGTTGCAGGCGGGCTACATTCCCGTCAACCCGAATCTTTCCAAACTGTTCGGCCGTGCGCCCGAGGTGATCGCGCGGGCCGACAACGACGTCAACAGCAGCCTGGCGCATGGCCATACCGCGGTCGACCTGACGCTGTCGAAAGAGCGGCTTGACTATCTGCGGCAAGCGGGCCTGGTCACCGGCATGGCCGATGGCGTGGTCGCGAGCAACCACGCAGGCTACGAGCAGTTCGAGTTTCGCGTGAAGGAAACCTCGGACGGGCGCTATGCCGTGCAGTATCGCCGCAAGGGCGGCGACGATTTCGAGGCGGTCAAGGTGATCGGCAATGCCGCCGGTATTCCACTGACGGCGGATATCGACATGTTCGCCATTATGCCGCATCTGTCCAACTTCCGCGACTCGGCGCGCAGTTCGGTGACCAGCGGCGATTCGGTGACCGATTACCTGGCGCGCACGCGGCGGGCCGCCAGCGAGGCCACGGGCGGCCTGGATCGCGAACGCATCGACTTGTTGTGGAAAATCGCTCGCGCCGGCGCCCGTTCCGCAGTGGGCACCGAGGCGCGTCGCCAGTTCCGCTACGACGGCGACATGAATATCGGCGTGATCACCGATTTCGAGCTGGAAGTGCGCAATGCGCTGAACAGGCGGGCGCACGCCGTCGGCGCGCAGGACGTGGTCCAGCATGGCACTGAGCAGAACAATCCTTTCCCGGAGGCAGATGAGAAGATTTTCGTCGTATCGGCCACCGGTGAAAGCCAGATGCTCACGCGCGGGCAACTGAAGGAATACATTGGC**TGCAGC**CAGCAGCGCGGCGAGGGCTATGTCTTCTACGAGAACCGTGCATACGGCGTGGCGGGGAAAAGCCTGTTCGACGATGGGCTGGGAGCCGCGCCCGGCGTGCCGAGCGGACGTTCGAAGTTCTCGCCGGATGTACTGGAAACGGTGCCGGCGTCACCCGGATTGCGGCGGCCGTCGCTGGGCGCAGTGGAACGCCACTGCAGGTCGACTCTAGAGGATCCCATGGGCGTGCAGGTGGAGACTATCTCCCCAGGAGACGGGCGCACCTTCCCCAAGCGCGGCCAGACCTGCGTGGTGCACTACACCGGGATGCTTGAAGATGGAAAGAAATTTGATTCCTCCCGGGACAGAAACAAGCCCTTTAAGTTTATGCTAGGCAAGCAGGAGGTGATCCGAGGCTGGGAAGAAGGGGTTGCCCAGATGAGTGTGGGTCAGAGAGCCAAACTGACTATATCTCCAGATTATGCCTATGGTGCCACTGGGCACCCAGGCATCATCCCACCACATGCCACTCTCGTCTTCGATGTGGAGCTTCTAAAACTGGAACGGAGCTCTTAA**ctcgag**

**pACM2-Zip ( 3743 bp):**

**
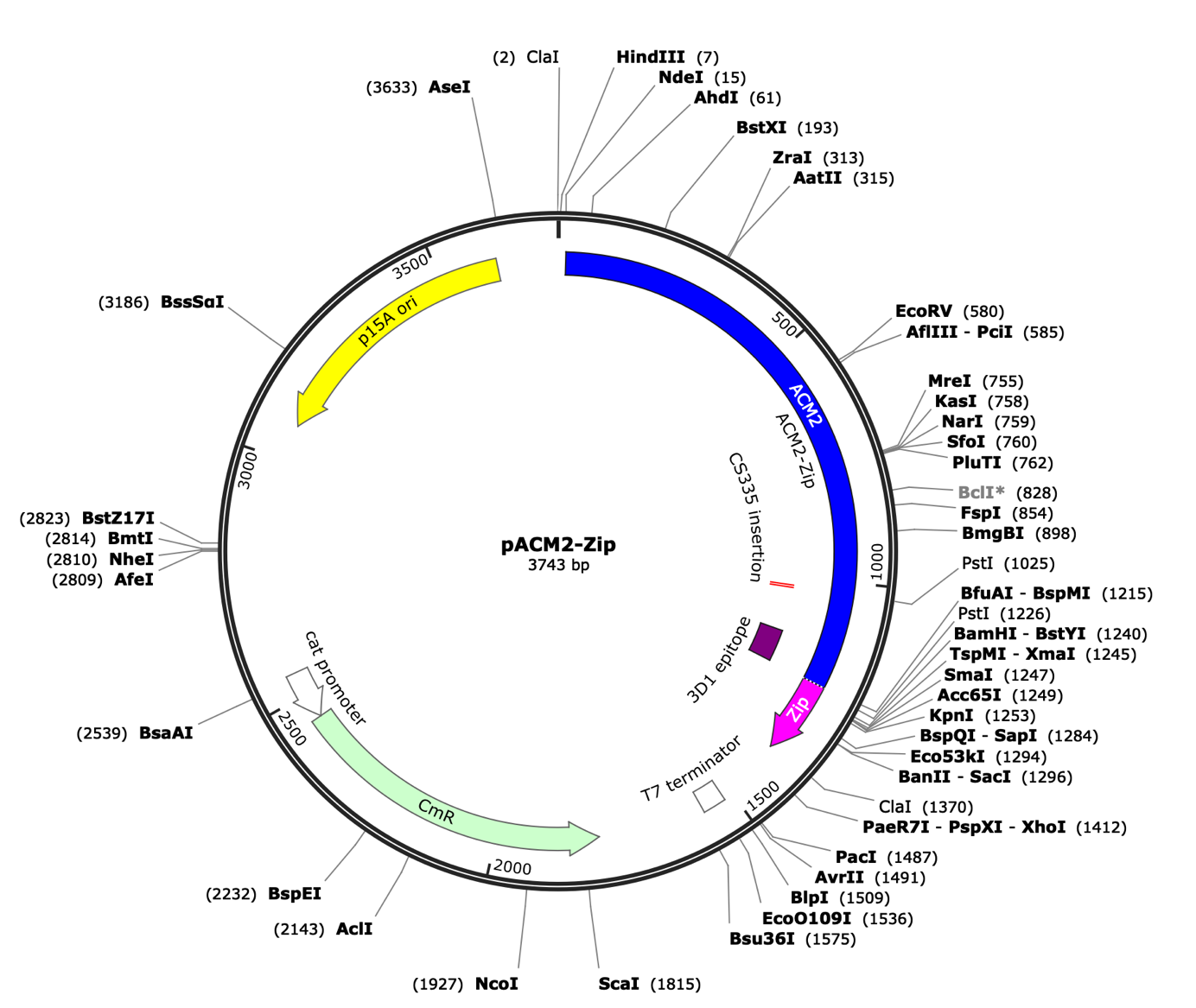
**

**DNA sequence (*Cla*I-*Xho*I fragment)**

**atcgat**aagcttgcatATGCAGCAATCGCATCAGGCTGGTTACGCAAACGCCGCCGACCGGGAGTCTGGCATCCCCGCAGCCGTACTCGATGGCATCAAGGCCGTGGCGAAGGAAAAAAACGCCACATTGATGTTCCGCCTGGTCAACCCCCATTCCACCAGCCTGATTGCCGAAGGGGTGGCCACCAAAGGATTGGGCGTGCACGCCAAGTCGTCCGATTGGGGGTTGCAGGCGGGCTACATTCCCGTCAACCCGAATCTTTCCAAACTGTTCGGCCGTGCGCCCGAGGTGATCGCGCGGGCCGACAACGACGTCAACAGCAGCCTGGCGCATGGCCATACCGCGGTCGACCTGACGCTGTCGAAAGAGCGGCTTGACTATCTGCGGCAAGCGGGCCTGGTCACCGGCATGGCCGATGGCGTGGTCGCGAGCAACCACGCAGGCTACGAGCAGTTCGAGTTTCGCGTGAAGGAAACCTCGGACGGGCGCTATGCCGTGCAGTATCGCCGCAAGGGCGGCGACGATTTCGAGGCGGTCAAGGTGATCGGCAATGCCGCCGGTATTCCACTGACGGCGGATATCGACATGTTCGCCATTATGCCGCATCTGTCCAACTTCCGCGACTCGGCGCGCAGTTCGGTGACCAGCGGCGATTCGGTGACCGATTACCTGGCGCGCACGCGGCGGGCCGCCAGCGAGGCCACGGGCGGCCTGGATCGCGAACGCATCGACTTGTTGTGGAAAATCGCTCGCGCCGGCGCCCGTTCCGCAGTGGGCACCGAGGCGCGTCGCCAGTTCCGCTACGACGGCGACATGAATATCGGCGTGATCACCGATTTCGAGCTGGAAGTGCGCAATGCGCTGAACAGGCGGGCGCACGCCGTCGGCGCGCAGGACGTGGTCCAGCATGGCACTGAGCAGAACAATCCTTTCCCGGAGGCAGATGAGAAGATTTTCGTCGTATCGGCCACCGGTGAAAGCCAGATGCTCACGCGCGGGCAACTGAAGGAATACATTGGC**TGCAGC**CAGCAGCGCGGCGAGGGCTATGTCTTCTACGAGAACCGTGCATACGGCGTGGCGGGGAAAAGCCTGTTCGACGATGGGCTGGGAGCCGCGCCCGGCGTGCCGAGCGGACGTTCGAAGTTCTCGCCGGATGTACTGGAAACGGTGCCGGCGTCACCCGGATTGCGGCGGCCGTCGCTGGGCGCAGTGGAACGCCACTGCAGGTCGACTCTAGAGGATCCCCGGGTACCTATCCAGCGTATGAAACAGCTGGAAGACAAAGTTGAAGAGCTCCTGAGCAAAAACTACCACCTGGAGAACGAAGTTGCGCGCCTGAAAAAACTGGTGGGTGAACGTGGGAATTCATCGATATAActaagtaatatggtgcactctcagtacaatctg**ctcgag**

**pACM2-TM-Zip ( 3869 bp):**

**
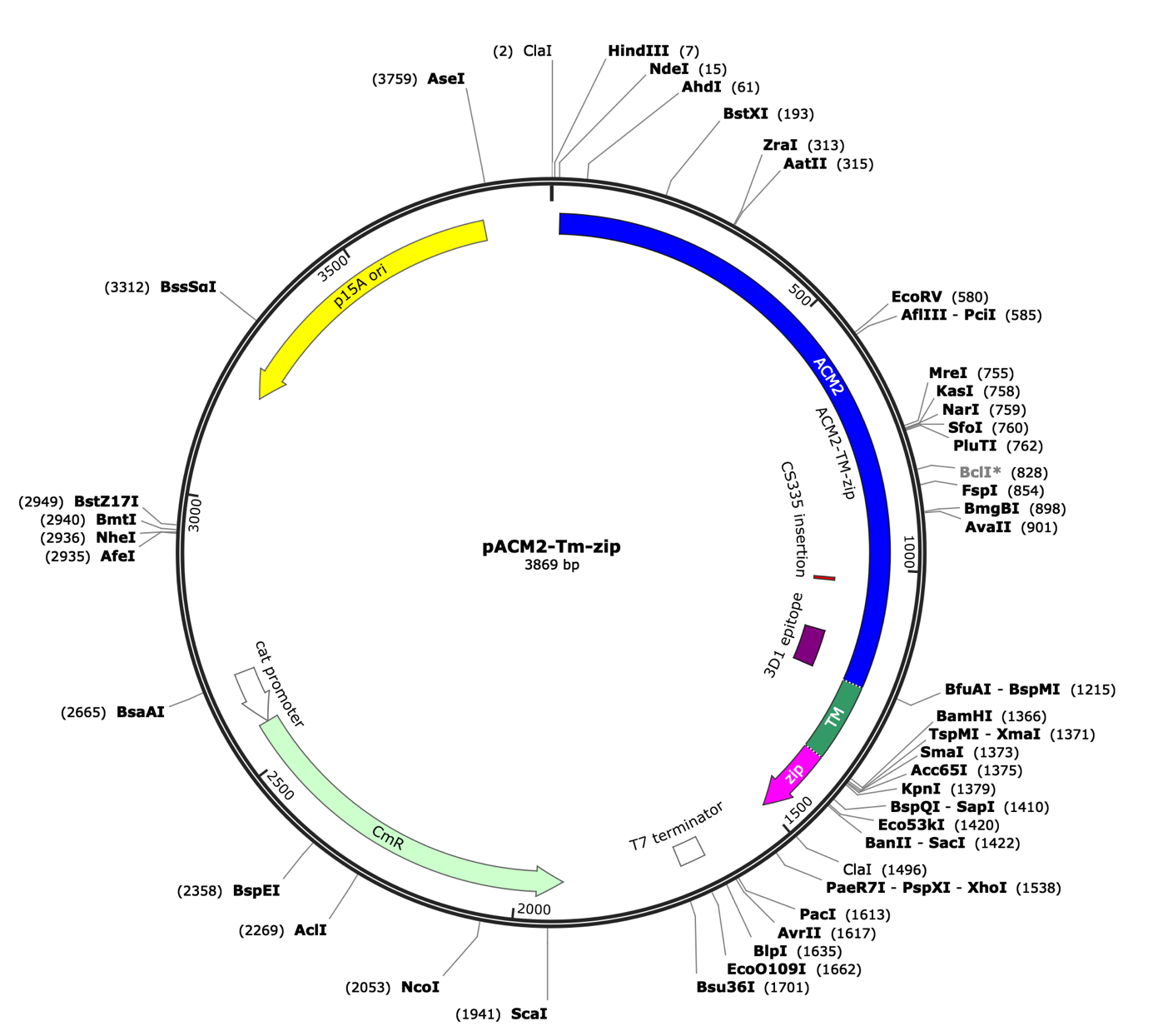
**

**DNA sequence (*Cla*I-*Xho*I fragment)**

**atcgat**aagcttgcatATGCAGCAATCGCATCAGGCTGGTTACGCAAACGCCGCCGACCGGGAGTCTGGCATCCCCGCAGCCGTACTCGATGGCATCAAGGCCGTGGCGAAGGAAAAAAACGCCACATTGATGTTCCGCCTGGTCAACCCCCATTCCACCAGCCTGATTGCCGAAGGGGTGGCCACCAAAGGATTGGGCGTGCACGCCAAGTCGTCCGATTGGGGGTTGCAGGCGGGCTACATTCCCGTCAACCCGAATCTTTCCAAACTGTTCGGCCGTGCGCCCGAGGTGATCGCGCGGGCCGACAACGACGTCAACAGCAGCCTGGCGCATGGCCATACCGCGGTCGACCTGACGCTGTCGAAAGAGCGGCTTGACTATCTGCGGCAAGCGGGCCTGGTCACCGGCATGGCCGATGGCGTGGTCGCGAGCAACCACGCAGGCTACGAGCAGTTCGAGTTTCGCGTGAAGGAAACCTCGGACGGGCGCTATGCCGTGCAGTATCGCCGCAAGGGCGGCGACGATTTCGAGGCGGTCAAGGTGATCGGCAATGCCGCCGGTATTCCACTGACGGCGGATATCGACATGTTCGCCATTATGCCGCATCTGTCCAACTTCCGCGACTCGGCGCGCAGTTCGGTGACCAGCGGCGATTCGGTGACCGATTACCTGGCGCGCACGCGGCGGGCCGCCAGCGAGGCCACGGGCGGCCTGGATCGCGAACGCATCGACTTGTTGTGGAAAATCGCTCGCGCCGGCGCCCGTTCCGCAGTGGGCACCGAGGCGCGTCGCCAGTTCCGCTACGACGGCGACATGAATATCGGCGTGATCACCGATTTCGAGCTGGAAGTGCGCAATGCGCTGAACAGGCGGGCGCACGCCGTCGGCGCGCAGGACGTGGTCCAGCATGGCACTGAGCAGAACAATCCTTTCCCGGAGGCAGATGAGAAGATTTTCGTCGTATCGGCCACCGGTGAAAGCCAGATGCTCACGCGCGGGCAACTGAAGGAATACATTGGC**TGCAGC**CAGCAGCGCGGCGAGGGCTATGTCTTCTACGAGAACCGTGCATACGGCGTGGCGGGGAAAAGCCTGTTCGACGATGGGCTGGGAGCCGCGCCCGGCGTGCCGAGCGGACGTTCGAAGTTCTCGCCGGATGTACTGGAAACGGTGCCGGCGTCACCCGGATTGCGGCGGCCGTCGCTGGGCGCAGTGGAACGCCACTGCAGGTCGACTCTAGAGGATCTAAAATTTATTCTACGTCGCTGTCTGGAAGCGATTCCGACGCTATTTATTCTTATTACTATTTCGTTCTTTATGATGCGCCTCGCGCCGGGAAGCCCTTTTACCGGCGAACGTACTTTAGCGGATCCCCGGGTACCTATCCAGCGTATGAAACAGCTGGAAGACAAAGTTGAAGAGCTCCTGAGCAAAAACTACCACCTGGAGAACGAAGTTGCGCGCCTGAAAAAACTGGTGGGTGAACGTGGGAATTCATCGATATAActaagtaatatggtgcactctcagtacaatctg**ctcgag**

**pACM2-Barnase ( 3960 bp):**

**
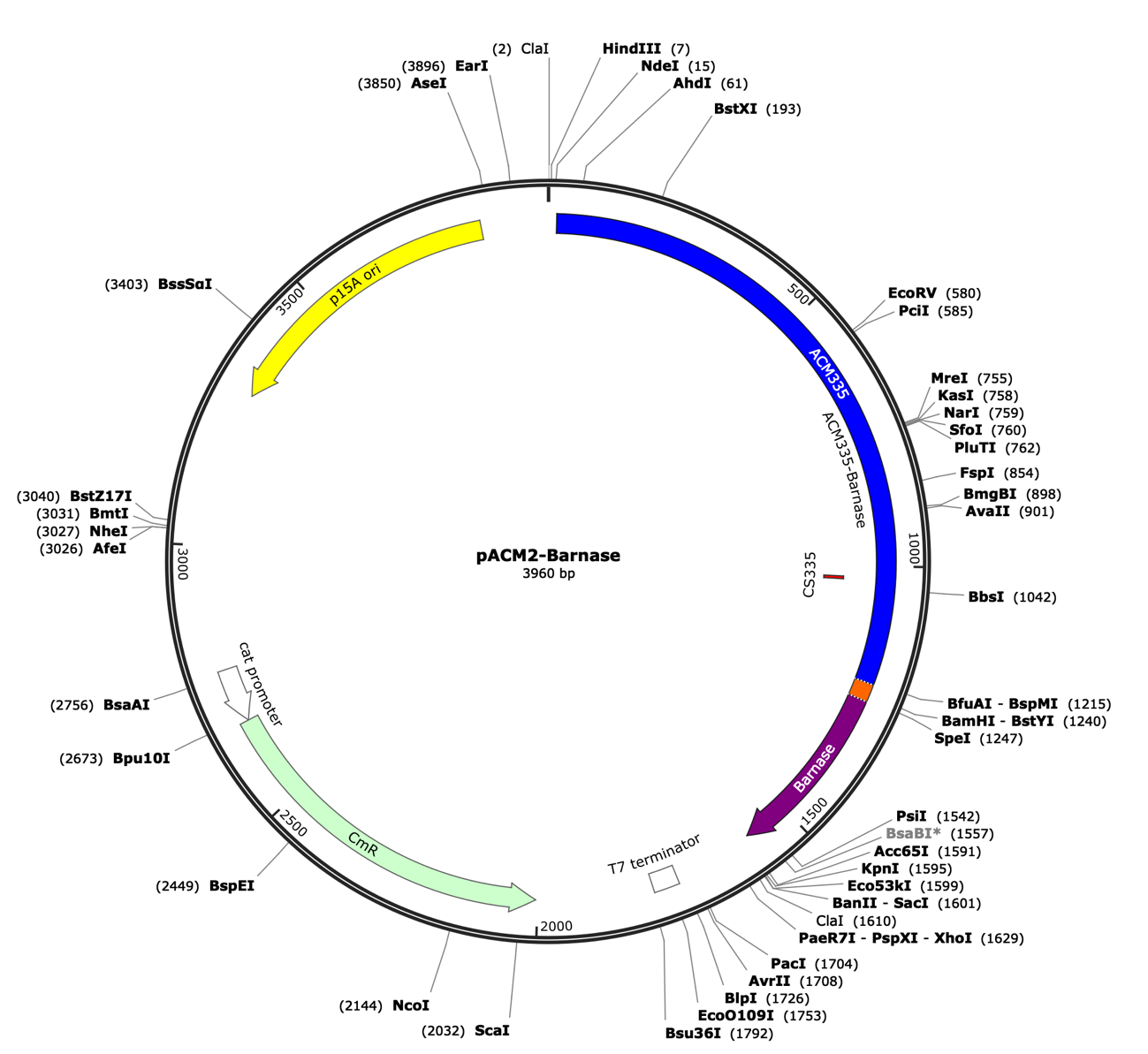
**

**DNA sequence (*Cla*I-*Xho*I fragment)**

**atcgat**aagcttgcatATGCAGCAATCGCATCAGGCTGGTTACGCAAACGCCGCCGACCGGGAGTCTGGCATCCCCGCAGCCGTACTCGATGGCATCAAGGCCGTGGCGAAGGAAAAAAACGCCACATTGATGTTCCGCCTGGTCAACCCCCATTCCACCAGCCTGATTGCCGAAGGGGTGGCCACCAAAGGATTGGGCGTGCACGCCAAGTCGTCCGATTGGGGGTTGCAGGCGGGCTACATTCCCGTCAACCCGAATCTTTCCAAACTGTTCGGCCGTGCGCCCGAGGTGATCGCGCGGGCCGACAACGACGTCAACAGCAGCCTGGCGCATGGCCATACCGCGGTCGACCTGACGCTGTCGAAAGAGCGGCTTGACTATCTGCGGCAAGCGGGCCTGGTCACCGGCATGGCCGATGGCGTGGTCGCGAGCAACCACGCAGGCTACGAGCAGTTCGAGTTTCGCGTGAAGGAAACCTCGGACGGGCGCTATGCCGTGCAGTATCGCCGCAAGGGCGGCGACGATTTCGAGGCGGTCAAGGTGATCGGCAATGCCGCCGGTATTCCACTGACGGCGGATATCGACATGTTCGCCATTATGCCGCATCTGTCCAACTTCCGCGACTCGGCGCGCAGTTCGGTGACCAGCGGCGATTCGGTGACCGATTACCTGGCGCGCACGCGGCGGGCCGCCAGCGAGGCCACGGGCGGCCTGGATCGCGAACGCATCGACTTGTTGTGGAAAATCGCTCGCGCCGGCGCCCGTTCCGCAGTGGGCACCGAGGCGCGTCGCCAGTTCCGCTACGACGGCGACATGAATATCGGCGTGATCACCGATTTCGAGCTGGAAGTGCGCAATGCGCTGAACAGGCGGGCGCACGCCGTCGGCGCGCAGGACGTGGTCCAGCATGGCACTGAGCAGAACAATCCTTTCCCGGAGGCAGATGAGAAGATTTTCGTCGTATCGGCCACCGGTGAAAGCCAGATGCTCACGCGCGGGCAACTGAAGGAATACATTGGC**TGCAGC**CAGCAGCGCGGCGAGGGCTATGTCTTCTACGAGAACCGTGCATACGGCGTGGCGGGGAAAAGCCTGTTCGACGATGGGCTGGGAGCCGCGCCCGGCGTGCCGAGCGGACGTTCGAAGTTCTCGCCGGATGTACTGGAAACGGTGCCGGCGTCACCCGGATTGCGGCGGCCGTCGCTGGGCGCAGTGGAACGCCACTGCAGGTCGACTCTAGAGGATCCAACTAGTGCACAGGTTATCAACACGTTTGACGGGGTTGCGGATTATCTTCAGACATATCATAAGCTACCTGATAATTACATTACAAAATCAGAAGCACAAGCCCTCGGCTGGGTGGCATCAAAAGGGAACCTTGCAGACGTCGCTCCGGGGAAAAGCATCGGCGGAGACATCTTCTCAAACAGGGAAGGCAAACTCCCGGGCAAAAGCGGACGAACATGGCGTGAAGCGGATATTAACTATACATCAGGCTTCAGAAATTCAGACCGGATTCTTTACTCAAGCGACTGGCTGATTTATAAAACAACTGATCATTATCAAACGTTTACAAAAATCAGATAAccccgggtaccgagctcgaattcatcgatataactaagtaata**ctcgag**

**pTrACM2-Gfp ( 5264 bp):**

**Map**

**
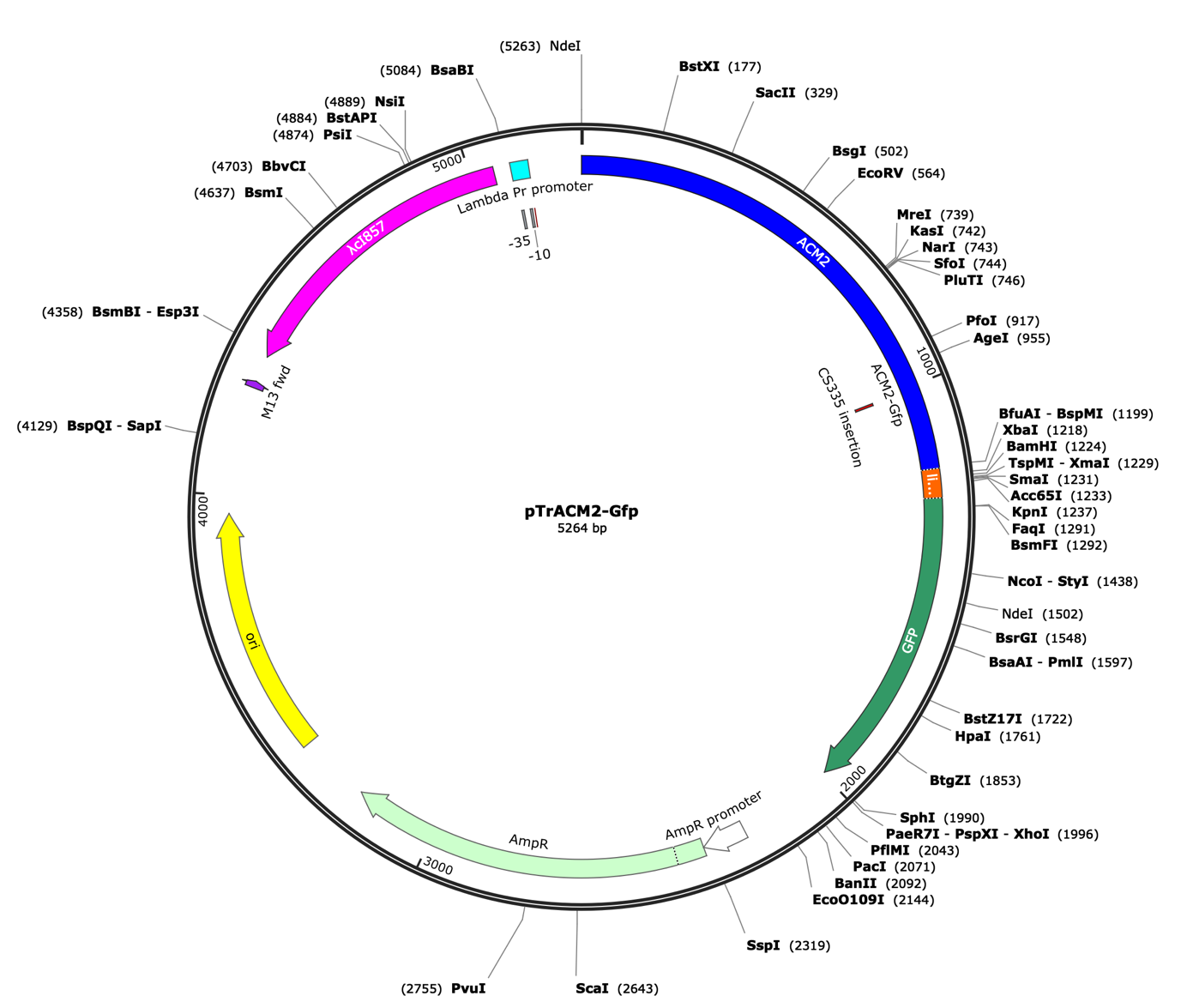
**

**DNA sequence**

ATGCAGCAATCGCATCAGGCTGGTTACGCAAACGCCGCCGACCGGGAGTCTGGCATCCCCGCAGCCGTACTCGATGGCATCAAGGCCGTGGCGAAGGAAAAAAACGCCACATTGATGTTCCGCCTGGTCAACCCCCATTCCACCAGCCTGATTGCCGAAGGGGTGGCCACCAAAGGATTGGGCGTGCACGCCAAGTCGTCCGATTGGGGGTTGCAGGCGGGCTACATTCCCGTCAACCCGAATCTTTCCAAACTGTTCGGCCGTGCGCCCGAGGTGATCGCGCGGGCCGACAACGACGTCAACAGCAGCCTGGCGCATGGCCATACCGCGGTCGACCTGACGCTGTCGAAAGAGCGGCTTGACTATCTGCGGCAAGCGGGCCTGGTCACCGGCATGGCCGATGGCGTGGTCGCGAGCAACCACGCAGGCTACGAGCAGTTCGAGTTTCGCGTGAAGGAAACCTCGGACGGGCGCTATGCCGTGCAGTATCGCCGCAAGGGCGGCGACGATTTCGAGGCGGTCAAGGTGATCGGCAATGCCGCCGGTATTCCACTGACGGCGGATATCGACATGTTCGCCATTATGCCGCATCTGTCCAACTTCCGCGACTCGGCGCGCAGTTCGGTGACCAGCGGCGATTCGGTGACCGATTACCTGGCGCGCACGCGGCGGGCCGCCAGCGAGGCCACGGGCGGCCTGGATCGCGAACGCATCGACTTGTTGTGGAAAATCGCTCGCGCCGGCGCCCGTTCCGCAGTGGGCACCGAGGCGCGTCGCCAGTTCCGCTACGACGGCGACATGAATATCGGCGTGATCACCGATTTCGAGCTGGAAGTGCGCAATGCGCTGAACAGGCGGGCGCACGCCGTCGGCGCGCAGGACGTGGTCCAGCATGGCACTGAGCAGAACAATCCTTTCCCGGAGGCAGATGAGAAGATTTTCGTCGTATCGGCCACCGGTGAAAGCCAGATGCTCACGCGCGGGCAACTGAAGGAATACATTGGC**TGCAGC**CAGCAGCGCGGCGAGGGCTATGTCTTCTACGAGAACCGTGCATACGGCGTGGCGGGGAAAAGCCTGTTCGACGATGGGCTGGGAGCCGCGCCCGGCGTGCCGAGCGGACGTTCGAAGTTCTCGCCGGATGTACTGGAAACGGTGCCGGCGTCACCCGGATTGCGGCGGCCGTCGCTGGGCGCAGTGGAACGCCACTGCAGGTCGACTCTAGAGGATCCCCGGGTACCGGGGGGGTCTATGACCATGATTACGCCAAGCTTGATGAGTAAAGGAGAAGAACTTTTCACTGGAGTTGTCCCAATTCTTGTTGAATTAGATGGTGATGTTAATGGGCACAAATTTTCTGTCAGTGGAGAGGGTGAAGGTGATGCAACATACGGAAAACTTACCCTTAAATTTATTTGCACTACTGGAAAACTACCTGTTCCATGGCCAACACTTGTCACTACTTTCGCGTATGGTCTTCAATGCTTTGCGAGATACCCAGATCATATGAAACAGCATGACTTTTTCAAGAGTGCCATGCCCGAAGGTTATGTACAGGAAAGAACTATATTTTTCAAAGATGACGGGAACTACAAGACACGTGCTGAAGTCAAGTTTGAAGGTGATACCCTTGTTAATAGAATCGAGTTAAAAGGTATTGATTTTAAAGAAGATGGAAACATTCTTGGACACAAATTGGAATACAACTATAACTCACACAATGTATACATCATGGCAGACAAACAAAAGAATGGAATCAAAGTTAACTTCAAAATTAGACACAACATTGAAGATGGAAGCGTTCAACTAGCAGACCATTATCAACAAAATACTCCAATTGGCGATGGCCCTGTCCTTTTACCAGACAACCATTACCTGTCCACACAATCTGCCCTTTCGAAAGATCCCAACGAAAAGAGAGACCACATGGTCCTTCTTGAGTTTGTAACAGCTGCTGGGATTACACATGGCATGGATGAACTATACAAGCATGCGTGActcgagtctggtaaagaaaccgctgctgcgaaatttgaacgccagcacatggactcgtctactagcgcagcttaattaacctagagtcgaccgagcccgcctaatgagcgggcttttttttcatgcaagctaattcttgaagacgaaagggcctcgtgatacgcctatttttataggttaatgtcatgataataatggtttcttagacgtcaggtggcacttttcggggaaatgtgcgcggaacccctatttgtttatttttctaaatacattcaaatatgtatccgctcatgagacaataaccctgataaatgcttcaataatattgaaaaaggaagagtatgagtattcaacatttccgtgtcgcccttattcccttttttgcggcattttgccttcctgtttttgctcacccagaaacgctggtgaaagtaaaagatgctgaagatcagttgggtgcacgagtgggttacatcgaactggatctcaacagcggtaagatccttgagagttttcgccccgaagaacgttttccaatgatgagcacttttaaagttctgctatgtggcgcggtattatcccgtattgacgtcgggcaagagcaactcggtcgccgcatacactattctcagaatgacttggttgagtactcaccagtcacagaaaagcatcttacggatggcatgacagtaagagaattatgcagtgctgccataaccatgagtgataacactgcggccaacttacttctgacaacgatcggaggaccgaaggagctaaccgcttttttgcacaacatgggggatcaagtaactcgccttgatcgttgggaaccggagctgaatgaagccataccaaacgacgagcgtgacaccacgatgcctgtagcaatggcaacaacgttgcgcaaactattaactggcgaactacttactctagcttcccggcaacaattaatagactggatggaggcggataaagttgcaggaccacttctgcgctcggcccttccggctggctggtttattgctgataaatctggagccggtgagcgtgggtctcgcggtatcattgcagcactggggccagatggtaagccctcccgtatcgtagttatctacacgacggggagtcaggcaactatggatgaacgaaatagacagatcgctgagataggtgcctcactgattaagcattggtaactgtcagaccaagtttactcatatatactttagattgatttaaaacttcatttttaatttaaaaggatctaggtgaagatcctttttgataatctcatgaccaaaatcccttaacgtgagttttcgttccactgagcgtcagaccccgtagaaaagatcaaaggatcttcttgagatcctttttttctgcgcgtaatctgctgcttgcaaacaaaaaaaccaccgctaccagcggtggtttgtttgccggatcaagagctaccaactctttttccgaaggtaactggcttcagcagagcgcagataccaaatactgtccttctagtgtagccgtagttaggccaccacttcaagaactctgtagcaccgcctacatacctcgctctgctaatcctgttaccagtggctgctgccagtggcgataagtcgtgtcttaccgggttggactcaagacgatagttaccggataaggcgcagcggtcgggctgaacggggggttcgtgcacacagcccagcttggagcgaacgacctacaccgaactgagatacctacagcgtgagcattgagaaagcgccacgcttcccgaagggagaaaggcggacaggtatccggtaagcggcagggtcggaacaggagagcgcacgagggagcttccagggggaaacgcctggtatctttatagtcctgtcgggtttcgccacctctgacttgagcgtcgatttttgtgatgctcgtcaggggggcggagcctatggaaaaacgccagcaacgcggcctttttacggttcctggccttttgctggccttttgctcacatgttctctcctgcgttatcccctgattctgtggataaccgtattaccgcctttgagtgagctgataccgctcgccgcagccgaacgaccgagcgcagcgagtcagtgagcgaggaagcggaagagcgcccaatacgcaaaccgcctctccccgcgcgttggccgattcattaatgcagctggcgaaagggggatgtgctgcaaggcgattaagttgggtaacgccagggttttcccagtcacgacgttgtaaaacgacggccagtgccaagcttgaagattcttgctcaattgttatcagctatgcgccgaccagaacaccttgccgatcagccaaacgtctcttcaggccactgactagcgataactttccccacaacggaacaactctcattgcatgggatcattgggtactgtgggtttagtggttgtaaaaacacctgaccgctatccctgatcagtttcttgaaggtaaactcatcacccccaagtctggctatgcagaaatcacctggctcaacagcctgctcagggtcaacgagaattaacattccgtcaggaaagcttggcttggagcctgttggtgcggtcatggaattaccttcaacctcaagccagaatgcagaatcactggcttttttggttgtgcttacccatctctccgcatcacctttggtaaaggttctaagctgaggtgagaacatccctgcctgaacatgagaaaaaacagggtactcatactcacttctaagtgacggctgcatactaaccgcttcatacatctcgtagatttctctggcgattgaagggctaaattcttcaacgctaactttgagaatttttgtaagcaatgcggcgttataagcatttaatgcattgatgccattaaataaagcaccaacgcctgactgccccatccccatcttgtctacgacagattcctgggataagccaagttcatttttctttttttcataaattgctttaaggcgacgtgcgtcctcaagctgctcttgtgttaatggtttcttttttgtgctcatacgttaaatctatcaccgcaagggataaatatctaacaccgtgcgtgttgactattttacctctggcggtgataatggttgcatgtactaaggaggttgtatggaacaacgcataaccctgaaagattatgcaatgcgctttgggcaaaccaagacagctaaagatcctagaaataattttgtttaactttaagaaggagatatacat

**pTCam ( 4144 bp)**

**Map**

**
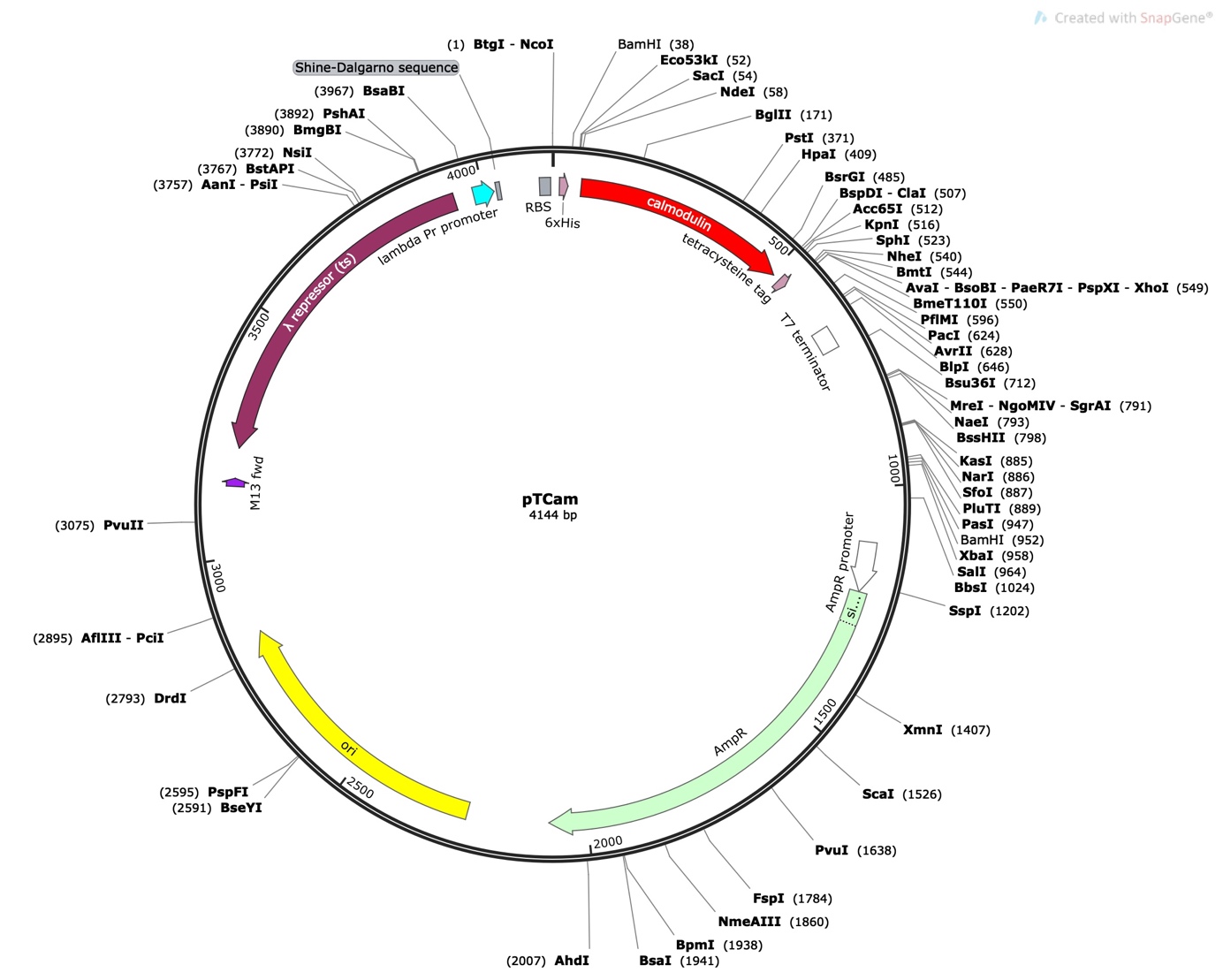
**

**Full DNA sequence**

ccATGGGCAGCAGC**CATCACCATCATCACCAC**AGCCAGGATCCGAATTCGAGCTCCCATATGGCTGACCAACTGACAGAAGAGCAGATTGCAGAATTCAAAGAAGCTTTTTCACTATTTGACAAAGATGGTGATGGAACTATAACAACAAAGGAATTGGGAACTGTAATGAGATCTCTTGGGCAGAATCCCACAGAAGCAGAGTTACAGGACATGATTAATGAAGTAGATGCTGATGGTAATGGCACAATTGACTTCCCTGAATTTCTGACAATGATGGCAAGAAAAATGAAAGACACAGACAGTGAAGAAGAAATTAGAGAAGCATTCCGTGTGTTTGATAAGGATGGCAATGGCTATATTAGTGCTGCAGAACTTCGCCATGTGATGACAAACCTTGGAGAGAAGTTAACAGATGAAGAAGTTGATGAAATGATCAGGGAAGCAGATATTGATGGTGATGGTCAAGTAAACTATGAAGAGTTTGTACAAATGATGACAGCGAAATCGATGGTACCCGCA**TGCTGT**CCGGGT**TGCTGT**GCTAGCTAActcgagtctggtaaagaaaccgctgctgcgaaatttgaacgccagcacatggactcgtctactagcgcagcttaattaacctaggctgctgccaccgctgagcaataactagcataaccccttggggcctctaaacgggtcttgaggggttttttgctgaaacctcaggcatttgagaagcacacggtcacactgcttccggtagtcaataaaccggaccccggcgcggcggcggctgccccgccggcggcgcgcgtgccggacacgctgatgcagtccctggctgtcaactggcgctgaagcgccgtgaatcacggcccgcctgcctcgcgcggcggcgccgtctctttgcgttcttctccgaggtatttcccatcatgaattcttttgctttttaccctggggatcctctagagtcgaccgagcccgcctaatgagcgggcttttttttcatgcaagctaattcttgaagacgaaagggcctcgtgatacgcctatttttataggttaatgtcatgataataatggtttcttagacgtcaggtggcacttttcggggaaatgtgcgcggaacccctatttgtttatttttctaaatacattcaaatatgtatccgctcatgagacaataaccctgataaatgcttcaataatattgaaaaaggaagagtatgagtattcaacatttccgtgtcgcccttattcccttttttgcggcattttgccttcctgtttttgctcacccagaaacgctggtgaaagtaaaagatgctgaagatcagttgggtgcacgagtgggttacatcgaactggatctcaacagcggtaagatccttgagagttttcgccccgaagaacgttttccaatgatgagcacttttaaagttctgctatgtggcgcggtattatcccgtattgacgtcgggcaagagcaactcggtcgccgcatacactattctcagaatgacttggttgagtactcaccagtcacagaaaagcatcttacggatggcatgacagtaagagaattatgcagtgctgccataaccatgagtgataacactgcggccaacttacttctgacaacgatcggaggaccgaaggagctaaccgcttttttgcacaacatgggggatcaagtaactcgccttgatcgttgggaaccggagctgaatgaagccataccaaacgacgagcgtgacaccacgatgcctgtagcaatggcaacaacgttgcgcaaactattaactggcgaactacttactctagcttcccggcaacaattaatagactggatggaggcggataaagttgcaggaccacttctgcgctcggcccttccggctggctggtttattgctgataaatctggagccggtgagcgtgggtctcgcggtatcattgcagcactggggccagatggtaagccctcccgtatcgtagttatctacacgacggggagtcaggcaactatggatgaacgaaatagacagatcgctgagataggtgcctcactgattaagcattggtaactgtcagaccaagtttactcatatatactttagattgatttaaaacttcatttttaatttaaaaggatctaggtgaagatcctttttgataatctcatgaccaaaatcccttaacgtgagttttcgttccactgagcgtcagaccccgtagaaaagatcaaaggatcttcttgagatcctttttttctgcgcgtaatctgctgcttgcaaacaaaaaaaccaccgctaccagcggtggtttgtttgccggatcaagagctaccaactctttttccgaaggtaactggcttcagcagagcgcagataccaaatactgtccttctagtgtagccgtagttaggccaccacttcaagaactctgtagcaccgcctacatacctcgctctgctaatcctgttaccagtggctgctgccagtggcgataagtcgtgtcttaccgggttggactcaagacgatagttaccggataaggcgcagcggtcgggctgaacggggggttcgtgcacacagcccagcttggagcgaacgacctacaccgaactgagatacctacagcgtgagcattgagaaagcgccacgcttcccgaagggagaaaggcggacaggtatccggtaagcggcagggtcggaacaggagagcgcacgagggagcttccagggggaaacgcctggtatctttatagtcctgtcgggtttcgccacctctgacttgagcgtcgatttttgtgatgctcgtcaggggggcggagcctatggaaaaacgccagcaacgcggcctttttacggttcctggccttttgctggccttttgctcacatgttctctcctgcgttatcccctgattctgtggataaccgtattaccgcctttgagtgagctgataccgctcgccgcagccgaacgaccgagcgcagcgagtcagtgagcgaggaagcggaagagcgcccaatacgcaaaccgcctctccccgcgcgttggccgattcattaatgcagctggcgaaagggggatgtgctgcaaggcgattaagttgggtaacgccagggttttcccagtcacgacgttgtaaaacgacggccagtgccaagcttgaagattcttgctcaattgttatcagctatgcgccgaccagaacaccttgccgatcagccaaacgtctcttcaggccactgactagcgataactttccccacaacggaacaactctcattgcatgggatcattgggtactgtgggtttagtggttgtaaaaacacctgaccgctatccctgatcagtttcttgaaggtaaactcatcacccccaagtctggctatgcagaaatcacctggctcaacagcctgctcagggtcaacgagaattaacattccgtcaggaaagcttggcttggagcctgttggtgcggtcatggaattaccttcaacctcaagccagaatgcagaatcactggcttttttggttgtgcttacccatctctccgcatcacctttggtaaaggttctaagctcaggtgagaacatccctgcctgaacatgagaaaaaacagggtactcatactcacttctaagtgacggctgcatactaaccgcttcatacatctcgtagatttctctggcgattgaagggctaaattcttcaacgctaactttgagaatttttgtaagcaatgcggcgttataagcatttaatgcattgatgccattaaataaagcaccaacgcctgactgccccatccccatcttgtctacgacagattcctgggataagccaagttcatttttctttttttcataaattgctttaaggcgacgtgcgtcctcaagctgctcttgtgttaatggtttcttttttgtgctcatacgttaaatctatcaccgcaagggataaatatctaacaccgtgcgtgttgactattttacctctggcggtgataatggttgcatgtactaaggaggttgtatggaacaacgcataaccctgaaagattatgcaatgcgctttgggcaaaccaagacagctaaagatcctagaaataattttgtttaactttaagaaggagatata

**pTCam-V_9A_ ( 4544 bp)**

**Map**

**
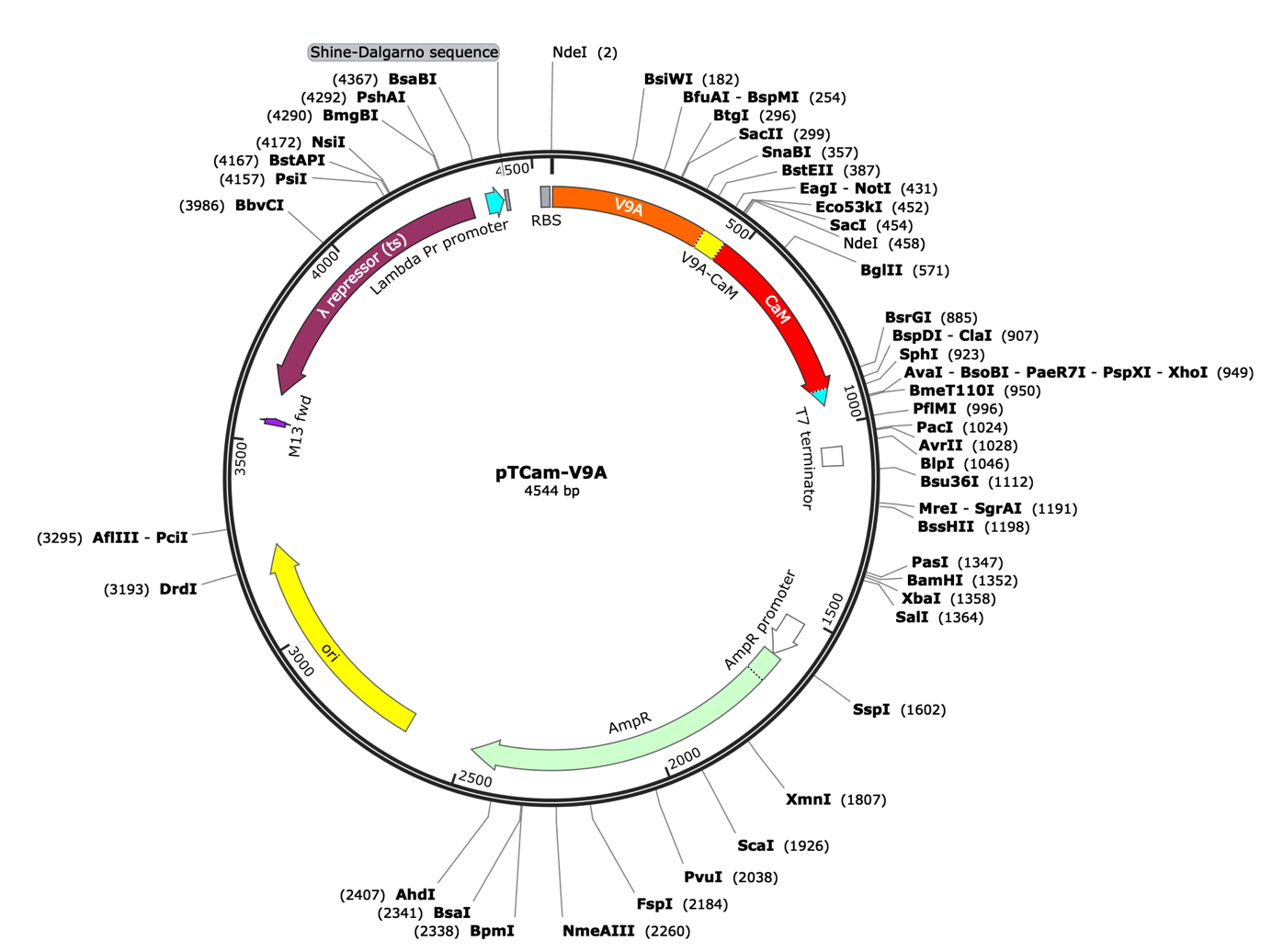
**

**Full DNA sequence**

**catATG**GCTGACGTTCAGCTGCAGGAATCTGGTGGTGGTTCTGTTCAGGCGGGTGGTTCTCTGCGTCTGTCTTGCGCGGCTAGCGGTGACACCTTCTCTTCTTACTCTATGGCGTGGTTCCGTCAGGCGCCGGGTAAAGAATGCGAACTGGTTTCTAACATCCTGCGTGACGGTACTACCACGTACGCCGGCTCTGTTAAAGGTCGTTTCACCATCTCTCGTGACGACGCGAAAAACACCGTTTACCTGCAGATGGTTAACCTGAAATCTGAAGACACCGCGCGTTACTACTGCGCCGCGGACTCTGGTACTCAGCTGGGTTACGTTGGTGCGGTTGGTCTGTCTTGCCTGGACTACGTAATGGACTACTGGGGTAAAGGTACTCAGGTTACCGTTTCTTCTGAACCGAAAACCCCGAAACCGCAGCCAGCGGCCGCTGAAAAGGTACCGAGCTCCCATATGGCTGACCAACTGACAGAAGAGCAGATTGCAGAATTCAAAGAAGCTTTTTCACTATTTGACAAAGATGGTGATGGAACTATAACAACAAAGGAATTGGGAACTGTAATGAGATCTCTTGGGCAGAATCCCACAGAAGCAGAGTTACAGGACATGATTAATGAAGTAGATGCTGATGGTAATGGCACAATTGACTTCCCTGAATTTCTGACAATGATGGCAAGAAAAATGAAAGACACAGACAGTGAAGAAGAAATTAGAGAAGCATTCCGTGTGTTTGATAAGGATGGCAATGGCTATATTAGTGCTGCAGAACTTCGCCATGTGATGACAAACCTTGGAGAGAAGTTAACAGATGAAGAAGTTGATGAAATGATCAGGGAAGCAGATATTGATGGTGATGGTCAAGTAAACTATGAAGAGTTTGTACAAATGATGACAGCGAAATCGATGGTACCCGCATGCTGTCCGGGTTGCTGTGCTAGCTAA**ctcgag**tctggtaaagaaaccgctgctgcgaaatttgaacgccagcacatggactcgtctactagcgcagcttaattaacctaggctgctgccaccgctgagcaataactagcataaccccttggggcctctaaacgggtcttgaggggttttttgctgaaacctcaggcatttgagaagcacacggtcacactgcttccggtagtcaataaaccggaccccggcgcggcggcggctgccccgccggcggcgcgcgtgccggacacgctgatgcagtccctggctgtcaactggcgctgaagcgccgtgaatcacggcccgcctgcctcgcgcggcggcgccgtctctttgcgttcttctccgaggtatttcccatcatgaattcttttgctttttaccctggggatcctctagagtcgaccgagcccgcctaatgagcgggcttttttttcatgcaagctaattcttgaagacgaaagggcctcgtgatacgcctatttttataggttaatgtcatgataataatggtttcttagacgtcaggtggcacttttcggggaaatgtgcgcggaacccctatttgtttatttttctaaatacattcaaatatgtatccgctcatgagacaataaccctgataaatgcttcaataatattgaaaaaggaagagtatgagtattcaacatttccgtgtcgcccttattcccttttttgcggcattttgccttcctgtttttgctcacccagaaacgctggtgaaagtaaaagatgctgaagatcagttgggtgcacgagtgggttacatcgaactggatctcaacagcggtaagatccttgagagttttcgccccgaagaacgttttccaatgatgagcacttttaaagttctgctatgtggcgcggtattatcccgtattgacgtcgggcaagagcaactcggtcgccgcatacactattctcagaatgacttggttgagtactcaccagtcacagaaaagcatcttacggatggcatgacagtaagagaattatgcagtgctgccataaccatgagtgataacactgcggccaacttacttctgacaacgatcggaggaccgaaggagctaaccgcttttttgcacaacatgggggatcaagtaactcgccttgatcgttgggaaccggagctgaatgaagccataccaaacgacgagcgtgacaccacgatgcctgtagcaatggcaacaacgttgcgcaaactattaactggcgaactacttactctagcttcccggcaacaattaatagactggatggaggcggataaagttgcaggaccacttctgcgctcggcccttccggctggctggtttattgctgataaatctggagccggtgagcgtgggtctcgcggtatcattgcagcactggggccagatggtaagccctcccgtatcgtagttatctacacgacggggagtcaggcaactatggatgaacgaaatagacagatcgctgagataggtgcctcactgattaagcattggtaactgtcagaccaagtttactcatatatactttagattgatttaaaacttcatttttaatttaaaaggatctaggtgaagatcctttttgataatctcatgaccaaaatcccttaacgtgagttttcgttccactgagcgtcagaccccgtagaaaagatcaaaggatcttcttgagatcctttttttctgcgcgtaatctgctgcttgcaaacaaaaaaaccaccgctaccagcggtggtttgtttgccggatcaagagctaccaactctttttccgaaggtaactggcttcagcagagcgcagataccaaatactgtccttctagtgtagccgtagttaggccaccacttcaagaactctgtagcaccgcctacatacctcgctctgctaatcctgttaccagtggctgctgccagtggcgataagtcgtgtcttaccgggttggactcaagacgatagttaccggataaggcgcagcggtcgggctgaacggggggttcgtgcacacagcccagcttggagcgaacgacctacaccgaactgagatacctacagcgtgagcattgagaaagcgccacgcttcccgaagggagaaaggcggacaggtatccggtaagcggcagggtcggaacaggagagcgcacgagggagcttccagggggaaacgcctggtatctttatagtcctgtcgggtttcgccacctctgacttgagcgtcgatttttgtgatgctcgtcaggggggcggagcctatggaaaaacgccagcaacgcggcctttttacggttcctggccttttgctggccttttgctcacatgttctctcctgcgttatcccctgattctgtggataaccgtattaccgcctttgagtgagctgataccgctcgccgcagccgaacgaccgagcgcagcgagtcagtgagcgaggaagcggaagagcgcccaatacgcaaaccgcctctccccgcgcgttggccgattcattaatgcagctggcgaaagggggatgtgctgcaaggcgattaagttgggtaacgccagggttttcccagtcacgacgttgtaaaacgacggccagtgccaagcttgaagattcttgctcaattgttatcagctatgcgccgaccagaacaccttgccgatcagccaaacgtctcttcaggccactgactagcgataactttccccacaacggaacaactctcattgcatgggatcattgggtactgtgggtttagtggttgtaaaaacacctgaccgctatccctgatcagtttcttgaaggtaaactcatcacccccaagtctggctatgcagaaatcacctggctcaacagcctgctcagggtcaacgagaattaacattccgtcaggaaagcttggcttggagcctgttggtgcggtcatggaattaccttcaacctcaagccagaatgcagaatcactggcttttttggttgtgcttacccatctctccgcatcacctttggtaaaggttctaagctgaggtgagaacatccctgcctgaacatgagaaaaaacagggtactcatactcacttctaagtgacggctgcatactaaccgcttcatacatctcgtagatttctctggcgattgaagggctaaattcttcaacgctaactttgagaatttttgtaagcaatgcggcgttataagcatttaatgcattgatgccattaaataaagcaccaacgcctgactgccccatccccatcttgtctacgacagattcctgggataagccaagttcatttttctttttttcataaattgctttaaggcgacgtgcgtcctcaagctgctcttgtgttaatggtttcttttttgtgctcatacgttaaatctatcaccgcaagggataaatatctaacaccgtgcgtgttgactattttacctctggcggtgataatggttgcatgtactaaggaggttgtatggaacaacgcataaccctgaaagattatgcaatgcgctttgggcaaaccaagacagctaaagatcctagaaataattttgtttaactttaagaaggagatata

**pTCam-V_9A_-Zip ( 4778 bp)**

**Map**

**
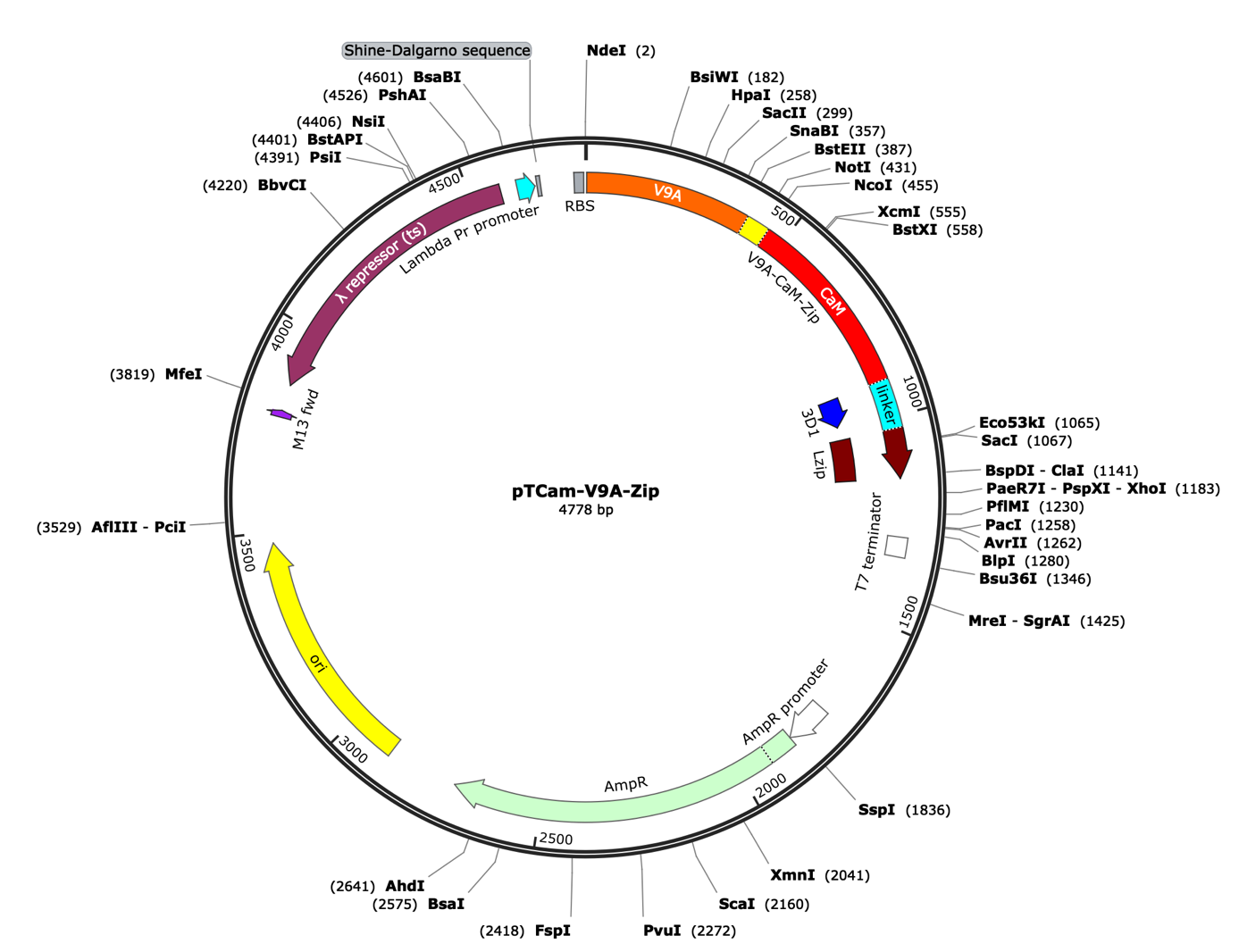
**

**DNA sequence (*Nde*I-*Xho*I fragment)**

**catATG**GCTGACGTTCAGCTGCAGGAATCTGGTGGTGGTTCTGTTCAGGCGGGTGGTTCTCTGCGTCTGTCTTGCGCGGCTAGCGGTGACACCTTCTCTTCTTACTCTATGGCGTGGTTCCGTCAGGCGCCGGGTAAAGAATGCGAACTGGTTTCTAACATCCTGCGTGACGGTACTACCACGTACGCCGGCTCTGTTAAAGGTCGTTTCACCATCTCTCGTGACGACGCGAAAAACACCGTTTACCTGCAGATGGTTAACCTGAAATCTGAAGACACCGCGCGTTACTACTGCGCCGCGGACTCTGGTACTCAGCTGGGTTACGTTGGTGCGGTTGGTCTGTCTTGCCTGGACTACGTAATGGACTACTGGGGTAAAGGTACTCAGGTTACCGTTTCTTCTGAACCGAAAACCCCGAAACCGCAGCCAGCGGCCGCTGAAAAGGTACCCGGGTCCATGGCTAGCGCCGACCAGCTGACCGAGGAGCAGATCGCCGAGTTCAAGGAGGCCTTCTCCCTGTTCGACAAGGACGGCGACGGCACCATCACCACCAAGGAGCTGGGCACCGTCATGCGGTCCCTGGGCCAGAACCCCACCGAGGCCGAGCTTCAGGACATGATCAACGAGGTCGACGCCGACGGCAACGGCACCATCGACTTCCCCGAGTTCCTGACCATGATGGCCCGGAAGATGAAGGACACCGACTCCGAGGAGGAGATCCGGGAGGCCTTCCGGGTCTTCGACAAGGACGGCAACGGCTATATCTCCGCCGCCGAGCTGCGGCACGTCATGACCAACCTGGGCGAGAAGCTGACCGACGAGGAGGTCGACGAGATGATCCGGGAGGCCGACATCGACGGCGACGGCCAGGTCAACTATGAGGAGTTCGTCCAGATGATGACCGCCAAATCGAAGTTCTCGCCGGATGTACTGGAAACGGTGCCGGCGTCACCCGGATTGCGGCGGCCGTCGCTGGGCGCAGTGGAACGCCACTGCAGGTCGACTCTAGAGGATCCCCGGGTACCTATCCAGCGTATGAAACAGCTGGAAGACAAAGTTGAAGAGCTCCTGAGCAAAAACTACCACCTGGAGAACGAAGTTGCGCGCCTGAAAAAACTGGTGGGTGAACGTGGGAATTCATCGATATAActaagtaatatggtgcactctcagtacaatctg**ctcgag**

**pTCam-Frb (4384 bp)**

**Map**

**
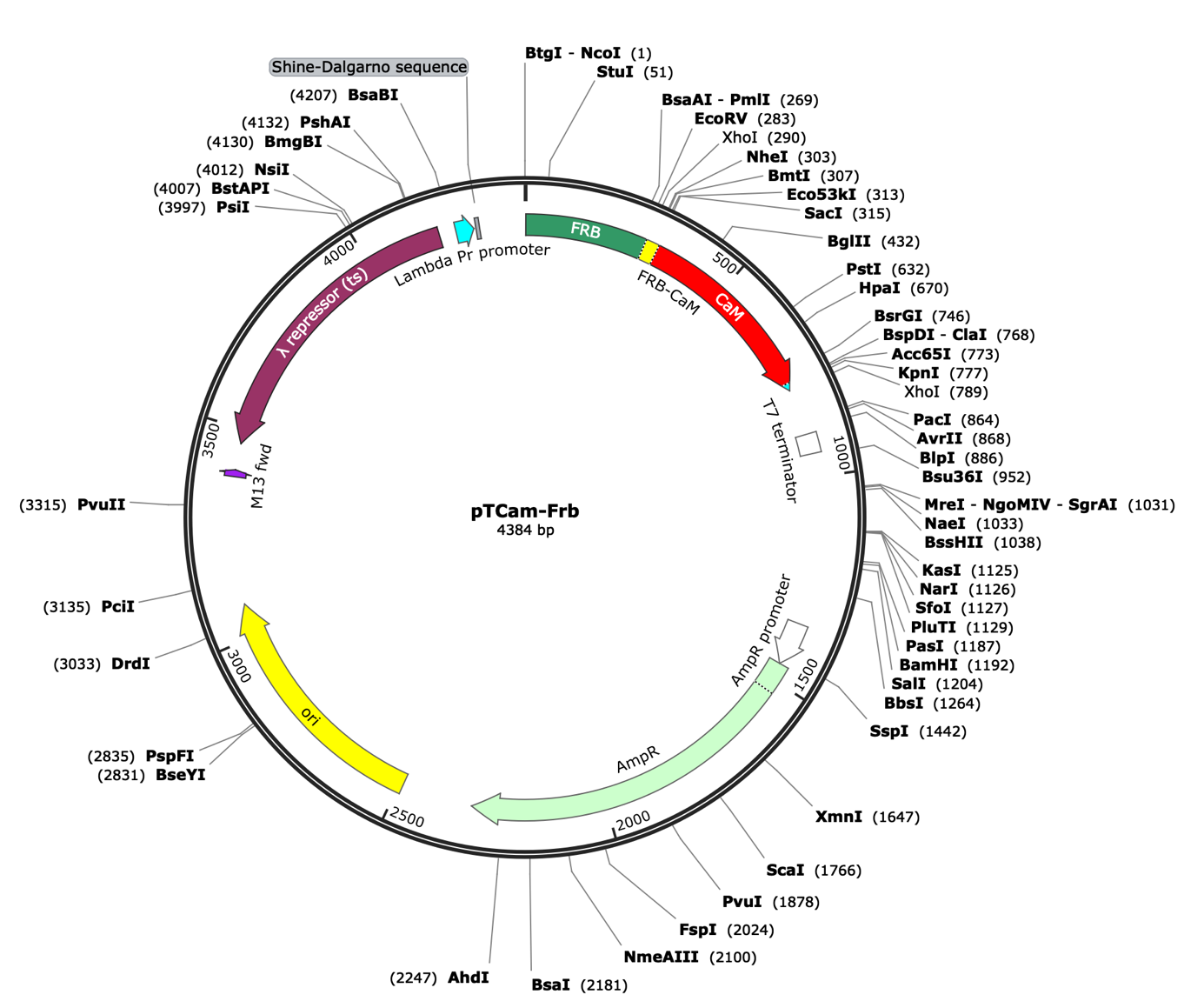
**

**DNA sequence (*Nco*I-*Xho*I fragment)**

ccATGGTAGCCATCCTCTGGCATGAGATGTGGCATGAAGGTCTAGAAGAGGCCTCTCGCTTGTACTTTGGGGAGAGGAACGTCAAAGGCATGTTTGAGGTGCTGGAGCCCCTGCATGCTATGATGGAACGCGGTCCCCAGACCCTGAAGGAAACGTCCTTTAATCAGGCATATGGTCGAGATTTAATGGAGGCACAAGAATGGTGCCGAAAGTACATGAAATCAGGGAACGTCAAGGACCTCACCCAAGCCTGGGACCTCTACTATCACGTGTTCAGACGGATATCAGGCTCGAGTGGCCCGGCTAGCCCGAGCTCCCATATGGCTGACCAACTGACAGAAGAGCAGATTGCAGAATTCAAAGAAGCTTTTTCACTATTTGACAAAGATGGTGATGGAACTATAACAACAAAGGAATTGGGAACTGTAATGAGATCTCTTGGGCAGAATCCCACAGAAGCAGAGTTACAGGACATGATTAATGAAGTAGATGCTGATGGTAATGGCACAATTGACTTCCCTGAATTTCTGACAATGATGGCAAGAAAAATGAAAGACACAGACAGTGAAGAAGAAATTAGAGAAGCATTCCGTGTGTTTGATAAGGATGGCAATGGCTATATTAGTGCTGCAGAACTTCGCCATGTGATGACAAACCTTGGAGAGAAGTTAACAGATGAAGAAGTTGATGAAATGATCAGGGAAGCAGATATTGATGGTGATGGTCAAGTAAACTATGAAGAGTTTGTACAAATGATGACAGCGAAATCGATGGTACCCGCATGCTAActcgag

**pK1Cam-V_9A_  ( 3333 bp)**

**Map**

**
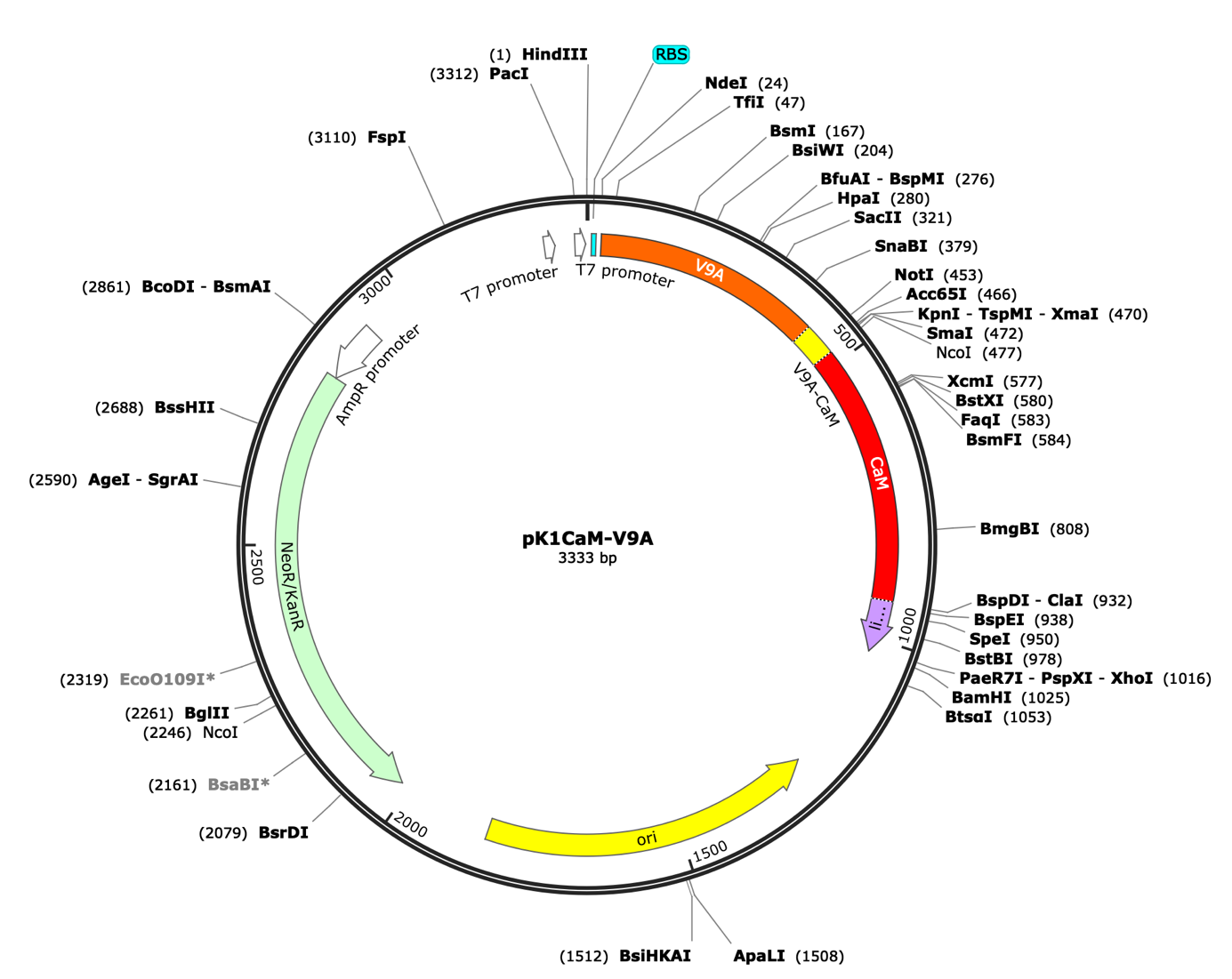
**

**Full DNA sequence**

**aagctt**ataagaaggagatatacatATGGCTGACGTTCAGCTGCAGGAATCTGGTGGTGGTTCTGTTCAGGCGGGTGGTTCTCTGCGTCTGTCTTGCGCGGCTAGCGGTGACACCTTCTCTTCTTACTCTATGGCGTGGTTCCGTCAGGCGCCGGGTAAAGAATGCGAACTGGTTTCTAACATCCTGCGTGACGGTACTACCACGTACGCCGGCTCTGTTAAAGGTCGTTTCACCATCTCTCGTGACGACGCGAAAAACACCGTTTACCTGCAGATGGTTAACCTGAAATCTGAAGACACCGCGCGTTACTACTGCGCCGCGGACTCTGGTACTCAGCTGGGTTACGTTGGTGCGGTTGGTCTGTCTTGCCTGGACTACGTAATGGACTACTGGGGTAAAGGTACTCAGGTTACCGTTTCTTCTGAACCGAAAACCCCGAAACCGCAGCCAGCGGCCGCTGAAAAGGTACCCGGGTCCATGGCTAGCGCCGACCAGCTGACCGAGGAGCAGATCGCCGAGTTCAAGGAGGCCTTCTCCCTGTTCGACAAGGACGGCGACGGCACCATCACCACCAAGGAGCTGGGCACCGTCATGCGGTCCCTGGGCCAGAACCCCACCGAGGCCGAGCTTCAGGACATGATCAACGAGGTCGACGCCGACGGCAACGGCACCATCGACTTCCCCGAGTTCCTGACCATGATGGCCCGGAAGATGAAGGACACCGACTCCGAGGAGGAGATCCGGGAGGCCTTCCGGGTCTTCGACAAGGACGGCAACGGCTATATCTCCGCCGCCGAGCTGCGGCACGTCATGACCAACCTGGGCGAGAAGCTGACCGACGAGGAGGTCGACGAGATGATCCGGGAGGCCGACATCGACGGCGACGGCCAGGTCAACTATGAGGAGTTCGTCCAGATGATGACCGCCAAATCGATGTCCGGAGGTGGCACTAGTGCTTCAGGTCTGAACGACATCTTCGAAGCTCAGAAAATCGAATGGCACGAAGGCGGCACCCTCGAGTAA**ggatcc**ctgggcctcatgggccttcctttcactgcccgctttccagtcgggaaacctgtcgtgccagctgcattaacatggtcatagctgtttccttgcgtattgggcgctctccgcttcctcgctcactgactcgctgcgctcggtcgttcgggtaaagcctggggtgcctaatgagcaaaaggccagcaaaaggccaggaaccgtaaaaaggccgcgttgctggcgtttttccataggctccgcccccctgacgagcatcacaaaaatcgacgctcaagtcagaggtggcgaaacccgacaggactataaagataccaggcgtttccccctggaagctccctcgtgcgctctcctgttccgaccctgccgcttaccggatacctgtccgcctttctcccttcgggaagcgtggcgctttctcatagctcacgctgtaggtatctcagttcggtgtaggtcgttcgctccaagctgggctgtgtgcacgaaccccccgttcagcccgaccgctgcgccttatccggtaactatcgtcttgagtccaacccggtaagacacgacttatcgccactggcagcagccactggtaacaggattagcagagcgaggtatgtaggcggtgctacagagttcttgaagtggtggcctaactacggctacactagaagaacagtatttggtatctgcgctctgctgaagccagttaccttcggaaaaagagttggtagctcttgatccggcaaacaaaccaccgctggtagcggtggtttttttgtttgcaagcagcagattacgcgcagaaaaaaaggatctcaagaagatcctttgatcttttctacggggtctgacgctcagtggaacgaaaactcacgttaagggattttggtcatgagattatcaaaaaggatcttcacctagatccttttaaattaaaaatgaagttttaaatcaatctaaagtatatatgagtaaacttggtctgacagttattagaaaaattcatccagcagacgataaaacgcaatacgctggctatccggtgccgcaatgccatacagcaccagaaaacgatccgcccattcgccgcccagttcttccgcaatatcacgggtggccagcgcaatatcctgataacgatccgccacgcccagacggccgcaatcaataaagccgctaaaacggccattttccaccataatgttcggcaggcacgcatcaccatgggtcaccaccagatcttcgccatccggcatgctcgctttcagacgcgcaaacagctctgccggtgccaggccctgatgttcttcatccagatcatcctgatccaccaggcccgcttccatacgggtacgcgcacgttcaatacgatgtttcgcctgatgatcaaacggacaggtcgccgggtccagggtatgcagacgacgcatggcatccgccataatgctcactttttctgccggcgccagatggctagacagcagatcctgacccggcacttcgcccagcagcagccaatcacggcccgcttcggtcaccacatccagcaccgccgcacacggaacaccggtggtggccagccagctcagacgcgccgcttcatcctgcagctcgttcagcgcaccgctcagatcggttttcacaaacagcaccggacgaccctgcgcgctcagacgaaacaccgccgcatcagagcagccaatggtctgctgcgcccaatcatagccaaacagacgttccacccacgctgccgggctacccgcatgcaggccatcctgttcaatcatactcttcctttttcaatattattgaagcatttatcagggttattgtctcatgagcggatacatatttgaatgtatttagaaaaataaacaaataggggttccgcgcacatttccccgaaaagtgccacctaaattgtaagcgttaatattttgttaaaattcgcgttaaatttttgttaaatcagctcattttttaaccaataggccgaaatcggcaaaatcccttataaatcaaaagaatagaccgagatagggttgagtggccgctacagggcgctcccattcgccattcaggctgcgcaactgttgggaagggcgtttcggtgcgggcctcttcgctattacgccagctggcgaaagggggatgtgctgcaaggcgattaagttgggtaacgccagggttttcccagtcacgacgttgtaaaacgacggccagtgagcgcgacgtaatacgactcactatagggcgaattgaaggaaggccgtcaaggccgcattaattaatacgactcactatagggg

**pK2Cam-V_9A_( 3318 bp)**

**Map**

**
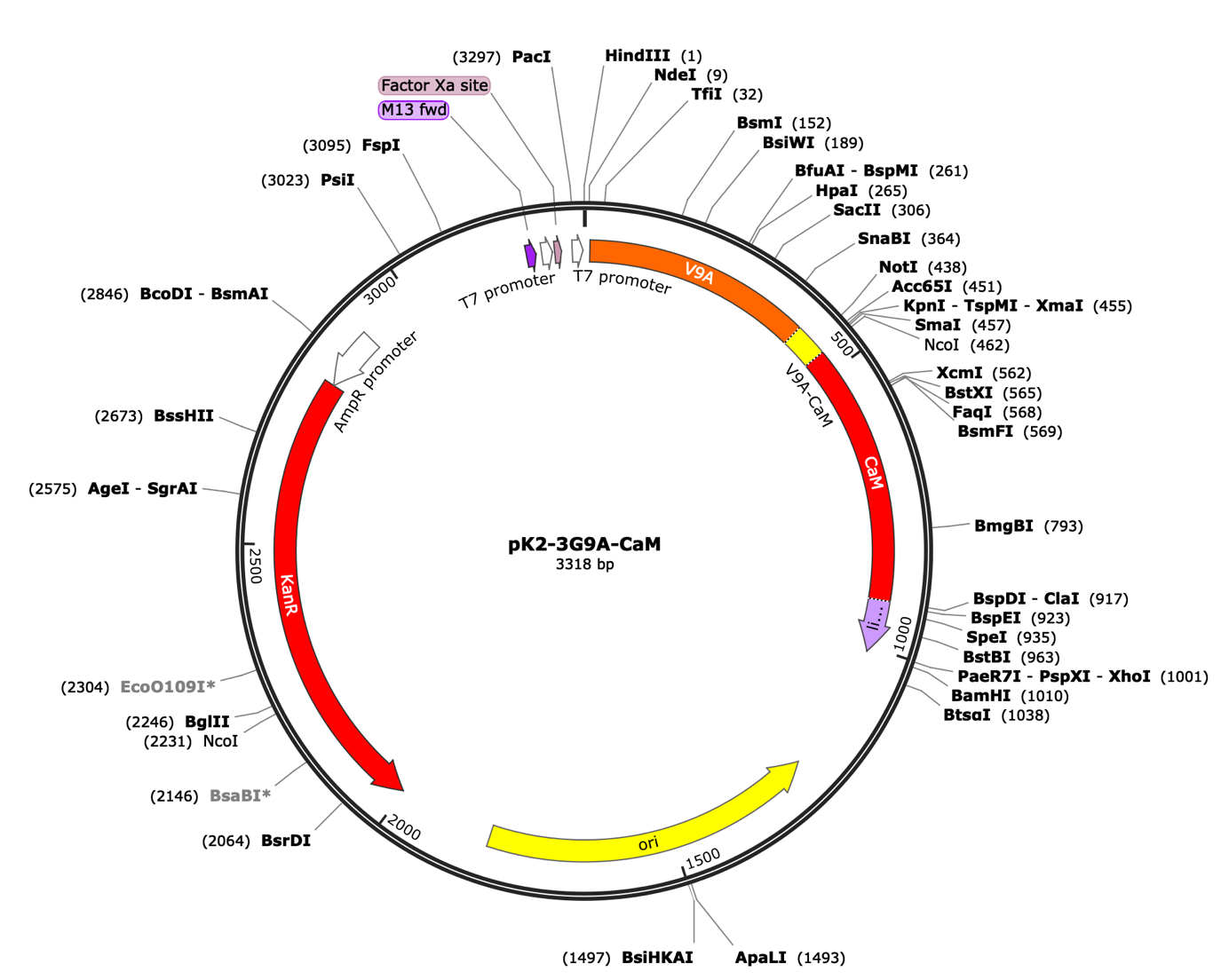
**

**DNA sequence (*Hind*III-B*amH*I fragment)**

**aagctt**gcatATGGCTGACGTTCAGCTGCAGGAATCTGGTGGTGGTTCTGTTCAGGCGGGTGGTTCTCTGCGTCTGTCTTGCGCGGCTAGCGGTGACACCTTCTCTTCTTACTCTATGGCGTGGTTCCGTCAGGCGCCGGGTAAAGAATGCGAACTGGTTTCTAACATCCTGCGTGACGGTACTACCACGTACGCCGGCTCTGTTAAAGGTCGTTTCACCATCTCTCGTGACGACGCGAAAAACACCGTTTACCTGCAGATGGTTAACCTGAAATCTGAAGACACCGCGCGTTACTACTGCGCCGCGGACTCTGGTACTCAGCTGGGTTACGTTGGTGCGGTTGGTCTGTCTTGCCTGGACTACGTAATGGACTACTGGGGTAAAGGTACTCAGGTTACCGTTTCTTCTGAACCGAAAACCCCGAAACCGCAGCCAGCGGCCGCTGAAAAGGTACCCGGGTCCATGGCTAGCGCCGACCAGCTGACCGAGGAGCAGATCGCCGAGTTCAAGGAGGCCTTCTCCCTGTTCGACAAGGACGGCGACGGCACCATCACCACCAAGGAGCTGGGCACCGTCATGCGGTCCCTGGGCCAGAACCCCACCGAGGCCGAGCTTCAGGACATGATCAACGAGGTCGACGCCGACGGCAACGGCACCATCGACTTCCCCGAGTTCCTGACCATGATGGCCCGGAAGATGAAGGACACCGACTCCGAGGAGGAGATCCGGGAGGCCTTCCGGGTCTTCGACAAGGACGGCAACGGCTATATCTCCGCCGCCGAGCTGCGGCACGTCATGACCAACCTGGGCGAGAAGCTGACCGACGAGGAGGTCGACGAGATGATCCGGGAGGCCGACATCGACGGCGACGGCCAGGTCAACTATGAGGAGTTCGTCCAGATGATGACCGCCAAATCGATGTCCGGAGGTGGCACTAGTGCTTCAGGTCTGAACGACATCTTCGAAGCTCAGAAAATCGAATGGCACGAAGGCGGCACCCTCGAGTAA**ggatcc**

**pK1Cam-V_9A_-Zip (3522 bp)**

**Map**

**
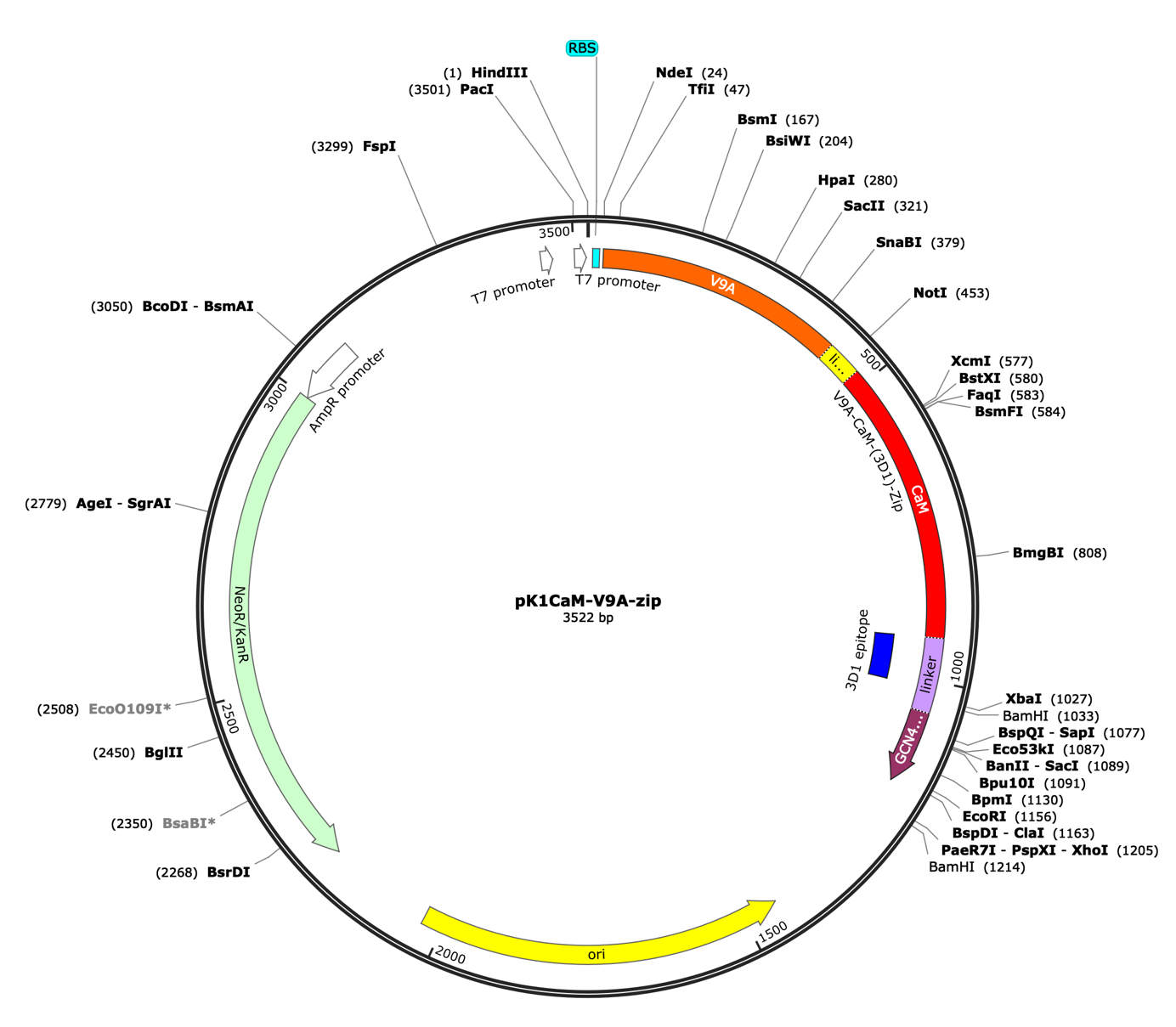
**

**DNA sequence (*Hind*III-B*amH*I fragment)**

**aagctt**ataagaaggagatatacatATGGCTGACGTTCAGCTGCAGGAATCTGGTGGTGGTTCTGTTCAGGCGGGTGGTTCTCTGCGTCTGTCTTGCGCGGCTAGCGGTGACACCTTCTCTTCTTACTCTATGGCGTGGTTCCGTCAGGCGCCGGGTAAAGAATGCGAACTGGTTTCTAACATCCTGCGTGACGGTACTACCACGTACGCCGGCTCTGTTAAAGGTCGTTTCACCATCTCTCGTGACGACGCGAAAAACACCGTTTACCTGCAGATGGTTAACCTGAAATCTGAAGACACCGCGCGTTACTACTGCGCCGCGGACTCTGGTACTCAGCTGGGTTACGTTGGTGCGGTTGGTCTGTCTTGCCTGGACTACGTAATGGACTACTGGGGTAAAGGTACTCAGGTTACCGTTTCTTCTGAACCGAAAACCCCGAAACCGCAGCCAGCGGCCGCTGAAAAGGTACCCGGGTCCATGGCTAGCGCCGACCAGCTGACCGAGGAGCAGATCGCCGAGTTCAAGGAGGCCTTCTCCCTGTTCGACAAGGACGGCGACGGCACCATCACCACCAAGGAGCTGGGCACCGTCATGCGGTCCCTGGGCCAGAACCCCACCGAGGCCGAGCTTCAGGACATGATCAACGAGGTCGACGCCGACGGCAACGGCACCATCGACTTCCCCGAGTTCCTGACCATGATGGCCCGGAAGATGAAGGACACCGACTCCGAGGAGGAGATCCGGGAGGCCTTCCGGGTCTTCGACAAGGACGGCAACGGCTATATCTCCGCCGCCGAGCTGCGGCACGTCATGACCAACCTGGGCGAGAAGCTGACCGACGAGGAGGTCGACGAGATGATCCGGGAGGCCGACATCGACGGCGACGGCCAGGTCAACTATGAGGAGTTCGTCCAGATGATGACCGCCAAATCGAAGTTCTCGCCGGATGTACTGGAAACGGTGCCGGCGTCACCCGGATTGCGGCGGCCGTCGCTGGGCGCAGTGGAACGCCACTGCAGGTCGACTCTAGAGGATCCCCGGGTACCTATCCAGCGTATGAAACAGCTGGAAGACAAAGTTGAAGAGCTCCTGAGCAAAAACTACCACCTGGAGAACGAAGTTGCGCGCCTGAAAAAACTGGTGGGTGAACGTGGGAATTCATCGATATAActaagtaatatggtgcactctcagtacaatctgctcgagtaa**ggatcc**

**pK1Cam-V_9A_-TM-Zip ( 3612 bp)**

**Map**

**
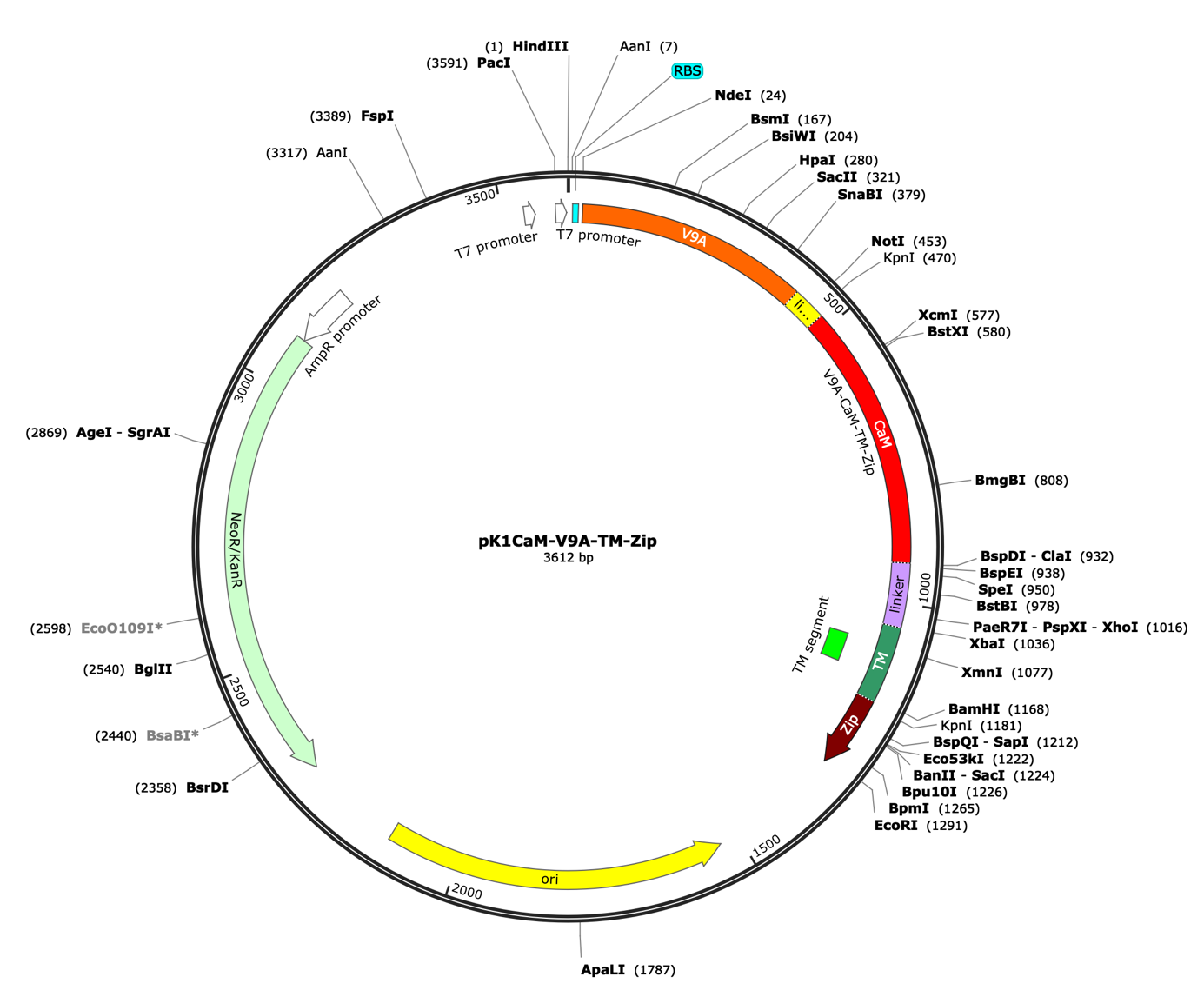
**

**Full DNA sequence**

**aagctt**ataagaaggagatatacatATGGCTGACGTTCAGCTGCAGGAATCTGGTGGTGGTTCTGTTCAGGCGGGTGGTTCTCTGCGTCTGTCTTGCGCGGCTAGCGGTGACACCTTCTCTTCTTACTCTATGGCGTGGTTCCGTCAGGCGCCGGGTAAAGAATGCGAACTGGTTTCTAACATCCTGCGTGACGGTACTACCACGTACGCCGGCTCTGTTAAAGGTCGTTTCACCATCTCTCGTGACGACGCGAAAAACACCGTTTACCTGCAGATGGTTAACCTGAAATCTGAAGACACCGCGCGTTACTACTGCGCCGCGGACTCTGGTACTCAGCTGGGTTACGTTGGTGCGGTTGGTCTGTCTTGCCTGGACTACGTAATGGACTACTGGGGTAAAGGTACTCAGGTTACCGTTTCTTCTGAACCGAAAACCCCGAAACCGCAGCCAGCGGCCGCTGAAAAGGTACCCGGGTCCATGGCTAGCGCCGACCAGCTGACCGAGGAGCAGATCGCCGAGTTCAAGGAGGCCTTCTCCCTGTTCGACAAGGACGGCGACGGCACCATCACCACCAAGGAGCTGGGCACCGTCATGCGGTCCCTGGGCCAGAACCCCACCGAGGCCGAGCTTCAGGACATGATCAACGAGGTCGACGCCGACGGCAACGGCACCATCGACTTCCCCGAGTTCCTGACCATGATGGCCCGGAAGATGAAGGACACCGACTCCGAGGAGGAGATCCGGGAGGCCTTCCGGGTCTTCGACAAGGACGGCAACGGCTATATCTCCGCCGCCGAGCTGCGGCACGTCATGACCAACCTGGGCGAGAAGCTGACCGACGAGGAGGTCGACGAGATGATCCGGGAGGCCGACATCGACGGCGACGGCCAGGTCAACTATGAGGAGTTCGTCCAGATGATGACCGCCAAATCGATGTCCGGAGGTGGCACTAGTGCTTCAGGTCTGAACGACATCTTCGAAGCTCAGAAAATCGAATGGCACGAAGGCGGCACCCTCGAGCACTGCAGGTCGACTCTAGAGGATCTAAAATTTATTCTACGTCGCTGTCTGGAAGCGATTCCGACGCTATTTATTCTTATTACTATTTCGTTCTTTATGATGCGCCTCGCGCCGGGAAGCCCTTTTACCGGCGAACGTACTTTAGCGGATCCCCGGGTACCTATCCAGCGTATGAAACAGCTGGAAGACAAAGTTGAAGAGCTCCTGAGCAAAAACTACCACCTGGAGAACGAAGTTGCGCGCCTGAAAAAACTGGTGGGTGAACGTGGGAATTCATCGTAAagatccctgggcctcatgggccttcctttcactgcccgctttccagtcgggaaacctgtcgtgccagctgcattaacatggtcatagctgtttccttgcgtattgggcgctctccgcttcctcgctcactgactcgctgcgctcggtcgttcgggtaaagcctggggtgcctaatgagcaaaaggccagcaaaaggccaggaaccgtaaaaaggccgcgttgctggcgtttttccataggctccgcccccctgacgagcatcacaaaaatcgacgctcaagtcagaggtggcgaaacccgacaggactataaagataccaggcgtttccccctggaagctccctcgtgcgctctcctgttccgaccctgccgcttaccggatacctgtccgcctttctcccttcgggaagcgtggcgctttctcatagctcacgctgtaggtatctcagttcggtgtaggtcgttcgctccaagctgggctgtgtgcacgaaccccccgttcagcccgaccgctgcgccttatccggtaactatcgtcttgagtccaacccggtaagacacgacttatcgccactggcagcagccactggtaacaggattagcagagcgaggtatgtaggcggtgctacagagttcttgaagtggtggcctaactacggctacactagaagaacagtatttggtatctgcgctctgctgaagccagttaccttcggaaaaagagttggtagctcttgatccggcaaacaaaccaccgctggtagcggtggtttttttgtttgcaagcagcagattacgcgcagaaaaaaaggatctcaagaagatcctttgatcttttctacggggtctgacgctcagtggaacgaaaactcacgttaagggattttggtcatgagattatcaaaaaggatcttcacctagatccttttaaattaaaaatgaagttttaaatcaatctaaagtatatatgagtaaacttggtctgacagttattagaaaaattcatccagcagacgataaaacgcaatacgctggctatccggtgccgcaatgccatacagcaccagaaaacgatccgcccattcgccgcccagttcttccgcaatatcacgggtggccagcgcaatatcctgataacgatccgccacgcccagacggccgcaatcaataaagccgctaaaacggccattttccaccataatgttcggcaggcacgcatcaccatgggtcaccaccagatcttcgccatccggcatgctcgctttcagacgcgcaaacagctctgccggtgccaggccctgatgttcttcatccagatcatcctgatccaccaggcccgcttccatacgggtacgcgcacgttcaatacgatgtttcgcctgatgatcaaacggacaggtcgccgggtccagggtatgcagacgacgcatggcatccgccataatgctcactttttctgccggcgccagatggctagacagcagatcctgacccggcacttcgcccagcagcagccaatcacggcccgcttcggtcaccacatccagcaccgccgcacacggaacaccggtggtggccagccagctcagacgcgccgcttcatcctgcagctcgttcagcgcaccgctcagatcggttttcacaaacagcaccggacgaccctgcgcgctcagacgaaacaccgccgcatcagagcagccaatggtctgctgcgcccaatcatagccaaacagacgttccacccacgctgccgggctacccgcatgcaggccatcctgttcaatcatactcttcctttttcaatattattgaagcatttatcagggttattgtctcatgagcggatacatatttgaatgtatttagaaaaataaacaaataggggttccgcgcacatttccccgaaaagtgccacctaaattgtaagcgttaatattttgttaaaattcgcgttaaatttttgttaaatcagctcattttttaaccaataggccgaaatcggcaaaatcccttataaatcaaaagaatagaccgagatagggttgagtggccgctacagggcgctcccattcgccattcaggctgcgcaactgttgggaagggcgtttcggtgcgggcctcttcgctattacgccagctggcgaaagggggatgtgctgcaaggcgattaagttgggtaacgccagggttttcccagtcacgacgttgtaaaacgacggccagtgagcgcgacgtaatacgactcactatagggcgaattgaaggaaggccgtcaaggccgcattaattaatacgactcactatagggg

**pK1Cam-Frb**

**Map**

**
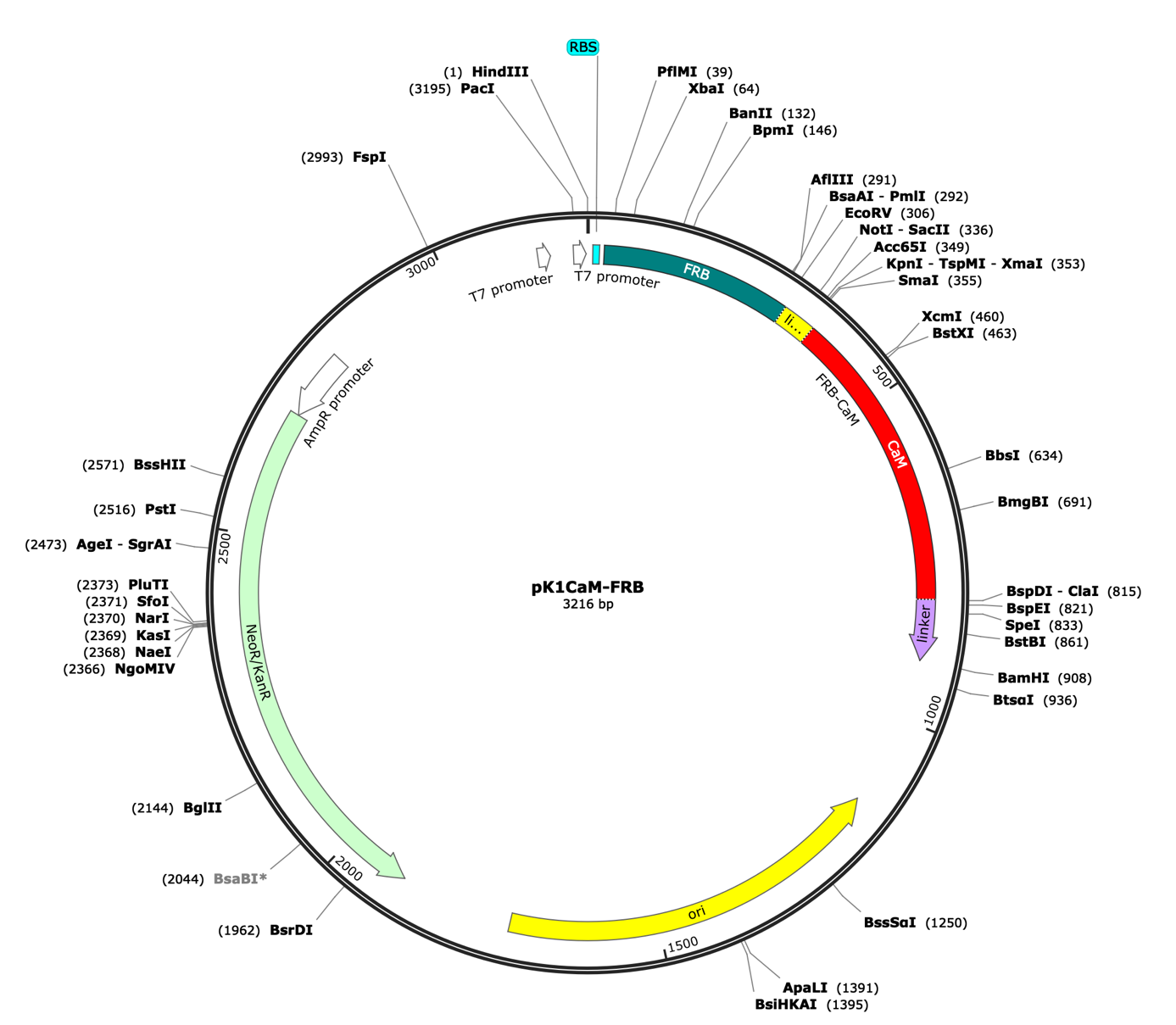
**

**DNA sequence (*Hind*III-B*amH*I fragment)**

**aagctt**ataagaaggagatatacatATGGTAGCCATCCTCTGGCATGAGATGTGGCATGAAGGTCTAGAAGAGGCCTCTCGCTTGTACTTTGGGGAGAGGAACGTCAAAGGCATGTTTGAGGTGCTGGAGCCCCTGCATGCTATGATGGAACGCGGTCCCCAGACCCTGAAGGAAACGTCCTTTAATCAGGCATATGGTCGAGATTTAATGGAGGCACAAGAATGGTGCCGAAAGTACATGAAATCAGGGAACGTCAAGGACCTCACCCAAGCCTGGGACCTCTACTATCACGTGTTCAGACGGATATCAGGCTCGAGTGGCCCGGCTAGCCCCGCGGCCGCTGAAAAGGTACCCGGGTCCATGGCTAGCGCCGACCAGCTGACCGAGGAGCAGATCGCCGAGTTCAAGGAGGCCTTCTCCCTGTTCGACAAGGACGGCGACGGCACCATCACCACCAAGGAGCTGGGCACCGTCATGCGGTCCCTGGGCCAGAACCCCACCGAGGCCGAGCTTCAGGACATGATCAACGAGGTCGACGCCGACGGCAACGGCACCATCGACTTCCCCGAGTTCCTGACCATGATGGCCCGGAAGATGAAGGACACCGACTCCGAGGAGGAGATCCGGGAGGCCTTCCGGGTCTTCGACAAGGACGGCAACGGCTATATCTCCGCCGCCGAGCTGCGGCACGTCATGACCAACCTGGGCGAGAAGCTGACCGACGAGGAGGTCGACGAGATGATCCGGGAGGCCGACATCGACGGCGACGGCCAGGTCAACTATGAGGAGTTCGTCCAGATGATGACCGCCAAATCGATGTCCGGAGGTGGCACTAGTGCTTCAGGTCTGAACGACATCTTCGAAGCTCAGAAAATCGAATGGCACGAAGGCGGCACCCTCGAGTAA**ggatcc**

**pK1Cam-Frb-Zs (4465 bp)**

**Map**

**
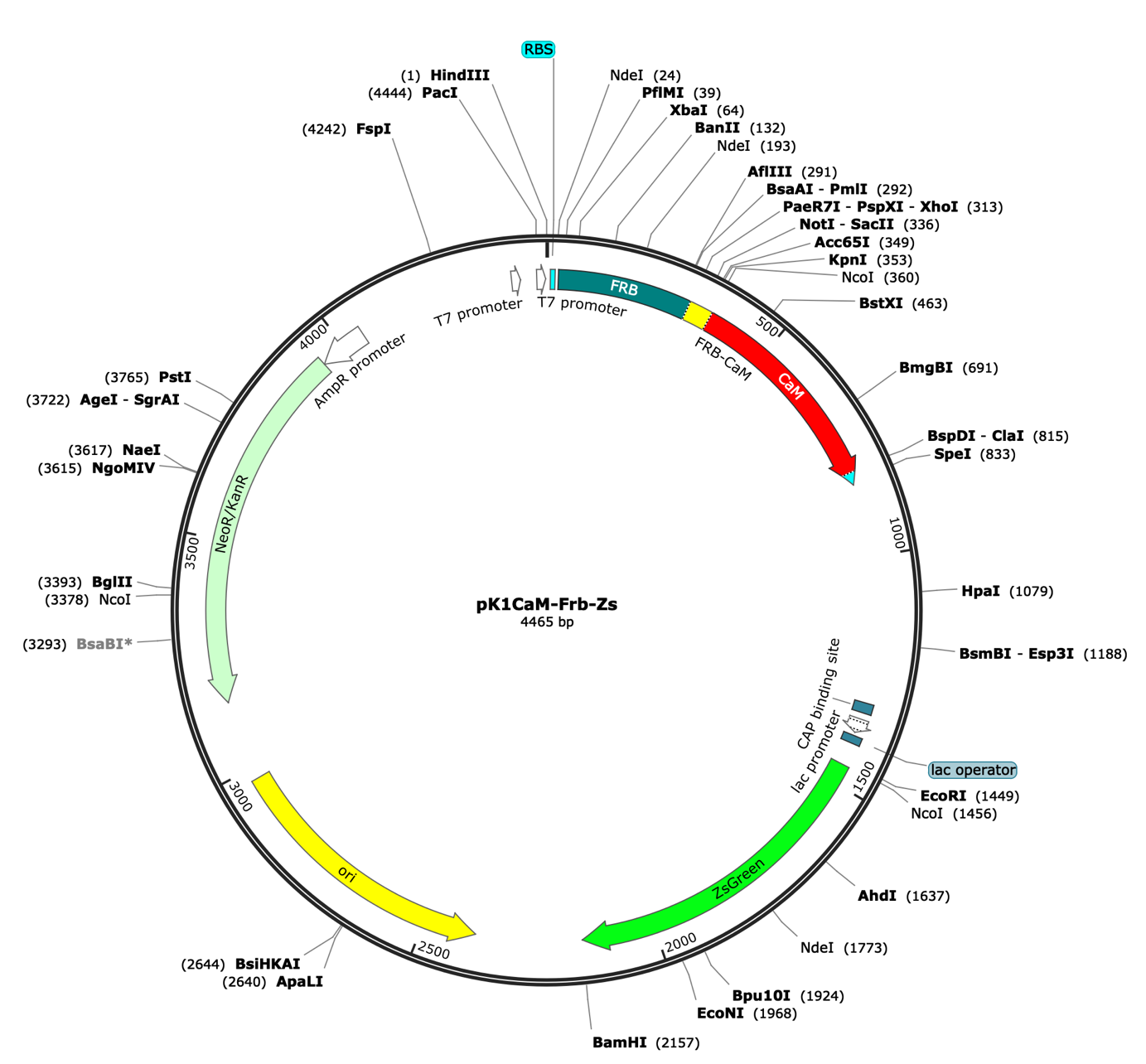
**

**DNA sequence (*Hind*III-B*amH*I fragment)**

**aagctt**ataagaaggagatatacatATGGTAGCCATCCTCTGGCATGAGATGTGGCATGAAGGTCTAGAAGAGGCCTCTCGCTTGTACTTTGGGGAGAGGAACGTCAAAGGCATGTTTGAGGTGCTGGAGCCCCTGCATGCTATGATGGAACGCGGTCCCCAGACCCTGAAGGAAACGTCCTTTAATCAGGCATATGGTCGAGATTTAATGGAGGCACAAGAATGGTGCCGAAAGTACATGAAATCAGGGAACGTCAAGGACCTCACCCAAGCCTGGGACCTCTACTATCACGTGTTCAGACGGATATCAGGCTCGAGTGGCCCGGCTAGCCCCGCGGCCGCTGAAAAGGTACCCGGGTCCATGGCTAGCGCCGACCAGCTGACCGAGGAGCAGATCGCCGAGTTCAAGGAGGCCTTCTCCCTGTTCGACAAGGACGGCGACGGCACCATCACCACCAAGGAGCTGGGCACCGTCATGCGGTCCCTGGGCCAGAACCCCACCGAGGCCGAGCTTCAGGACATGATCAACGAGGTCGACGCCGACGGCAACGGCACCATCGACTTCCCCGAGTTCCTGACCATGATGGCCCGGAAGATGAAGGACACCGACTCCGAGGAGGAGATCCGGGAGGCCTTCCGGGTCTTCGACAAGGACGGCAACGGCTATATCTCCGCCGCCGAGCTGCGGCACGTCATGACCAACCTGGGCGAGAAGCTGACCGACGAGGAGGTCGACGAGATGATCCGGGAGGCCGACATCGACGGCGACGGCCAGGTCAACTATGAGGAGTTCGTCCAGATGATGACCGCCAAATCGATGTCCGGAGGTGGCACTAGTTAAtaaatcaaattcagccgatagcggaacgggaaggcgactggagtgccatgtccggttttcaacaaaccatgcaaatgctgaatgagggcatcgttcccactgcgatgctggttgccaacgatcagatggcgctgggcgcaatgcgcgccattaccgagtccgggctgcgcgttggtgcggatatctcggtagtgggatacgacgataccgaagacagctcatgttatatcccgccgttaaccaccatcaaacaggattttcgcctgctggggcaaaccagcgtggaccgcttgctgcaactctctcagggccaggcggtgaagggcaatcagctgttgcccgtctcactggtgaaaagaaaaaccaccctggcgcccaatacgcaaaccgcctctccccgcgcgttggccgattcattaatgcagctggcacgacaggtttcccgactggaaagcgggcagtgagcgcaacgcaattaatgtgagttagctcactcattaggcaccccaggctttacactttatgcttccggctcgtatgttgtgtggaattgtgagcggataacaatttcacacaggaaacagctatgaccatgattacggattcagaattctccATGGGCCAGTCAAAGCACGGTCTAACAAAAGAAATGACAATGAAATACCGTATGGAAGGGTGCGTCGATGGACATAAATTTGTGATCACGGGAGAGGGCATTGGATATCCGTTCAAAGGGAAACAGGCTATTAATCTGTGTGTGGTCGAAGGTGGACCATTGCCATTTGCCGAAGACATATTGTCAGCTGCCTTTATGTACGGAAACAGGGTTTTCACTGAATATCCTCAAGACATAGCTGACTATTTCAAGAACTCGTGTCCTGCTGGTTATACATGGGACAGGTCTTTTCTCTTTGAGGATGGAGCAGTTTGCATATGTAATGCAGATATAACAGTGAGTGTTGAAGAAAACTGCATGTATCATGAGTCCAAATTTTATGGAGTGAATTTTCCTGCTGATGGACCTGTGATGAAAAAGATGACAGATAACTGGGAGCCATCCTGCGAGAAGATCATACCAGTACCTAAGCAGGGGATATTGAAAGGGGATGTCTCCATGTACCTCCTTCTGAAGGATGGTGGGCGTTTACGGTGCCAATTCGACACAGTTTACAAAGCAAAGTCTGTGCCAAGAAAGATGCCGGACTGGCACTTCATCCAGCATAAGCTCACCCGTGAAGACCGCAGCGATGCTAAGAATCAGAAATGGCATCTGACAGAACATGCTATTGCATCCGGATCTGCATTGCCCGGGTAA**ggatcc**

**pK1Cam-V_1K_**

**Map**

**
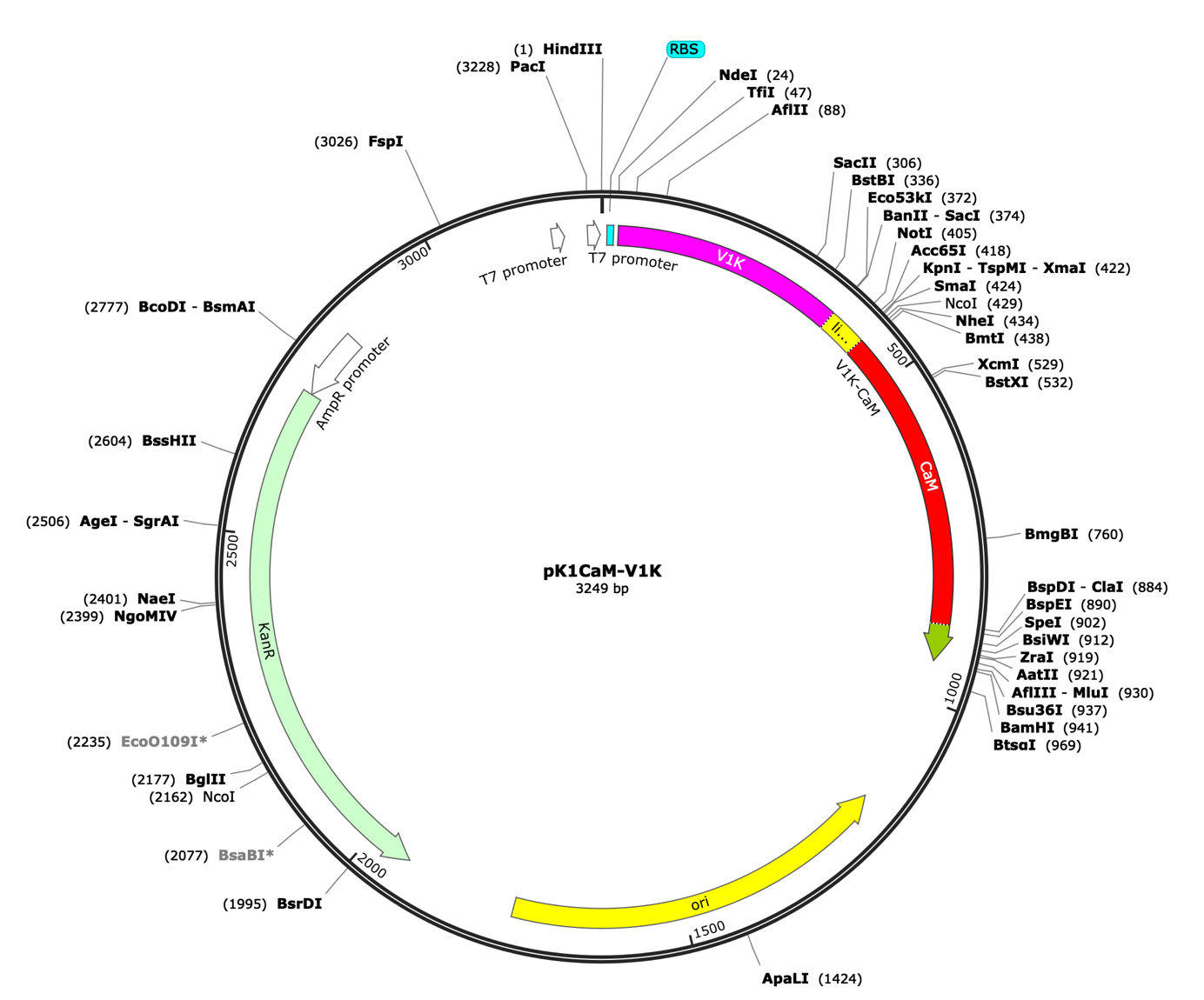
**

**DNA sequence (*Hind*III-B*amH*I fragment)**

**aagctt**ataagaaggagatatacatATGGCGCAGGTTCAGCTTGTTGAATCTGGTGGTGCGCTGGTTCAGCCGGGTGGTTCTCTGCGCTTAAGCTGCGCGGCTTCCGGTTTCCCGGTTAACCGTTACTCTATGCGTTGGTATCGTCAGGCGCCGGGTAAAGAACGTGAATGGGTTGCGGGTATGTCTTCTGCGGGTGACCGTTCTTCTTACGAAGACTCTGTTAAAGGTCGTTTCACCATCTCTCGTGACGACGCGCGTAACACCGTTTACCTGCAGATGAACTCTCTGAAACCGGAAGACACCGCGGTTTACTACTGCAACGTTAACGTTGGTTTCGAATACTGGGGTCAGGGCACCCAGGTTACCGTGAGCTCTGAACCGAAAACCCCGAAACCGCAGCCAGCGGCCGCTGAAAAGGTACCCGGGTCCATGGCTAGCGCCGACCAGCTGACCGAGGAGCAGATCGCCGAGTTCAAGGAGGCCTTCTCCCTGTTCGACAAGGACGGCGACGGCACCATCACCACCAAGGAGCTGGGCACCGTCATGCGGTCCCTGGGCCAGAACCCCACCGAGGCCGAGCTTCAGGACATGATCAACGAGGTCGACGCCGACGGCAACGGCACCATCGACTTCCCCGAGTTCCTGACCATGATGGCCCGGAAGATGAAGGACACCGACTCCGAGGAGGAGATCCGGGAGGCCTTCCGGGTCTTCGACAAGGACGGCAACGGCTATATCTCCGCCGCCGAGCTGCGGCACGTCATGACCAACCTGGGCGAGAAGCTGACCGACGAGGAGGTCGACGAGATGATCCGGGAGGCCGACATCGACGGCGACGGCCAGGTCAACTATGAGGAGTTCGTCCAGATGATGACCGCCAAATCGATGTCCGGAGGTGGCACTAGTTACCCGTACGACGTCCCAGATTACGCGTCCTAA**ggatcc**

**pK1Cam-Barstar**

**Map**

**
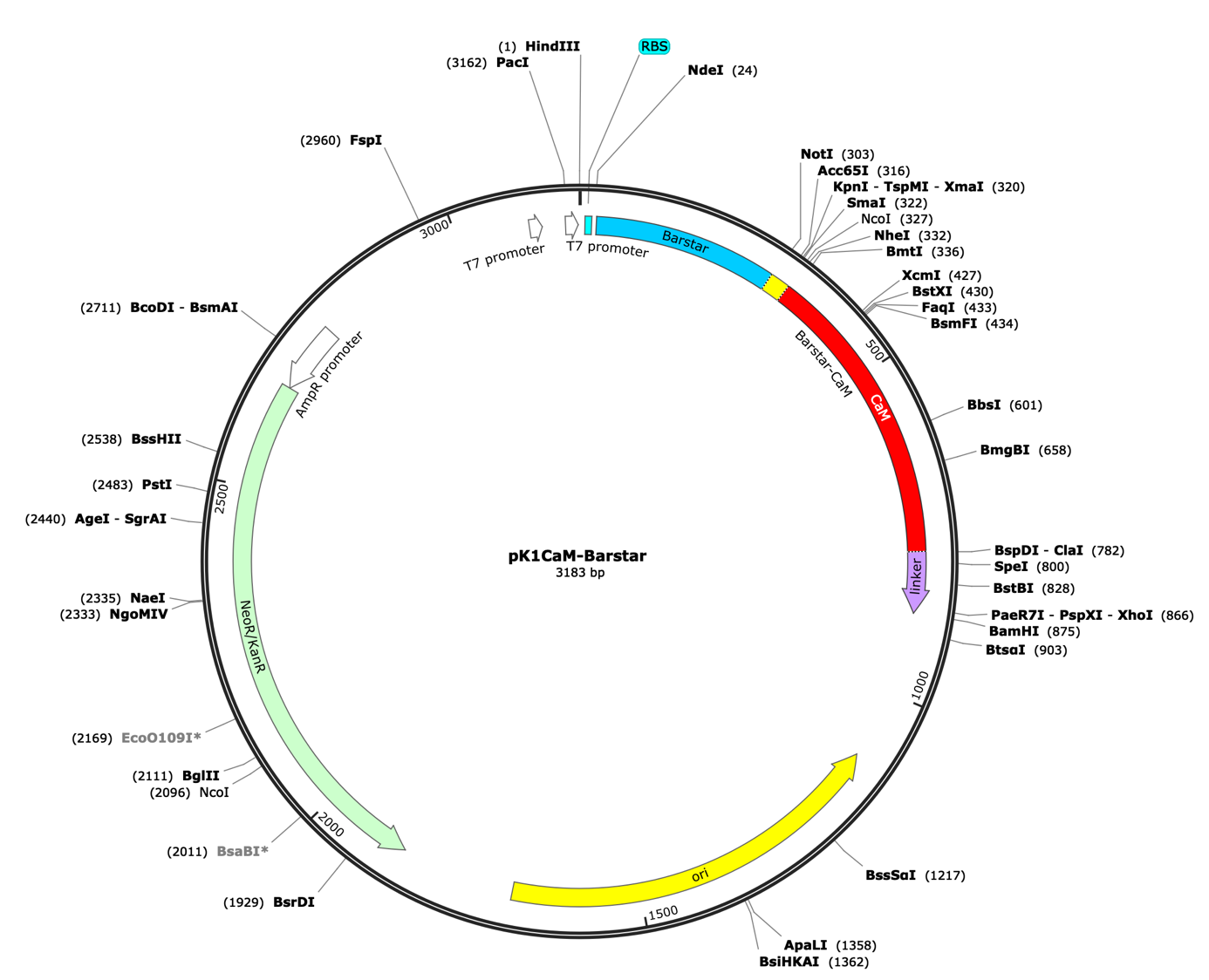
**

**DNA sequence (*Hind*III-B*amH*I fragment)**

**aagctt**ataagaaggagatatacatATGGGTAAAAAAGCAGTCATTAACGGGGAACAAATCAGAAGTATCAGCGACCTCCACCAGACATTGAAAAAGGAGCTTGCCCTTCCGGAATACTACGGTGAAAACCTGGACGCTTTATGGGATTGTCTGACCGGATGGGTGGAGTACCCGCTCGTTTTGGAATGGAGGCAGTTTGAACAAAGCAAGCAGCTGACTGAAAATGGCGCCGAGAGTGTGCTTCAGGTTTTCCGTGAAGCGAAAGCGGAAGGCTGCGACATCACCATCATACTTTCTGGTGCGGCCGCTGAAAAGGTACCCGGGTCCATGGCTAGCGCCGACCAGCTGACCGAGGAGCAGATCGCCGAGTTCAAGGAGGCCTTCTCCCTGTTCGACAAGGACGGCGACGGCACCATCACCACCAAGGAGCTGGGCACCGTCATGCGGTCCCTGGGCCAGAACCCCACCGAGGCCGAGCTTCAGGACATGATCAACGAGGTCGACGCCGACGGCAACGGCACCATCGACTTCCCCGAGTTCCTGACCATGATGGCCCGGAAGATGAAGGACACCGACTCCGAGGAGGAGATCCGGGAGGCCTTCCGGGTCTTCGACAAGGACGGCAACGGCTATATCTCCGCCGCCGAGCTGCGGCACGTCATGACCAACCTGGGCGAGAAGCTGACCGACGAGGAGGTCGACGAGATGATCCGGGAGGCCGACATCGACGGCGACGGCCAGGTCAACTATGAGGAGTTCGTCCAGATGATGACCGCCAAATCGATGTCCGGAGGTGGCACTAGTGCTTCAGGTCTGAACGACATCTTCGAAGCTCAGAAAATCGAATGGCACGAAGGCGGCACCCTCGAGTAA**ggatcc**

**pCam_VU8_ (3006 bp)**

**Map**

**
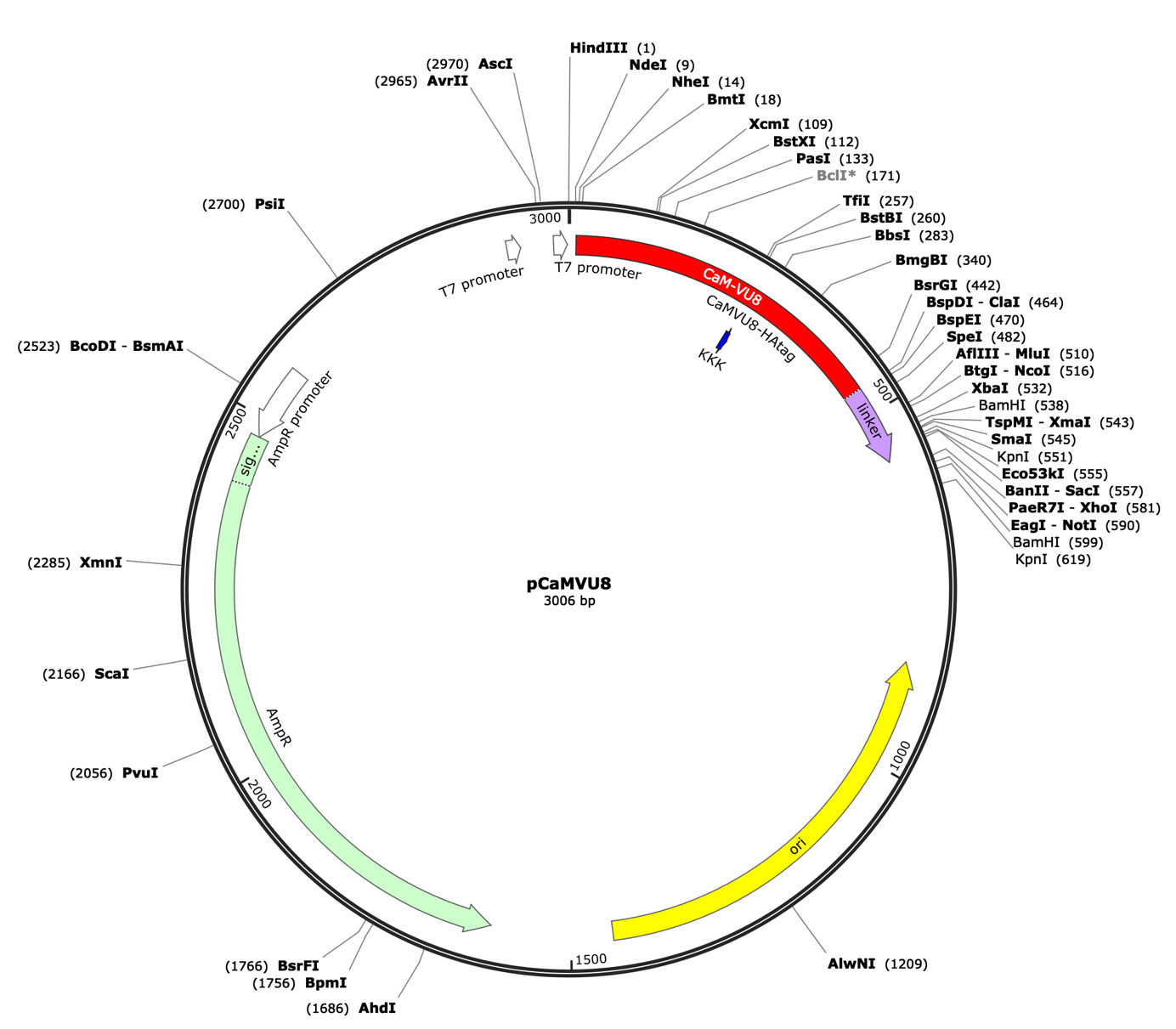
**

**Full DNA sequence**

**aagctt**gcatATGGCTAGCGCCGACCAGCTGACCGAGGAGCAGATCGCCGAGTTCAAGGAGGCGTTCTCCCTGTTCGACAAGGACGGCGACGGCACCATCACCACCAAGGAGCTGGGCACCGTCATGCGGTCCCTGGGCCAGAACCCCACCGAGGCCGAGCTTCAGGACATGATCAACGAGGTCGACGCCGACGGCAACGGCACCATCGACTTCCCCGAATTCCTGACCATGATGGCGCGCAAGATGAAGGACACCGATTCGAAGAAGAAGATCCGGGAGGCCTTCCGGGTCTTCGACAAGGACGGCAACGGCTATATCTCCGCCGCCGAGCTGCGGCACGTCATGACCAACCTGGGCGAGAAGCTGACCGACGAGGAGGTCGACGAGATGATCCGGGAGGCCGACATCGACGGCGACGGCCAGGTCAACTATGAGGAGTTTGTACAGATGATGACCGCCAAATCGATGTCCGGAGGTGGCACTAGTTACCCATACGATGTCCCAGATTACGCGTCCATGGCGCACTCGACTCTAGAGGATCCCCGGGTACCGAGCTCGAATTCATCGTAActaagtaatctcgagtagcggccgctt**ggatcc**cagaattctaggtacctcttaattaactggcctcatgggccttccgctcactgcccgctttccagtcgggaaacctgtcgtgccagctgcattaacatggtcatagctgtttccttgcgtattgggcgctctccgcttcctcgctcactgactcgctgcgctcggtcgttcgggtaaagcctggggtgcctaatgagcaaaaggccagcaaaaggccaggaaccgtaaaaaggccgcgttgctggcgtttttccataggctccgcccccctgacgagcatcacaaaaatcgacgctcaagtcagaggtggcgaaacccgacaggactataaagataccaggcgtttccccctggaagctccctcgtgcgctctcctgttccgaccctgccgcttaccggatacctgtccgcctttctcccttcgggaagcgtggcgctttctcatagctcacgctgtaggtatctcagttcggtgtaggtcgttcgctccaagctgggctgtgtgcacgaaccccccgttcagcccgaccgctgcgccttatccggtaactatcgtcttgagtccaacccggtaagacacgacttatcgccactggcagcagccactggtaacaggattagcagagcgaggtatgtaggcggtgctacagagttcttgaagtggtggcctaactacggctacactagaagaacagtatttggtatctgcgctctgctgaagccagttaccttcggaaaaagagttggtagctcttgatccggcaaacaaaccaccgctggtagcggtggtttttttgtttgcaagcagcagattacgcgcagaaaaaaaggatctcaagaagatcctttgatcttttctacggggtctgacgctcagtggaacgaaaactcacgttaagggattttggtcatgagattatcaaaaaggatcttcacctagatccttttaaattaaaaatgaagttttaaatcaatctaaagtatatatgagtaaacttggtctgacagttaccaatgcttaatcagtgaggcacctatctcagcgatctgtctatttcgttcatccatagttgcctgactccccgtcgtgtagataactacgatacgggagggcttaccatctggccccagtgctgcaatgataccgcgagaaccacgctcaccggctccagatttatcagcaataaaccagccagccggaagggccgagcgcagaagtggtcctgcaactttatccgcctccatccagtctattaattgttgccgggaagctagagtaagtagttcgccagttaatagtttgcgcaacgttgttgccattgctacaggcatcgtggtgtcacgctcgtcgtttggtatggcttcattcagctccggttcccaacgatcaaggcgagttacatgatcccccatgttgtgcaaaaaagcggttagctccttcggtcctccgatcgttgtcagaagtaagttggccgcagtgttatcactcatggttatggcagcactgcataattctcttactgtcatgccatccgtaagatgcttttctgtgactggtgagtactcaaccaagtcattctgagaatagtgtatgcggcgaccgagttgctcttgcccggcgtcaatacgggataataccgcgccacatagcagaactttaaaagtgctcatcattggaaaacgttcttcggggcgaaaactctcaaggatcttaccgctgttgagatccagttcgatgtaacccactcgtgcacccaactgatcttcagcatcttttactttcaccagcgtttctgggtgagcaaaaacaggaaggcaaaatgccgcaaaaaagggaataagggcgacacggaaatgttgaatactcatactcttcctttttcaatattattgaagcatttatcagggttattgtctcatgagcggatacatatttgaatgtatttagaaaaataaacaaataggggttccgcgcacatttccccgaaaagtgccacctaaattgtaagcgttaatattttgttaaaattcgcgttaaatttttgttaaatcagctcattttttaaccaataggccgaaatcggcaaaatcccttataaatcaaaagaatagaccgagatagggttgagtggccgctacagggcgctcccattcgccattcaggctgcgcaactgttgggaagggcgtttcggtgcgggcctcttcgctattacgccagctggcgaaagggggatgtgctgcaaggcgattaagttgggtaacgccagggttttcccagtcacgacgttgtaaaacgacggccagtgagcgcgacgtaatacgactcactatagggcgaattggcggaaggccgtcaaggcctaggcgcgccagcattaattaatacgactcactatagggg

**pCam_VU8_-V_1K_ (3315 bp)**

**Map**

**
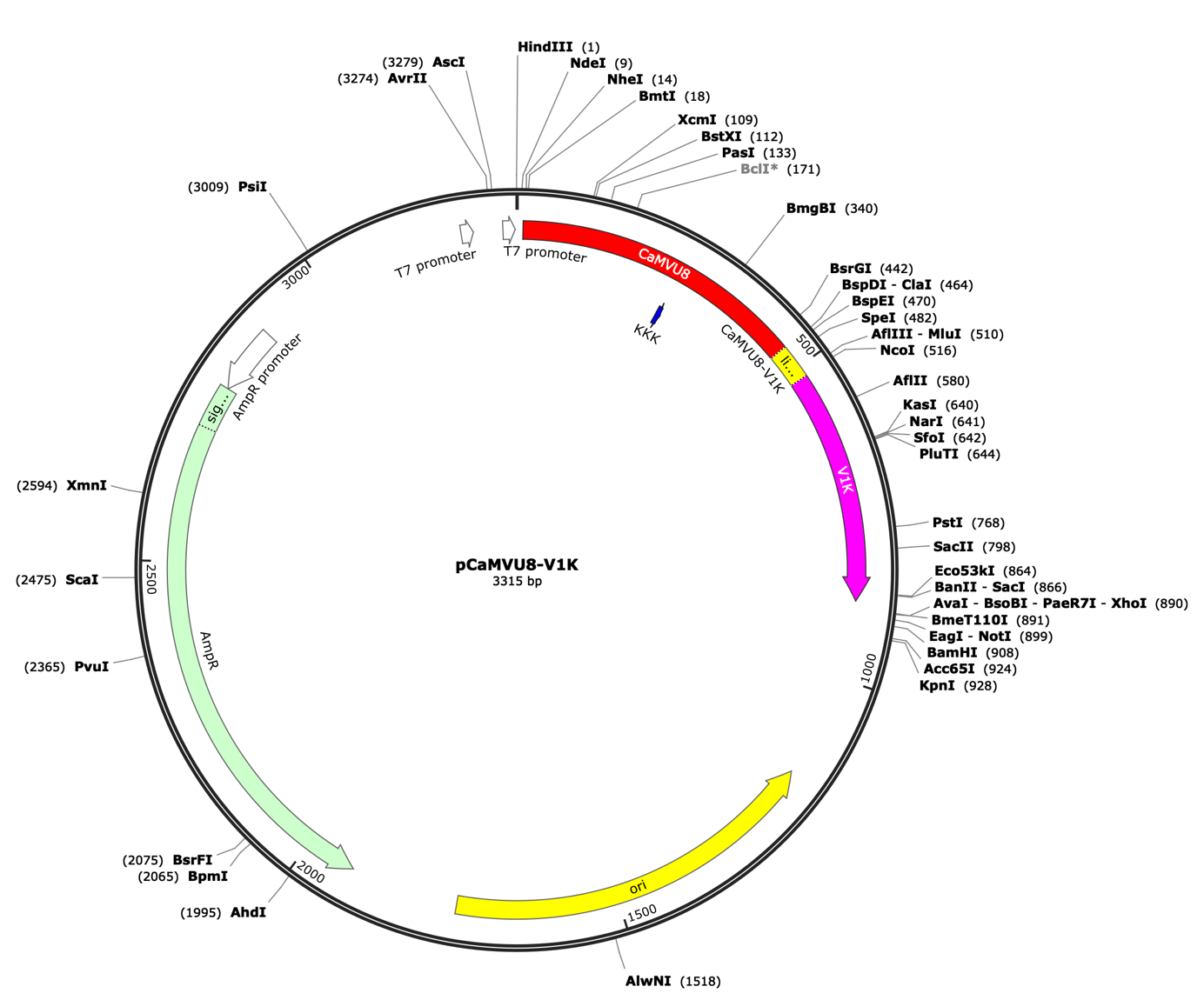
**

**Full DNA sequence**

aagcttgcATATGGCTAGCGCCGACCAGCTGACCGAGGAGCAGATCGCCGAGTTCAAGGAGGCGTTCTCCCTGTTCGACAAGGACGGCGACGGCACCATCACCACCAAGGAGCTGGGCACCGTCATGCGGTCCCTGGGCCAGAACCCCACCGAGGCCGAGCTTCAGGACATGATCAACGAGGTCGACGCCGACGGCAACGGCACCATCGACTTCCCCGAATTCCTGACCATGATGGCGCGCAAGATGAAGGACACCGATTCGAAGAAGAAGATCCGGGAGGCCTTCCGGGTCTTCGACAAGGACGGCAACGGCTATATCTCCGCCGCCGAGCTGCGGCACGTCATGACCAACCTGGGCGAGAAGCTGACCGACGAGGAGGTCGACGAGATGATCCGGGAGGCCGACATCGACGGCGACGGCCAGGTCAACTATGAGGAGTTTGTACAGATGATGACCGCCAAATCGATGTCCGGAGGTGGCACTAGTTACCCATACGATGTCCCAGATTACGCGTCCATGGCGCAGGTTCAGCTTGTTGAATCTGGTGGTGCGCTGGTTCAGCCGGGTGGTTCTCTGCGCTTAAGCTGCGCGGCTTCCGGTTTCCCGGTTAACCGTTACTCTATGCGTTGGTATCGTCAGGCGCCGGGTAAAGAACGTGAATGGGTTGCGGGTATGTCTTCTGCGGGTGACCGTTCTTCTTACGAAGACTCTGTTAAAGGTCGTTTCACCATCTCTCGTGACGACGCGCGTAACACCGTTTACCTGCAGATGAACTCTCTGAAACCGGAAGACACCGCGGTTTACTACTGCAACGTTAACGTTGGTTTCGAATACTGGGGTCAGGGCACCCAGGTTACCGTGAGCTCGAATTCATCGTAActaagtaatctcgagtagcggccgcttggatcccagaattcta**ggtacc**tcttaattaactggcctcatgggccttccgctcactgcccgctttccagtcgggaaacctgtcgtgccagctgcattaacatggtcatagctgtttccttgcgtattgggcgctctccgcttcctcgctcactgactcgctgcgctcggtcgttcgggtaaagcctggggtgcctaatgagcaaaaggccagcaaaaggccaggaaccgtaaaaaggccgcgttgctggcgtttttccataggctccgcccccctgacgagcatcacaaaaatcgacgctcaagtcagaggtggcgaaacccgacaggactataaagataccaggcgtttccccctggaagctccctcgtgcgctctcctgttccgaccctgccgcttaccggatacctgtccgcctttctcccttcgggaagcgtggcgctttctcatagctcacgctgtaggtatctcagttcggtgtaggtcgttcgctccaagctgggctgtgtgcacgaaccccccgttcagcccgaccgctgcgccttatccggtaactatcgtcttgagtccaacccggtaagacacgacttatcgccactggcagcagccactggtaacaggattagcagagcgaggtatgtaggcggtgctacagagttcttgaagtggtggcctaactacggctacactagaagaacagtatttggtatctgcgctctgctgaagccagttaccttcggaaaaagagttggtagctcttgatccggcaaacaaaccaccgctggtagcggtggtttttttgtttgcaagcagcagattacgcgcagaaaaaaaggatctcaagaagatcctttgatcttttctacggggtctgacgctcagtggaacgaaaactcacgttaagggattttggtcatgagattatcaaaaaggatcttcacctagatccttttaaattaaaaatgaagttttaaatcaatctaaagtatatatgagtaaacttggtctgacagttaccaatgcttaatcagtgaggcacctatctcagcgatctgtctatttcgttcatccatagttgcctgactccccgtcgtgtagataactacgatacgggagggcttaccatctggccccagtgctgcaatgataccgcgagaaccacgctcaccggctccagatttatcagcaataaaccagccagccggaagggccgagcgcagaagtggtcctgcaactttatccgcctccatccagtctattaattgttgccgggaagctagagtaagtagttcgccagttaatagtttgcgcaacgttgttgccattgctacaggcatcgtggtgtcacgctcgtcgtttggtatggcttcattcagctccggttcccaacgatcaaggcgagttacatgatcccccatgttgtgcaaaaaagcggttagctccttcggtcctccgatcgttgtcagaagtaagttggccgcagtgttatcactcatggttatggcagcactgcataattctcttactgtcatgccatccgtaagatgcttttctgtgactggtgagtactcaaccaagtcattctgagaatagtgtatgcggcgaccgagttgctcttgcccggcgtcaatacgggataataccgcgccacatagcagaactttaaaagtgctcatcattggaaaacgttcttcggggcgaaaactctcaaggatcttaccgctgttgagatccagttcgatgtaacccactcgtgcacccaactgatcttcagcatcttttactttcaccagcgtttctgggtgagcaaaaacaggaaggcaaaatgccgcaaaaaagggaataagggcgacacggaaatgttgaatactcatactcttcctttttcaatattattgaagcatttatcagggttattgtctcatgagcggatacatatttgaatgtatttagaaaaataaacaaataggggttccgcgcacatttccccgaaaagtgccacctaaattgtaagcgttaatattttgttaaaattcgcgttaaatttttgttaaatcagctcattttttaaccaataggccgaaatcggcaaaatcccttataaatcaaaagaatagaccgagatagggttgagtggccgctacagggcgctcccattcgccattcaggctgcgcaactgttgggaagggcgtttcggtgcgggcctcttcgctattacgccagctggcgaaagggggatgtgctgcaaggcgattaagttgggtaacgccagggttttcccagtcacgacgttgtaaaacgacggccagtgagcgcgacgtaatacgactcactatagggcgaattggcggaaggccgtcaaggcctaggcgcgccagcattaattaatacgactcactatagggg

**pCam_Cter_-V_1K_  (3087 bp)**

**Map**

**
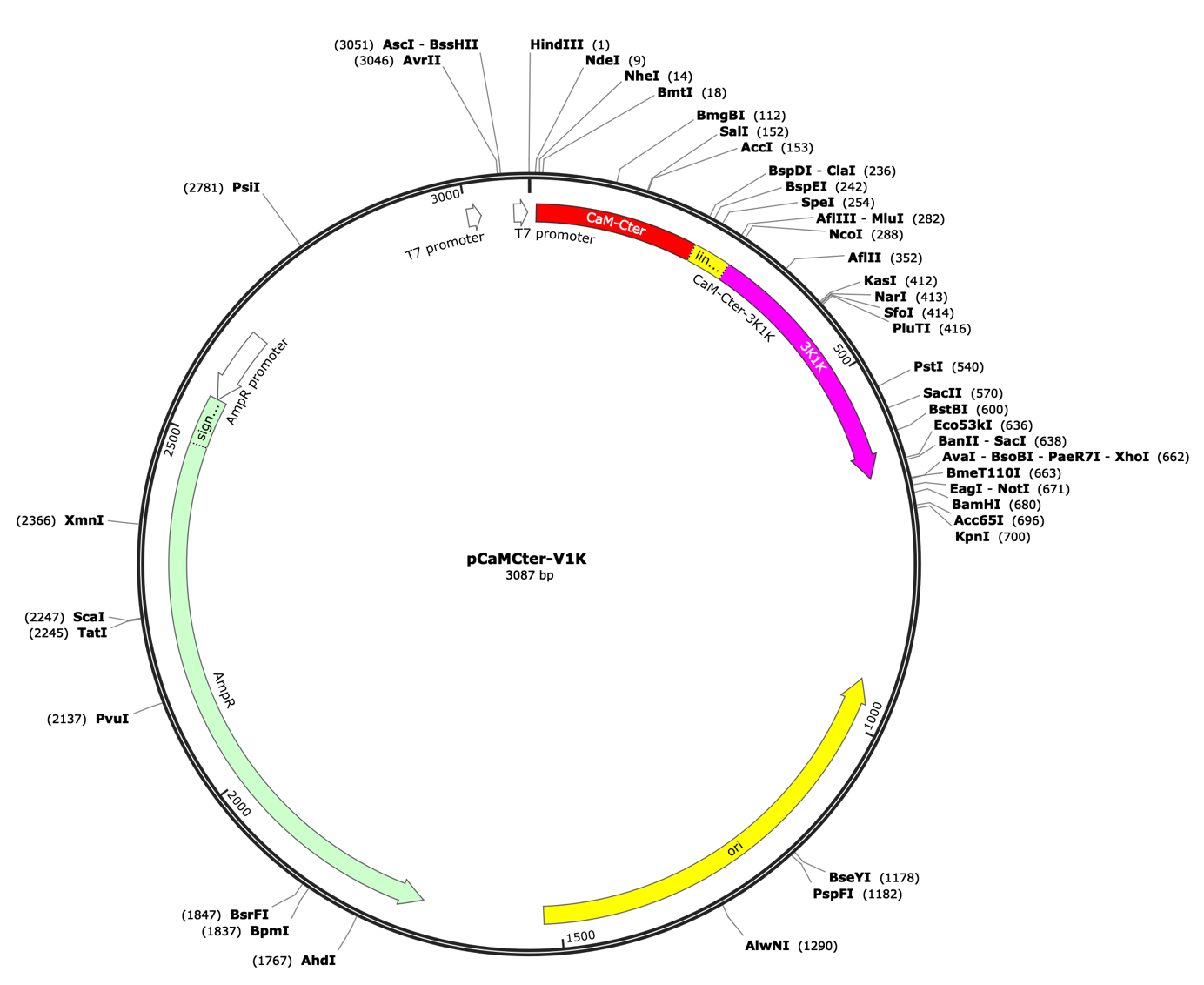
**

**Full DNA sequence**

**aagctt**gcatATGGCTAGCAAGGACACCGACTCCGAGGAGGAGATCCGGGAGGCCTTCCGGGTCTTCGACAAGGACGGCAACGGCTATATCTCCGCCGCCGAGCTGCGGCACGTCATGACCAACCTGGGCGAGAAGCTGACCGACGAGGAGGTCGACGAGATGATCCGGGAGGCCGACATCGACGGCGACGGCCAGGTCAACTATGAGGAGTTCGTCCAGATGATGACCGCCAAATCGATGTCCGGAGGTGGCACTAGTTACCCATACGATGTCCCAGATTACGCGTCCATGGCGCAGGTTCAGCTTGTTGAATCTGGTGGTGCGCTGGTTCAGCCGGGTGGTTCTCTGCGCTTAAGCTGCGCGGCTTCCGGTTTCCCGGTTAACCGTTACTCTATGCGTTGGTATCGTCAGGCGCCGGGTAAAGAACGTGAATGGGTTGCGGGTATGTCTTCTGCGGGTGACCGTTCTTCTTACGAAGACTCTGTTAAAGGTCGTTTCACCATCTCTCGTGACGACGCGCGTAACACCGTTTACCTGCAGATGAACTCTCTGAAACCGGAAGACACCGCGGTTTACTACTGCAACGTTAACGTTGGTTTCGAATACTGGGGTCAGGGCACCCAGGTTACCGTGAGCTCGAATTCATCGTAActaagtaatctcgagtagcggccgcttggatcccagaattcta**ggtacc**tcttaattaactggcctcatgggccttccgctcactgcccgctttccagtcgggaaacctgtcgtgccagctgcattaacatggtcatagctgtttccttgcgtattgggcgctctccgcttcctcgctcactgactcgctgcgctcggtcgttcgggtaaagcctggggtgcctaatgagcaaaaggccagcaaaaggccaggaaccgtaaaaaggccgcgttgctggcgtttttccataggctccgcccccctgacgagcatcacaaaaatcgacgctcaagtcagaggtggcgaaacccgacaggactataaagataccaggcgtttccccctggaagctccctcgtgcgctctcctgttccgaccctgccgcttaccggatacctgtccgcctttctcccttcgggaagcgtggcgctttctcatagctcacgctgtaggtatctcagttcggtgtaggtcgttcgctccaagctgggctgtgtgcacgaaccccccgttcagcccgaccgctgcgccttatccggtaactatcgtcttgagtccaacccggtaagacacgacttatcgccactggcagcagccactggtaacaggattagcagagcgaggtatgtaggcggtgctacagagttcttgaagtggtggcctaactacggctacactagaagaacagtatttggtatctgcgctctgctgaagccagttaccttcggaaaaagagttggtagctcttgatccggcaaacaaaccaccgctggtagcggtggtttttttgtttgcaagcagcagattacgcgcagaaaaaaaggatctcaagaagatcctttgatcttttctacggggtctgacgctcagtggaacgaaaactcacgttaagggattttggtcatgagattatcaaaaaggatcttcacctagatccttttaaattaaaaatgaagttttaaatcaatctaaagtatatatgagtaaacttggtctgacagttaccaatgcttaatcagtgaggcacctatctcagcgatctgtctatttcgttcatccatagttgcctgactccccgtcgtgtagataactacgatacgggagggcttaccatctggccccagtgctgcaatgataccgcgagaaccacgctcaccggctccagatttatcagcaataaaccagccagccggaagggccgagcgcagaagtggtcctgcaactttatccgcctccatccagtctattaattgttgccgggaagctagagtaagtagttcgccagttaatagtttgcgcaacgttgttgccattgctacaggcatcgtggtgtcacgctcgtcgtttggtatggcttcattcagctccggttcccaacgatcaaggcgagttacatgatcccccatgttgtgcaaaaaagcggttagctccttcggtcctccgatcgttgtcagaagtaagttggccgcagtgttatcactcatggttatggcagcactgcataattctcttactgtcatgccatccgtaagatgcttttctgtgactggtgagtactcaaccaagtcattctgagaatagtgtatgcggcgaccgagttgctcttgcccggcgtcaatacgggataataccgcgccacatagcagaactttaaaagtgctcatcattggaaaacgttcttcggggcgaaaactctcaaggatcttaccgctgttgagatccagttcgatgtaacccactcgtgcacccaactgatcttcagcatcttttactttcaccagcgtttctgggtgagcaaaaacaggaaggcaaaatgccgcaaaaaagggaataagggcgacacggaaatgttgaatactcatactcttcctttttcaatattattgaagcatttatcagggttattgtctcatgagcggatacatatttgaatgtatttagaaaaataaacaaataggggttccgcgcacatttccccgaaaagtgccacctaaattgtaagcgttaatattttgttaaaattcgcgttaaatttttgttaaatcagctcattttttaaccaataggccgaaatcggcaaaatcccttataaatcaaaagaatagaccgagatagggttgagtggccgctacagggcgctcccattcgccattcaggctgcgcaactgttgggaagggcgtttcggtgcgggcctcttcgctattacgccagctggcgaaagggggatgtgctgcaaggcgattaagttgggtaacgccagggttttcccagtcacgacgttgtaaaacgacggccagtgagcgcgacgtaatacgactcactatagggcgaattggcggaaggccgtcaaggcctaggcgcgccagcattaattaatacgactcactatagggg
